# Sequence dependencies and mutation rates of localized mutational processes in cancer

**DOI:** 10.1186/s13073-023-01217-z

**Published:** 2023-08-17

**Authors:** Gustav Alexander Poulsgaard, Simon Grund Sørensen, Randi Istrup Juul, Morten Muhlig Nielsen, Jakob Skou Pedersen

**Affiliations:** 1 Department of Clinical Medicine, Aarhus University, Palle Juul-Jensens Boulevard 82, 8200 Aarhus N, Denmark; 2 Department of Molecular Medicine (MOMA), Aarhus University Hospital, Palle Juul-Jensens Boulevard 99, 8200 Aarhus N, Denmark; 3 Bioinformatics Research Centre (BiRC), Aarhus University, University City 81, Building 1872, 3Rd Floor, 8000 Aarhus C, Denmark

**Keywords:** Pan-cancer, Mutational processes, Hotspots, Mutation rate

## Abstract

**Background:**

Cancer mutations accumulate through replication errors and DNA damage coupled with incomplete repair. Individual mutational processes often show nucleotide sequence and functional region preferences. As a result, some sequence contexts mutate at much higher rates than others, with additional variation found between functional regions. Mutational hotspots, with recurrent mutations across cancer samples, represent genomic positions with elevated mutation rates, often caused by highly localized mutational processes.

**Methods:**

We count the 11-mer genomic sequences across the genome, and using the PCAWG set of 2583 pan-cancer whole genomes, we associate 11-mers with mutational signatures, hotspots of single nucleotide variants, and specific genomic regions. We evaluate the mutation rates of individual and combined sets of 11-mers and derive mutational sequence motifs.

**Results:**

We show that hotspots generally identify highly mutable sequence contexts. Using these, we show that some mutational signatures are enriched in hotspot sequence contexts, corresponding to well-defined sequence preferences for the underlying localized mutational processes. This includes signature 17b (of unknown etiology) and signatures 62 (POLE deficiency), 7a (UV), and 72 (linked to lymphomas). In some cases, the mutation rate and sequence preference increase further when focusing on certain genomic regions, such as signature 62 in transcribed regions, where the mutation rate is increased up to 9-folds over cancer type and mutational signature average.

**Conclusions:**

We summarize our findings in a catalog of localized mutational processes, their sequence preferences, and their estimated mutation rates.

**Supplementary Information:**

The online version contains supplementary material available at 10.1186/s13073-023-01217-z.

## Background

Mutational signatures representing mutational processes have been identified and cataloged through analysis of large cancer genomic datasets. Some mutational processes show strong preferences for certain sequence or regional contexts, not captured by traditional mutational signature analysis. They cause variation in the mutation rate along cancer genomes with some positions displaying dramatically elevated mutation rates. These positions may manifest as mutational hotspots, which are recurrently mutated across cancer patients. Here, we use mutational hotspots identified across 2583 whole cancer genomes to discover and characterize localized mutational processes, including their mutation rate and sequence dependency.

Cancer arises through an evolutionary process within the body, where cells accumulate somatic mutations throughout life [1, 2]. Consequently, the cancer genome represents a record of the mutational processes that have shaped it since the formation of the zygote. While the majority of mutations are neutral passengers, which do not impact the cellular phenotype, some driver mutations are under recurrent positive selection across many patients and may lead to mutational hotspots [3, 4]. However, the far majority of driver hotspots reside in the protein-coding regions [5]. Therefore, we focus on non-coding regions in the PCAWG dataset [6], where few drivers are expected [7] and where we hypothesize most hotspots are explained by localized mutational processes.

Mutagenesis is a multi-step process starting with either replication error or DNA damage coupled with imperfect DNA repair and then manifests through replication as mutations in descendent cells [8, 9]. Lesions are frequently formed from endogenous processes, such as the spontaneous deamination of cytosine to uracil, and the majority are successfully repaired by the DNA damage response system [10]. Similarly, for lesions from exogenous mutagens, such as those found in tobacco smoke, the vast majority is cleared [11, 12]. Excessive lesion formation may overwhelm the DNA damage response system and result in an increased mutation rate [13, 14].

Mutational processes act with varying intensities across the genome [11, 15–23] and certain sequence motifs experience dramatically elevated mutation rates. This is for instance the case for mutations induced by UV radiation (UV), which preferentially fall in TTTCST (S = C|G) contexts as C > T mutations [21, 24–30], and certain members of the apolipoprotein B mRNA editing catalytic polypeptide-like (APOBEC) family of DNA-editing enzymes, which induce high loads of C > T and C > G mutations in TCW (W = A|T) contexts [18, 31–38]. In addition, the APOBECs specifically target single-stranded regions of DNA-level stem-loop structures to produce strand-coordinated clusters of localized hypermutation, as discovered from highly context-specific mutational hotspots [36, 38–40]. Likewise, we may study other localized mutation processes through systematic analysis of hypermutable sites and their contexts across cancer genomes.

Recent large whole-genome sequencing (WGS) datasets have powered landmark discoveries of mutational processes [6, 11, 41, 42]. Mutational signature analysis has been a key tool for disentangling the mutational processes shaping these genomes [11, 18, 22, 43]. It exploits that mutational processes are shared across patients, though with varying intensities. Using non-negative matrix factorization (NMF), recurring profiles of mutation types and contexts that represent individual mutational processes are identified and their exposure in each genome evaluated [11, 18, 43].

Given the high number of free parameters and limited data availability, mutational signature analysis was only recently expanded from considering trinucleotide (± 1 base pair [bp] neighbors) contexts to pentanucleotide contexts (± 2 bp) [22, 44]. Some mutational processes may further depend on regional properties such as chromatin organization [45–47], transcriptional activity [11, 48–50], and replication asymmetry [51, 52]. As all mutations are weighted equally, traditional signature analysis has limited power to learn the extended sequence contexts and regional preferences of rare localized mutational processes, which are generally underexplored [53].

We here aim to characterize the sequence dependency and mutation rate of localized mutational processes. We categorized all single base substitutions based on their extended sequence contexts, by considering their five bp up- and down-stream regions (11-mers). This allowed us to evaluate the mutation rate of different sequence contexts represented by individual 11-mers or sets of 11-mers. We then associated mutations with mutational signatures and their associated mutational processes. By exploiting that hotspots often pinpoint sequence contexts with generally elevated mutation rates, we identified localized mutational processes and characterized their sequence and genomic feature preferences. Based on this, we decompose the factors that increase the mutation rate in increasingly smaller parts of the genome and evaluate how these factors explain the elevation in mutation rate. We contribute a comprehensive pan-cancer catalog of localized mutational processes associated with mutational signatures.

## Methods

### Whole cancer genome dataset

We used whole-genome sequencing data from 2583 cancer patients of 37 different cancer types from The Pan-Cancer Analysis of Whole-Genomes (PCAWG) consortium [6]. The analysis was based on the full set of single nucleotide variants (SNVs), which include 343,923 coding and 41,318,716 non-coding SNVs. We focused on SNVs in the non-protein-coding part of the autosomal chromosomes. We excluded protein-coding regions to reduce potential signals of positive selection. The sex chromosomes were excluded as they include a higher rate of false SNV calls [6]. The GRCh37/hg19 reference genome was used throughout.

### Counting k-mer occurrences

First, we counted the number of k-mer instances in chromosome 1–22 using the oligonucleotideFrequency function from the Biostrings (version 2.50.2) package in R (version 3.5.1). We obtained the chromosome sequences through the R package BSgenome.Hsapiens.UCSC.hg19 (version 1.4.0). Second, we summed the counts of identical k-mers across the chromosomes. Third, to achieve strand symmetry, we collapsed reverse complementary pairs of k-mers and represented them by the sequence with a center pyrimidine (C or T) together with the total pair sum. For example, for *k* = 11, the AAAGAAGTTTC (*n*_purine_ = 5,250) and GAAACTTCTTT (*n*_pyrimidine_ = 5,495) pair was represented by GAAACTTCTTT (*n*_total_ = 10,745).

### Mutational signature annotation

#### Genome-wide mutational signature annotation

Signature posterior probabilities for the 96 different trinucleotide mutation types in each genome were calculated with SignatureAnalyzer and provided by the PCAWG consortium [22]. We downloaded 60 mutational signature annotations of all 2583 whitelisted PCAWG genomes (www.synapse.org/#!Synapse:syn11761189.6), which describe the exposure to signature X in genome Y. We classified a genome as exposed to a given mutational signature, when the signature load was equal to or above 5% of the genome’s mutation burden.

#### SNV-level mutational signature annotation

We assigned the signature posterior probabilities to each mutation, which were annotated with the most likely signature as in [7].

#### 11-mer assignment to mutational signatures

To further focus our analyses on sequence contexts targeted by the mutational signature used to define the signature-exposure cohort, we assign each individual 11-mer to the signature that best explains the SNVs found across all its 11-mer instances.

The procedure of signature assignment of 11-mers relies on three steps:

1. For each 11-mer, we calculated the mean posterior signature probabilities for SNVs across all its non-coding instances within an exposure cohort. The posterior signature probabilities are based on the patient specifc signature exposures together with the (prior) distributions over trinucleotide mutation types as specified by the mutational signatures [43]. We first averaged posterior signature probabilities of hotspot SNVs to yield a position-wise mean and then calculated the mean of all SNVs found across all instances of a given 11-mer family. This represents the average predicted probability that a given mutational signature generated the mutations.
2. We assigned each 11-mer to the signature that had the highest mean posterior probability and referred to this signature as most likely to explain its set of SNVs.
3. We identified the 11-mer families assigned to the signature in question for the given signature-exposed subset.

#### Signature similarities

We used the cosine similarity measure to calculate the similarity between two signatures defined by their mutation-type frequencies (${A}_{i}$ and ${B}_{i}$):

$$\mathrm{cosine}\;\mathrm{similarity}=\frac{\sum_{i=1}^nA_i\cdot B_i}{\sqrt{\sum_{i=1}^nA_i^2}\cdot\sqrt{\sum_{i=1}^nB_i^2}}$$

### Definition of hotspots and recurrently mutated 11-mers

We used SNV recurrence to identify 11-mers with high expected mutability. A recurrence count for hotspots was defined as the number of pan-cancer genomes with a shared position-specific SNV. We annotated 11-mers with the highest recurrence count observed across its instances. This annotation was used to further subset 11-mers into two groups: (1) 11-mers where at least one instance had a hotspot, i.e., recurrence of two or more, (2 + k-mer set) and (2) 11-mers where at least one instance had a hotspot of recurrence five or more (5 + k-mer set).

### APOBEC analysis

We grouped 11-mers into being associated with APOBEC3A, APOBEC3B, or none of the two based on their core trinucleotide sequence (TCA; APOBEC) and the 5’-neighbor being a pyrimidine (Y = C|T; APOBEC3A) or purine (R = A|G; APOBEC3B) [33]. We compared mutation rates between APOBEC3A- and APOBEC3B-motifs in 11-mers assigned to the three APOBEC signatures 2, 13, and 69. Here, the mutation rates were approximately log-normal distributed and we compared the mean log-mutation rates formally with a two-sample *t*-test in R.

### Genomic regions

We annotated the mutated 11-mer instances with the genomic region they occurred in and stratified 11-mers according to 15 different regions defined by ENCODE [54, 55]. Further characterization of repeat elements was performed using RepeatMasker (http://www.repeatmasker.org/) [56].

### Mutation rate analysis

For each 11-mer ($m$), we calculate its mutation rate (${\mu }_{m}$; SNV / Mb / patient) based on its genomic span (${n}_{m}^{\mathrm{instances}}$), the number of observed mutations (${n}_{m}^{\mathrm{SNV}}$), and the number patients in the relevant cohort (${n}^{\mathrm{patients}}$):

$${\mu }_{m}=\frac{{n}_{m}^{S N V}}{{n}^{\mathrm{patients}}\cdot {n}_{m}^{\mathrm{instances}}}$$

For a set of 11-mers (  ), we calculate the weighted average mutation rate (  ):

$${\overline{\mu }}_{\mathcal{M}}=\frac{{\sum }_{m\upepsilon \mathcal{M}}{n}_{m}^{S N V}}{{n}^{\mathrm{patients}}\cdot {\sum }_{m\upepsilon \mathcal{M}}{n}_{m}^{\mathrm{instances}}}$$

#### Statistical evaluation of mutation rate change

We evaluated 11-mer mutation rates given different genomic occurrences (family sizes; from 1 to 4,674,610) at three *p*-value thresholds (10^–2^, 10^–5^, 10^–9^) from the binomial cumulative density function (qbinom in R) with the null hypothesis of equal mutation rate (${\overline{u} }_{\mathrm{baseline}}$; 5.96 SNV/Mb/patient) in all 11-mers:

$$\begin{array}{c}{\overline{u} }_{\mathrm{baseline}}=\frac{{n}^{S N V}}{{n}^{\mathrm{patients}}\cdot {n}^{\mathrm{instances}}}\\ = \frac{41,\; 318,\; 716\; SNV}{2583 \mathrm\;{patients} \cdot 2,\; 684,\; 570,\; 106\; bp}=5.96 \frac{\mathrm{SNV}}{Mb\cdot \mathrm{patient}}\end{array}$$

To evaluate the statistical robustness of mutation rates ≥ 2 or ≥ 5 times above the expected, we computed the fraction of 11-mers that exceeded a given *p*-value threshold (10^–2^, 10^–5^, 10^–9^) at different family sizes or genomic spans in case of combined 11-mer sets.

The significance of an observed mutation rate increase from one genomic subset to another within a given signature cohort was evaluated using a binomial test (binom.test in R). The null hypothesis was that the rate did not change and hence that the mutation rate remained equal to that of the prior set. For instance, the mutation rate of the set of signature-assigned 11-mers is compared to the overall mutation rate of the signature cohort they were derived from. Likewise, the mutation rate of hotspot-associated 11-mers is compared to the signature-assigned 11-mers, and finally, the mutation rate of each functional genomic region subset is compared to the hotspot-associated 11-mers. We tested 817 mutation rate changes in total. We adjusted the *p*-values for multiple testing using Bonferroni correction, $\frac{p-\mathrm{value}}{817}$ and considered mutation rate increases with corrected *p* ≤ 0.01 as significant.

### Sequence context

Sequence information in (bits) logo plots was calculated as the Kullback–Leibler divergence between the observed and expected frequency of nucleotides at each position. The expected distribution was derived as the genome-wide autosomal distribution of nucleotides, i.e., *A* = 29.5%, *T* = 29.5%, *C* = 20.5%, and *G* = 20.5%, from R package BSgenome.Hsapiens.UCSC.hg19 version 1.4.0 using the oligonucleotideFrequency function from Biostrings version 2.54.0.

The surprise of observing nucleotide $a$ at a given position, $i$, is estimated as the Kullback–Leibler divergence (${D}_{\mathrm{KL}}$):

$${D}_{K L}\left({p}_{i}, {q}_{i}\right)=\sum_{a\upepsilon \left\{A, C, G, T\right\}}{p}_{a,i}\cdot {log}_{2}\frac{{p}_{a,i}}{{q}_{a,i}},$$

where ${p}_{a,i}$ is the observed frequency and ${q}_{a,i}$ is the expected frequency of nucleotide $a$ in position $i$. The divergence is visualized using a logo plot with letter $heigh{t}_{a,i}$ proportional to letter frequency, ${p}_{a,i}$, and divergence, ${D}_{K{L}}({p}_{i},{q}_{i})$, in that position:

$${height}_{a,i}={p}_{a,i}\cdot {D}_{K L}({p}_{i,}\; {q}_{i})$$

#### Background 11-mer sets derived from mutational signatures

We use mutational signatures as proxies for mutational processes and specify signature target regions through sets of signature-assigned 11-mers, as described above. Given that mutational signature models capture trinucleotide contexts (the mutated nucleotide and its neighbors), they will induce a distribution of nucleotide contexts in the signature-assigned 11-mer sets. To help identify interesting mutational sequence contexts and evaluate their significance given the signature-induced context composition, we construct signature-specific background sets of 11-mers, which are then provided to the pLogo [57] and kpLogo [58] methods to define appropriate background nucleotide composition models.

To construct the 11-mer background sets for a specific mutational signature ($s$), we ask how mutations would be distributed when generated according to the signature’s trinucleotide (neighbor-dependent) mutation type distribution (*n* = 96) across the (strand-symmetric) 11-mers of the genome (*n* = 2,097,090). To achieve this, we introduce a function ($trinuc$), which extracts the reference trinucleotide from a given neighbor dependent mutation type ($u$) or from the center of an 11-mer ($m$). Based on the given signature’s mutation-type probabilities, $P(u|s)$, and the genomic frequency of a given 11-mer, $P(x)$, and its corresponding trinucleotide, $P(trinuc(m))$, we can calculate the signature specific probability that a mutation falls in a given 11-mer compatible with the mutation type (i.e. $trinuc(u) =trinuc(m)$):

$$P\left(u\;in\;m\vert s\right)=\frac{P\left(u\vert s\right)P(m)}{P(trinuc\left(m\right))}$$

As our analysis only considers whether a mutation has happened and not its type, we further calculate the marginal probability of a given 11-mer being mutated by summing over all compatible mutation types:

$$P \left(m|s\right)= \sum_{\left\{u:trinuc\left(u\right)=trinuc(m)\right\}}P(u\; in\; m|s)$$

## Results

### Baseline mutation rate across families of 11-mers

**Fig. 1:**
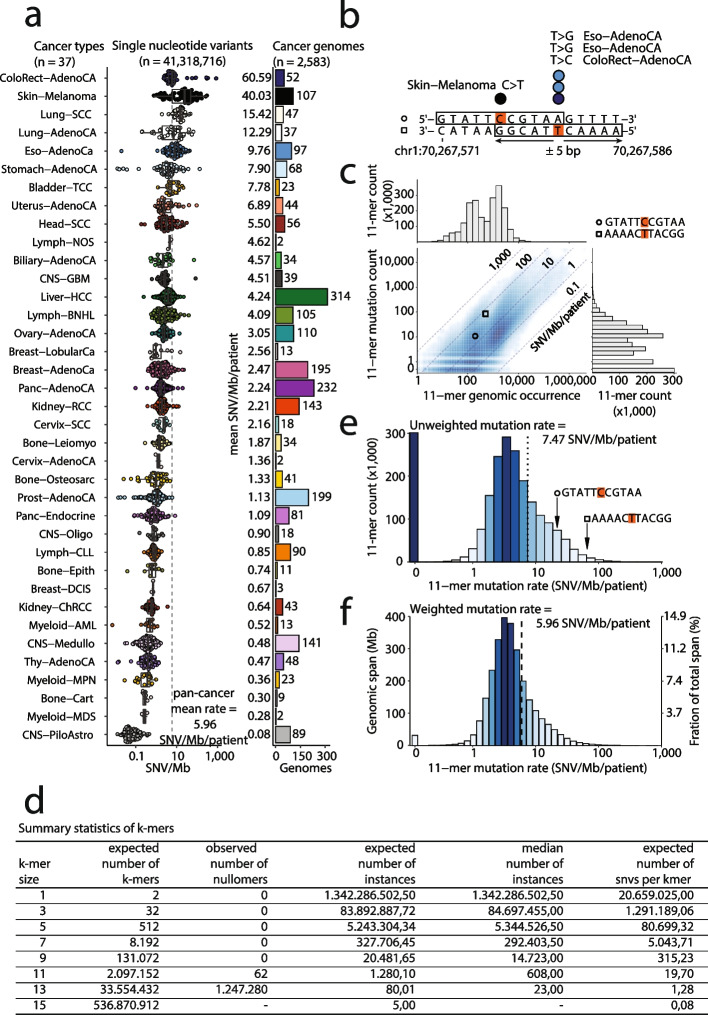
Mutation data and differential mutability of 11-mers. **a** The mutation rate of non-coding mutations (dots and boxplot) and the number of cancer genomes (bar chart) grouped and colored by cancer type. Figure 1a provides the color legend for cancer types for all figures. **b** Illustration of singleton and hotspot single nucleotide variants (SNVs). Strand symmetry is assumed in the analysis and mutated base pairs are represented by their reference pyrimidines (orange). Mutations are annotated with the ± 5 bp nucleotide context on the strand of the mutated pyrimidine and represented as 11-mers (framed) in the downstream analysis. **c** The distribution of 11-mer occurrences in the reference genome (x-axis) versus pan-cancer mutation count in 11-mers (y-axis) portrayed in a density cloud (*n* = 2,097,090). Diagonal lines represent mutation rates. Marginal plots show the distribution of 11-mer occurrences (top) and mutation count (right). **d** K-mer summary statistics given different sequence lengths (k). **e** The distribution of 11-mer mutation rates. Each 11-mer contributes a count on the y-axis. **f** The distribution of 11-mer mutation rates as a function of their genomic span. Each 11-mer contributes with its genomic occurrences to the genomic span on the y-axis. The secondary y-axis shows the fraction of the total genomic span (100%; 2,684,570,106 bp)

To estimate mutation rates, we initially identified 343,923 coding and 41,318,716 non-coding SNVs from the PCAWG set of 2583 whole cancer genomes [6] (Fig. 1a). Our analyses focused on the non-coding SNVs, which occur at an overall mutation rate of 5.96 SNV/Mb/patient (baseline mutation rate) across the dataset.

To investigate the sequence dependency of mutations, we classified all genomic positions (*n* = 2,684,570,106) by their 5 bp up- and downstream context, which we considered as 11-mer sequences (Fig. 1b). To achieve strand symmetry, base pairs were viewed from the strand that contains the pyrimidine. Hence, 11-mer sequences representing genomic positions with a purine on the plus strand were reverse complemented.

The human reference genome (hg19) contains 2,097,090 unique strand-symmetric 11-mer sequences. Each 11-mer represents a family of concrete instances along the genome, with some families much larger than others. Unless otherwise stated, 11-mers will refer to the 11-mer families. For each family, we calculated the average mutation rate per patient across the dataset, for example, the AAAACTTACGG family harbors 85 SNVs across 500 instances considering all 2583 patients, which result in a mutation rate of 65.8 SNV/Mb/patient (Fig. 1c).

We based our analysis on k-mers of length 11 as they provided an extended mutational context while allowing for a sufficient number of expected mutations for each k-mer family to achieve useful mutation rate estimates (“Methods”). If all 11-mers were equally common and mutations uniformly distributed, we would expect to observe 19.7 SNVs across 1280 instances for each possible 11-mer. In general, the choice of k-mer length represents a tradeoff between context resolution and robustness of mutation rate estimates (Fig. 1d and Additional file 1: Table S1). For example, using a k-mer length of 13 results in a much lower expected number of instances (*n* = 80) and SNVs (*n* = 1.3) per family. Additionally, 3.9% (*n* = 1,247,280) of 13-mers are absent from the reference genome. Larger k-mers will thus result in more uncertain rate estimates including many more k-mers with no observed mutations, while shorter k-mers will provide less sequence context resolution.

### Highly variable 11-mer mutation rate

We observed a mean mutation rate of 7.47 SNV/Mb/patient across all families of 11-mers, with a high degree of variation (sd = 13.1). Fourteen percent of 11-mers (*n* = 300,837) harbor no mutations at all, while the rest (85.7%; *n* = 1,796,253) have mutation rates ranging from 0.12 to 774 SNV/Mb/patient, displaying a 6492-fold difference. This high variation illustrates the inherent heterogeneity of the mutation rate of 11-mers across the genome (Fig. 1d).

When we weigh 11-mer mutation rates by their number of genomic instances, we recover the baseline mutation rate (5.96 SNV/Mb/patient; Fig. 1e). In the downstream analyses, we focus on these weighted mutation rates across sets of 11-mers to allow comparison between different genomic subsets.

**Fig. 2:**
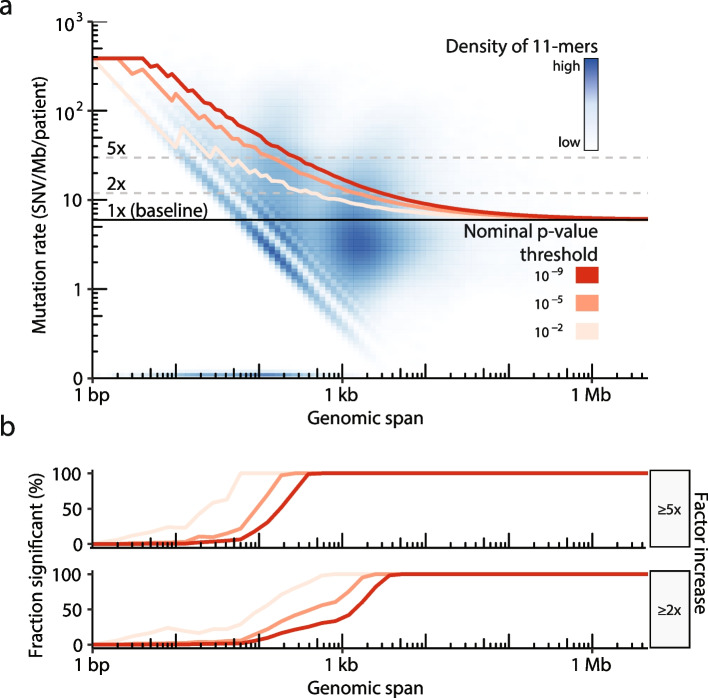
Uncertainty of 11-mer mutation rates. **a** Density of all genomic 11-mers (blue-scale) according to their genomic spans (x-axis) and mutation rates (y-axis). The mean mutation rate of the dataset (5.96 SNV/Mb/patient) is indicated by a solid line (baseline). Dashed lines indicate a 2- and 5-factor mutation rate increase. Colored curves (shades of red) represent the nominal *p*-value thresholds for a given 11-mer mutating at a significantly elevated rate compared to the baseline, with 11-mers above and to the right considered significant at the given level. If all 2,097,090 11-mer mutation rates were tested separately, the nominal *p*-value threshold of 10^–9^ (red) would provide a conservative bound for significance after (Bonferroni) multiple testing correction. In the downstream analysis of this study, we focus on a total of 817 combined sets of 11-mers, with extended spans compared to individual 11-mers. The nominal *p*-value threshold of 10^–5^ (organge) conservatively defines the region of mutation rates and spans where they would be significant after multiple testing correction. **b** The expected fraction of 11-mer sets achieving significance when the mutation rate is increased by a factor of two (top) or by a factor of five (bottom) as a function of their genomic spans. Color-coding and interpretation of p-value thresholds as in panel **a**

Some of the variation in mutation rates is a consequence of the sampling variation caused by differences in 11-mer family sizes (i.e., their genomic spans) (Fig. 1e). In general, larger genomic spans result in smaller sampling variations and an increased significance of a given mutation rate increase. For instance, 11-mers with a combined genomic span of 100 bp will require a mutation rate increase of five times (5 ×) the baseline to become statistically significant (*p* ≤ 10^–2^; binomial test) with high power (fraction 100%), while 11-mers with genomic spans of 1 kb require only 2 × increases to achive the same level of significance (Fig. 2a). Correspondingly, 100% of 11-mers with a 5 kb genomic span and mutation rates increases of more than 2 × have *p*-values below 10^–9^ and are thus highly unlikely to be significantly influenced by sampling variation (Fig. 2b). If we correct for the total number of 11-mers (Bonferroni correction on 2 million tests), the span of 11-mer sets need to increase to obtain significant mutation rate increases. Mutation rate increases of ≥ 2 × baseline for 11-mers and sets of 11-mers with ≥ 5 kb genomic span will generally be robust to sampling variation, and rate variation affecting large genomic spans may be explained by highly mutable extended nucleotide contexts [30, 59–61].

For individual 11-mers, the observed number of instances per family range widely from 0 to 4,674,610 (median 608; Fig. 2c and Additional file 1: Table S1). There are 62 (0.003%) 11-mers that are not present in the reference genome. We found 300,837 non-mutated 11-mers (14.3%), which span 31 Mb of the genome, while there were 32,080 (1.5%) highly mutated 11-mers (≥ 50 SNV/Mb/patient), which span 17 Mb. Overall, 104,992 (5.0%) 11-mers with a combined span of 254 Mb had significantly elevated mutation rates (*p* ≤ 10^–2^ after Bonferroni correction).

The mutational properties of individual 11-mers are not the main focus of this paper. Rather, for the downstream analysis, we used 11-mers as a tool for characterizing the mutational properties of sets of 11-mers associated with individual mutational processes, hotspots, and genomic regions. Depending on the size of the analyzed cohort, large genomic spans with substantially increased mutation rates usually become significant (Fig. 2b), which we then attribute to mutable sequence contexts [30, 59–61] of the included 11-mers.

### Assignment of mutated 11-mers to mutational processes

We next sought to identify and group mutated 11-mers by their underlying mutational processes to characterize their relative mutation rates and extended sequence preferences. As a proxy for mutational processes, we used the 60 mutational signatures from the PCAWG consortium, generated using the SignatureAnalyzer software [11, 18, 22, 43].

**Fig. 3:**
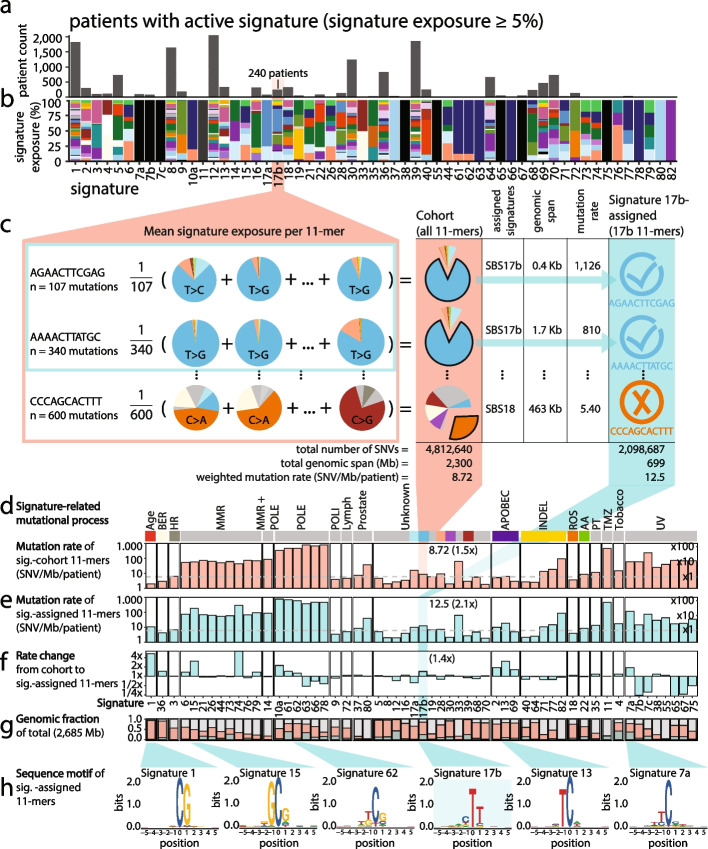
Assignment of cohorts and 11-mers to mutational signatures. **a** Stratification of genomes based on mutational signature load into 60 so-called activity cohorts. Each activity cohort comprises a number from 0 to 2049 genomes (median 48). The cohort with active signature 17b has 240 patients. **b** Fraction of cancer types in each activity cohort. Cancer type color legend can be found in Fig. 1a. **c** Each mutation has a posterior probability distribution of possible explanatory signatures (piechart). The average posterior probability distribution for an 11-mer is used to evaluate its most likely explanatory signature. On average, the mutations in 11-mers AGAACTTCGAG and AAAACTTATGC are most like explained by signature 17b, while mutations in CCCAGCACTTT are most likely explained by signature 18. All mutated 11-mers in the cohort are used as a background (red column). All 11-mers with signature 17b as the most likely signature make up a set of signature 17b-assigned 11-mers used for further analyses (blue column). The color legend for the piecharts can be found in panel **d**. **d** Color legend for signature association (top). Mutation rate of mutated 11-mers within each activity cohort (bottom). The mutation rates (left y-axis) are compared to the pan-cancer mutation rate (5.96 SNV/Mb/patient; grey dashed line) and differences are represented as a fold-change (right y-axis). **e** Mean mutation rate of each signature-assigned 11-mer set (blue). The mutation rates (left y-axis) are compared to the global mutation rate (5.96 SNV/Mb/patient; grey dashed line) and represented as a fold-change (right y-axis). **f** Fold-change from activity cohort mutation rate to signature-assigned 11-mer sets mutation rate. **g** Fraction of the genome spanned by 11-mers selected in each analysis step. **h** Sequence information content visualized by bit logo plots. The surprise (information) of observing a nucleotide is measured in bits derived from the Kullback–Leibler divergence with the reference genome as a background (A = 29.5%; C = 20.5%; G = 20.5%, T = 29.5%; “Methods”)

Cancer genomes were grouped into cohorts with shared signature exposure (≥ 5% exposure; “Methods”), allowing us to study 11-mers across genomes with potential for shared mutational processes. We obtained 57 signature-exposed cohorts (Fig. 3a) each representing between 1 and 2049 genomes inferred to share a mutational process either pan-cancer or cancer type-specific (Fig. 3b). As the mutation burden of a cancer genome is typically explained by multiple signatures, the signature-exposed cohorts overlap in their ascribed genomes. Consequently, some genomes are members of several signature-exposed cohorts.

Some processes were exclusive to distinct tissues, such as UV exposure to the skin (signature 7a; 89 melanoma genomes), while other widely active processes of unknown etiologies, such as signature 17b, possibly related to gastrointestinal cancer or 5-fluorouracil exposure, were found across many cancer types (240 genomes, 13 cancer types). The intrinsic clock-like process of 5-methylcytosine deamination (signature 1) was active in the far majority (70.7%) of all genomes (1825 genomes, 37 cancer types).

From the 11-mers in each signature-exposed cohort (Fig. 3c), we computed the cohort-wise mutation rates (Fig. 3d). As expected, we observed that some of these signature-exposed cohorts had much elevated mutation rates compared to the pan-cancer baseline mutation rate, including cohorts defined by signatures associated with mismatch repair (MMR; mean of 63.5 ± standard deviation of 13.2 SNV/Mb/patient; 10.7 × the pan-cancer baseline), POLE (579.5 ± 183.9 SNV/Mb/patient; 97.4 ×), and UV (79.2 ± 66.6 SNV/Mb/patient; 13.3 ×) (Fig. 3d and Additional file 1: Fig. S1).

For each signature-exposed cohort, we next identified the subset of 11-mers that can be explained primarily by the defining signature. We use the probabilities that individual signatures generated the observed mutations to assign 11-mers to their explanatory mutational process (Fig. 3c; “Methods”).

We characterized the mutation rates of these signature-assigned 11-mers and found that the rates of a number of signatures were much higher than both the baseline (Fig. 3e and Additional file 1: Fig. S1) and the average across the signature cohorts they were derived from (Fig. 3f), most notably signatures related to UV (7a), APOBEC (13), MMR deficiency (74), and POLE deficiency (10a). The 11-mers ascribed to signatures of age, MMR, POLE, and APOBEC generally spanned low fractions of the genome (2–8%). While the genomic spans of 11-mers assigned to, e.g., tobacco (42%), UV (37%), and signature 17b (26%) were much larger (Fig. 3g). We evaluated sequence preferences as logo plots relative to the genomic base composition (Fig. 3h) and relative to the composition dictated by the mutational signature (“Methods”; Additional file 1: Fig. S1 and S2). We observed that the base composition in the signature-assigned 11-mer sets mostly recapitulated the composition expected from the signature.

#### The APOBEC processes

For the APOBEC-related mutational signatures 2, 13, and 69, we further tried to evaluate the relative contributions of APOBEC3A (A3A), which preferentially targets YTCA contexts, and APOBEC3B (A3B), which preferentially targets RTCA [33].

Signature 2-exposed genomes (*n* = 303) had mutation rate increases from all non-APOBEC-targets (4.4 SNV/Mb/patient; 1958 Mb) to A3A-targets (5-fold; 23.1 SNV/Mb/patient; 63 Mb) and A3B-targets (4-fold; 16.2 SNV/Mb/patient; 40 Mb). Similarly, signature 13-exposed genomes (*n* = 330) had comparable mutation rate increases from all non-APOBEC-targets (5.3 SNV/Mb/patient; 2159 Mb) to A3A-targets (5-fold; 26.7 SNV/Mb/patient; 63 Mb) and A3B-targets (4-fold; 19.5 SNV/Mb/patient; 41 Mb). In contrast, signature 69-exposed genomes (*n* = 468) had much lower mutation rate changes from non-APOBEC-targets (2.9 SNV/Mb/patient; 2,087 Mb) to A3A- (2-fold; 4.7 SNV/Mb/patient; 60 Mb) and A3B-targets (2-fold; 6.0 SNV/Mb/patient; 39 Mb).

The mutation rates of A3A-targets were significantly different from A3B-targets within all three APOBEC cohorts (signature 2: *p* = 10^–307^; signature 13: *p* = 10^–280^; signature 69: *p* = 10^–275^; two-sample *t*-tests). Consistent with Chan et al. 2015 [33], we find that A3A induces mutations with higher rates and in larger fractions of the genome than A3B, which further establishes A3A as the major mutator of the two.

### Hotspots identify 11-mers with high mutation rates

**Fig. 4:**
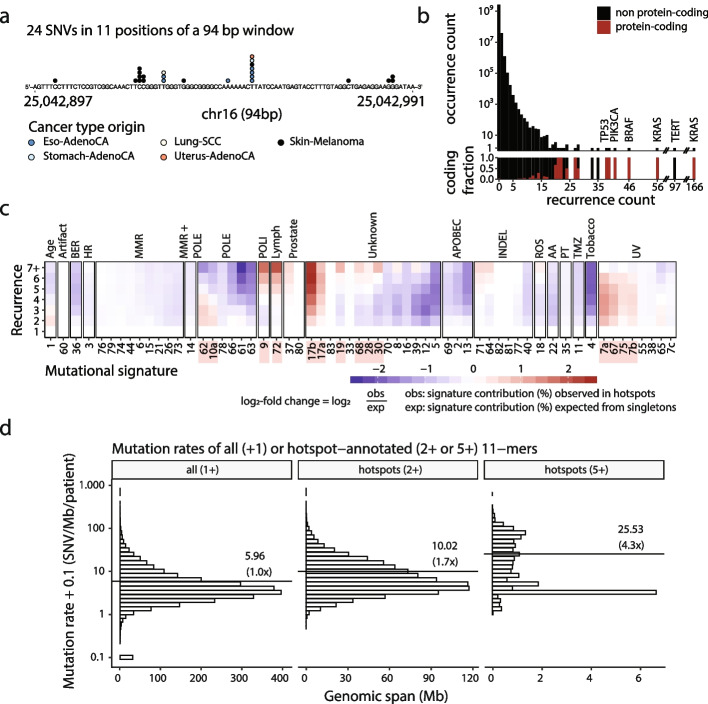
Hotspot overview and identification of enriched localized mutational processes. **a** Examples of pan-cancer recurrent and singleton SNVs in a 94-bp window on chromosome 16. SNVs are colored by cancer type. **b** Hotspot recurrence counts (x-axis) and frequency in counts (y-axis; top) with the proportion (bottom) of positions in protein-coding (red) or non-protein-coding regions (black). **c** All SNVs (*n* = 41,318,716) grouped by their pan-cancer recurrence count (1–7 +). Heatmap showing the relative contribution (color) of all mutational signatures (x-axis) to hotspot mutations of increasing recurrence (y-axis). Colors represent log_2_-fold change in mean signature posterior probability relative to singleton SNVs (recurrence 1). Several mutational signatures are enriched (red) in highly recurrent hotspots (recurrence 5, 6, 7 +). **d** Mutation rates of all mutated 11-mers (1 + ; 98.8% [2653 Mb] of the genome) and 11-mers with a hotspot in at least one of its instances for all hotspots (2 + ; 35.5% [954 Mb] of the genome), and highly recurrent hotspots (5 + ; 0.9% [23 Mb] of the genome)

Our niche and key focus is localized processes with long mutational contexts. We expect that mutational events with long consensus contexts are rare among the vast catalog of mutations. To study such rare events, we focus on the contexts associated with mutational hotspots. We consider hotspots as proxies for highly mutable positions in the genome. We hypothesize they may be targeted by highly localized and hence context specific mutational processes, which we aim to characterize. From recurrently mutated positions (Fig. 4a), we identified 2,842,934 SNVs across 1,339,497 hotspots in the non-coding part of the genome and 17,856 SNVs across 8173 hotspots in protein-coding regions (Fig. 4b) [5, 7].

Highly recurrent hotspots, where ≥ 25 genomes share the mutation, are mainly found in protein-coding regions (62% [8 out of 13]; Fig. 4b). These include drivers in known cancer genes such as *KRAS*, *BRAF*, and *TP53* [62] and they are the results of recurrent positive selection [7]. We omit protein-coding regions from our analysis (“Methods”), as hotspots in these regions are often a result of recurrent selection rather than shared localized mutational processes [5, 7].

We next asked whether any mutational signatures were enriched at hotspots, which would suggest they captured partly localized mutational processes with strong context preferences.

We quantified each signature’s mean exposure in hotspot SNVs and singleton SNVs. We compared the mean exposure across multiple recurrence levels (2, …, 7 +) to singletons (1; baseline for this analysis) and evalutated the log-fold change as the log2-ratio.

Most signature contributions are unchanged or depleted in hotspots. For example, exposure to tobacco (signature 4) explains 39% of singletons, while it only explains 11% of highly recurrent mutations (1.8-fold depletion; Fig. 4c). Such a lack of signal might occur for both technical and biological reasons: technically, localized components of mutational processes may be poorly captured by signatures if they only constitute a small fraction of a patient's total mutations [63]. Biologically, some mutational processes may simply not be localized and hence not enriched at hotspots or even depleted, if some other signature is relatively enriched at hotspots.

We found that several mutational signatures of both known and unknown etiologies were enriched among hotspots and that their enrichment often increased with recurrence (Fig. 4c). Specifically, we found that the signature 17b signal in highly recurrent (5, 6, 7 +) SNVs was 6.4-fold enriched from singletons. We also found hotspot-enriched signatures related to UV (signatures 7a, 67, 75, 7b), POLE (62, 10a), POLI (9), lymphoma-linked (72), and several signatures of unknown etiologies (17b, 17a, 19, 68, 28, 30).

The hotspot-enriched signatures are generally unique in their mutation profile. Only those with the same proposed etiology had high cosine similarities (≥ 75%; Additional file 1: Fig. S3), namely signatures associated with POLE deficiency (62 and 10a) and those associated with UV (7a and 67, 7a and 7b). When comparing enriched signatures with all other signatures (Additional file 1: Fig. S3), we found only four signature pairs with high similarities. Among these four pairs, no patients were exposed to both signatures in a pair. Thus, we do not expect the enriched signatures to overlap with other signatures within the same set of patients.

We consider hotspots to represent only a subset of the highly mutable positions and contexts in the genome, which happens to be mutated multiple times across the analyzed set of genomes. To evaluate this, using the full dataset, we compared mutation rates across nested sets of 11-mers defined by harboring mutations of increasing recurrence (Fig. 4d): 11-mers that harbor at least one (1 +) singleton mutation (*n* = 1,796,253 11-mers spanning 2,653 Mb), 11-mers with mutations in two or more (2 +) genomes (*n* = 351,996; 954 Mb), and 11-mers mutated in five or more (5 +) genomes (*n* = 3817; 23 Mb). The span of these hotspot induced 11-mer sets were much larger than their defining sets of hostpots, with an 712 × (954 Mb/1.3 Mb) increase for the 2 + set and an 3813 × (5 + ; 23 Mb/6.2 Kb) increase for the 5 + set. The mutation rate of the 2 + hotspot set (10.02 SNV/Mb/patient) was 1.7-fold increased over the 1 + singleton set (5.96 SNV/Mb/patient), while the 5 + hotspot set (25.53 SNV/Mb/patient) had an 4.3-fold increased mutation rate.

When we excluded the hotspot mutations used to identify and select the included 11-mers, the mutation rates were still elevated by 1.6-fold for the 2 + set and by 4.5-fold for the 5 + set (Additional file 1: Fig. S4). This shows that the high mutation rates of these 11-mers are not the result of an ascertainment bias and that the observed rate elevations are also driven by singleton mutations. Thus, hotspots enable us to identify highly mutable 11-mer families.

### Characterization of mutational signatures enriched at hotspots

**Fig. 5:**
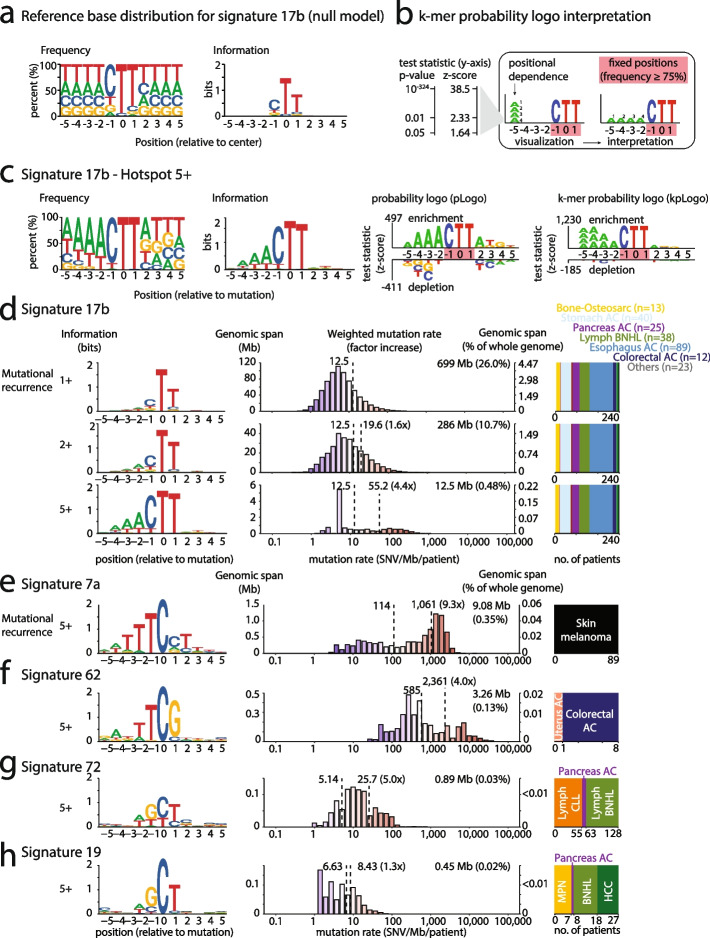
Hotspots capture highly mutated 11-mer sets. **a** Reference base distribution scaled by the mutational profile of signature 17b. The frequency logo (left) shows the percentage of each base that occupies a given position. The information logo (right) shows the Kullback–Leibler divergence (bits) of each base compared to the base distribution in the reference genome (chromosome 1–22; A = 29.5%; C = 20.5%; G = 20.5%, T = 29.5%). This signature-scaled base distribution is used as background input to the probability logo software. **b** Interpretation of positional dependencies as visualized by kpLogo. The bases of a given k-mer (*k* ≤ 4) is stacked vertically within the position it starts from with the top base (A^1^) at the start (position -5) and the bottom base (A^4^) at the end (position -2). The vertical k-mer (A^1^A^2^A^3^A^4^) should be interpreted as the most significant sequence of bases at that given position (-5). Only the most significant k-mer is shown at each position. As the logo software (pLogo and kpLogo) maxed out at *p*-value = 10^–300^ (equivalent to z-scores above 38.5), significance is reported using z-scores. **c** Example of motif visualization for signature 17b using four types of logo plots. The frequency logo and the information logo are produced as in panel a. pLogo and kpLogo quantify the surprise of observing a letter given a binomial distribution, where kpLogo only shows the most surprising k-mer (*k* ≤ 4) at each position. pLogo and kpLogo use as background the expected base distribution under a given signature, for signature 17b, the background is equivalent to the base distributions in panel **a**. **d** Signature 17b-assigned 11-mers of all recurrences-levels (1 + ; top horizontal panels), 11-mers with a hotspot in at least one of its instances (2 + ; middle horizontal panels), and 11-mers with a highly recurrent hotspot in at least one of its instances (5 + ; bottom horizontal panels). Information logo plots use as background the base distribution from the reference genome (left logo plot). Genomic span (y-axis) distribution on mutation rates (x-axis; middle histogram). Cancer type distribution within the cohort (right stacked bar plot), colored as in Fig. 1a. **e** UV-signature 7a-assigned 11-mers with a highly recurrent hotspot in at least one of its instances (5 +). Plots are interpreted as in panel **d**. **f** POLE-signature 62-assigned 11-mers with a highly recurrent hotspot in at least one of its instances (5 +). Plots are interpreted as in panel **d**. **g** Signature 72-assigned 11-mers with a highly recurrent hotspot in at least one of its instances (5 +). Plots are interpreted as in panel **d**. **h** Signature 19-assigned 11-mers with a highly recurrent hotspot in at least one of its instances (5 +). Plots are interpreted as in panel **d**

To identify and statistically evaluate nucleotide contexts characteristic of highly mutable 11-mers, we applied four different motif visualization methods showing: (1) the relative base frequency (Fig. 5a), (2) the information content (Fig. 5a), (3) base frequency significance, using pLogo [57], and (4) k-mer frequency significance, using kpLogo [58] (Fig. 5b). For the significance evaluations (methods 3 and 4), we generated a background distribution of nucleotide patterns matching what would be expected from the signature in question (Fig. 5a and Additional file 1: Fig. S5; “Methods”).

We recurrence-stratified signature-assigned 11-mer sets. For signature 17b-assigned 11-mers with high recurrence levels (5 +), we found a strong enrichment of adenines in the three upstream positions (fourth, third, and second 5’-neighbor) from the mutated base supported by all four visualization methods (AAACTT; Fig. 5c and Additional file 1: Fig. S2). The kpLogo even showed a 29-fold enrichment (*p* = 10^–296^; kpLogo test) of four consecutive 5’ adenines (AAAA) neighboring the CTT core trinucleotide (AAAACTT). This subset of 11-mers spanned 12.5 Mb (0.48% of the genome) with a mean mutation rate 4.4-fold higher (55.2 SNV/Mb/patient) than the overall signature 17b-assigned 11-mer rate (12.5 SNV/Mb/patient *p* < 10^–318^; binomial test). A subset (AACTT) of this motif has also been reported by Stobbe et al. (2019) [21], while Alexandrov et al. (2020) [22] showed high mutation probabilities in ACTTA contexts when fitting pentanucleotide signatures. The wide range of cancer types affected by this signature includes mainly adenocarcinomas of the digestive system (esophagus, stomach, colorectum, pancreas; *n* = 166), but also B-cell non-Hodgkin lymphoma (BNHL; *n* = 38), osteosarcoma (*n* = 13), and others (*n* = 23; Fig. 5d).

We found that the UV-associated signature 7a was enriched in hotspots (Fig. 4c), and the mutation rate of signature 7a-assigned 11-mers with 5 + hotspots (114 SNV/Mb/patient; 9 Mb) was enriched 9.3-fold compared to all signature 7a-assigned 11-mers (60 SNV/Mb/patient; 1 Gb; *p* < 10^–318^; binomial test; Fig. 5e). The nucleotide composition of this subset displayed trends towards the TCS (S = C|G) center trinucleotide flanked by additional up- and downstream thymines (TTTCST; Additional file 1: Fig. S2). This motif has previously been reported [21, 29, 30]. While the emergence of this motif is driven by highly mutated 11-mers with mutation rates above the mean (114 SNV/Mb/patient), we observed a different nucleotide composition in the lowly mutated contexts (WSYT; W = A|T, Y = C|T; Additional file 1: Fig. S6).

In genomes from adenocarcinoma of the colorectum (*n* = 7) and uterus (*n* = 1), 11-mers with 5 + hotspots assigned to the mutational signature 62 of POLE deficiency displays mutation rates 4-fold higher (2,361 SNV/Mb/patient; 3 Mb) than all signature 62-assigned 11-mers (585 SNV/Mb/patient; 208 Mb; *p* < 10^–318^; binomial test; Fig. 5f and Additional file 1: Fig. S2). We found this set of 11-mers to be characterized by the TCG center trinucleotide flanked by upstream AGT (11-fold enrichment; *p* = 10^–296^; kpLogo test) and downstream AGAC (24-fold enrichment; *p* = 10^–296^; kpLogo test) to establish a combined 10-bp motif (AGTTCGAGAC). From a pentanucleotide signature model, Alexandrov et al. (2020) [22] showed that signature 62 has moderate preference towards C > T substitutions in a TTCG context; however, they found that C > A substitutions in TTCTT were much more likely for this signature. The TTCG context has also been reported by others [22, 64, 65]. Our findings suggest that POLE-associated signature 62 displays specifically highly localized mutagenesis in AGTTCGAGAC contexts, adding several nucleotides to the known POLE-motif (TTCG).

We also found highly increased mutation rates and strong sequence specificities towards the TTCTTT 6-bp motif for POLE-signatures 10a (2.1-fold; 1582 SNV/Mb/patient; 3 Mb; *p* < 10^–318^; binomial test) and 61 (1.6-fold; 1098 SNV/Mb/patient; 15 Mb; binomial test; Additional file 1: Fig. S2). This motif is one position wider than the pentanucleotide motif, TTCTT, modeled by the POLE-associated pentanucleotide signatures 10a, 61, 62, 63, and 66 from Alexandrov et al. (2020) [22].

For signature 72, associated with B-cell lymphomas (BNHL and chronic lymphocytic leukemia), we observed 5-fold increased mutation rates (26 SNV/Mb/patient; 0.9 Mb; *p* < 10^–318^; binomial test) in the 5 + set over all the signature 72-assigned 11-mers (5 SNV/Mb/patient; 185 Mb; Fig. 5g). The nucleotide context showed a strong trend toward the AGCT motif; however, this trend was not confirmed by kpLogo. Though signature 72 has no clear etiology, this motif is identical to the hotspot motif of AID activity [66, 67], known to be involved in lymphomagenesis [68].

The AID hotspot motif also emerged from the 5 + set assigned to signature 19; however, the mutation rates increase of 1.3-fold was not significant (8 SNV/Mb/patient; 0.5 Kb; *p* = 0.5) compared with all signature 19-assigned 11-mers (7 SNV/Mb/patient; 111 Mb; Fig. 5h). Like signature 72, signature 19 is active in BNHL genomes (*n* = 10) and pancreatic adenocarcima (*n* = 1), but also myeloproliferative neoplasm (*n* = 7) and hepatocelluar carcinoma (*n* = 9). No etiology has been proposed for this signature. Though the mutational profile of signature 19 is very different from signature 72 (cosine similarity = 0.24), the signature 72- and 19-assigned 11-mers with hotspots share sequence contexts supporting a relatedness to AID-mutagenesis.

### Localized mutational processes are operative in distinct genomic elements

**Fig. 6:**
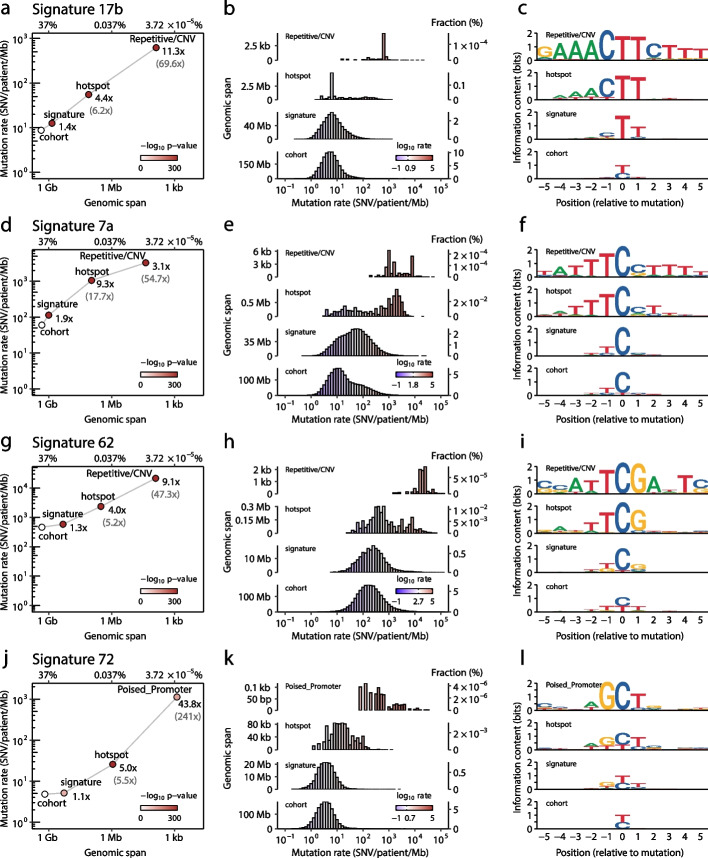
Genomic subsets with highly elevated mutation rates. **a** The decreasing genomic spans (x-axis) and increasing mutation rates (y-axis) are shown for nested genomic subsets for the signature 17b cohort. The cohort mutation rate is based on the entire non-coding genome, followed by the signature assigned 11-mers, hotspot-associated 11-mers, and finally, the subset falling in the genomic region with the highest (significant) observed mutation rate. The relative mutation rate increase from the prior set is shown and its significance indicated (red color scale; Bonferroni corrected *p*-value based on all 817 tests in full study; see Additional file 1: Fig. S2 for specific values). The overall total rate change compared with the cohort is given parenthetically. Mutation rate confidence intervals (CI-99%) are narrow and therefore invisible. **b** The genomic spans (y-axis) of genomic positions binned by their mutation rates (x-axis; log-scale) for the cohort, signature, hotspot, and genomic region subsets as defined above. The level of a mutation rate increase (red) or decrease (blue) is shown relative to the mean cohort mutation rate (8.72 SNV/Mb/patient for signature 17b; white). **c** Sequence information content surrounding the SNVs for each of the four genomic subsets defined in **a**. **d, e, f** UV-induced signature 7a genomic subsets visualized as in panels **a–c**. **g, h, i** POLE (polymerase epsilon deficiency) signature 62 genomic subsets visualized as in panels **a–c**. **j, k, l** Signature 72 (lymphoma-linked; unknown etiology) genomic subsets visualized as in panels **a–c**. Coresponding results for all signatures are given in Additional file 1: Fig. S1

To evaluate whether the hotspot-associated mutational processes show preference for specific genomic regions, we examined the mutation rate of signature-assigned 11-mers within functional genomic elements from ENCODE [69] and compared them to the equivalent subsets of genome-wide 11-mers. In multiple cases, we found significant regional differences (Fig. 6 and Additional file 1: Fig. S2).

For signature 17b, the mutation rate of 11-mers increased in insulators (2.1-fold; 46 Kb; *p* < 10^–125^; binomial test), heterochromatin (1.3-fold; 8.4 Mb; *p* < 10^–318^; binomial test), and repetitive regions (11-fold; 7.6 Kb; *p* < 10^–318^; binomial test) (Additional file 1: Fig. S2). While there was no clear signal of 5’-A-tracts in insulators and heterochromatin (pLogo and kpLogo; Additional file 1: Fig. S2), the repetitive regions displayed strong enrichment for an extended motif (GAAACTTCTTT; Additional file 1: Fig. S2) beyond what is captured by hotspots (AAACTT; Fig. 5c). Interestingly, this 11-bp sequence context also showed high mutation rates for POLE signature 63 in repetitive regions (6676 SNV/Mb/patient; 6.4-fold; 0.7 Kb; *p* < 10^–7^; binomial test) (Additional file 1: Fig. S2).

To further evaluate GAAACTTCTTT mutability in repetive elements, we annotated 11-mer instances with repeat-classes from RepeatMasker [56] (“Methods”; Additional file 1: Fig. S7). We found that this 11-mer is indeed highly mutable (434 SNV/Mb/patient) in repetitive regions pan-cancer. Additionally, we observed that the mutated instances almost exclusively (82.8%; 1200 out of 1450) occured in alpha satellite repeats (670 SNV/Mb/patient), characteristic of the centromeres.

For the UV signature 7a, 11-mer mutation rates increased in heterochromatin (1.2-fold; 6 Mb; *p* < 10^–318^; binomial test), enhancers (1.2-fold; 11 Kb; *p* < 10^–12^; binomial test), promoters (2.2-fold; 6 Kb; *p* < 10^–140^; binomial test), and repetitive regions (3.1-fold; 23 Kb; *p* < 10^–318^; binomial test) (Additional file 1: Fig. S2). The 11-mer subsets within promoter, heterochromatin, repetitive, and enhancer regions had strong sequence tendencies towards the TTTCSTT (S = C|G) motif, consistent with previous reports of T-tracts in UV hotspot motifs [26, 70, 71]. In addition, we found highly enriched downstream motifs in active promoters (159-fold; from position + 2; TCCG; *p* = 10^–296^; kpLogo test) and repetitive elements (368-fold; from position + 1; GATTC; *p* = 10^–296^; kpLogo test). Interestingly, promoters have previously been coupled to increased UV-mutability [27, 29].

The POLE-associated (signature 62) subsets displayed strong sequence preferences for the POLE-motif (TTCG) and dramatically increased mutation rates in poised promoters (4.5-fold; 2 Kb; *p* < 10^–58^; binomial test), enhancers (3.5-fold; 2 Kb; *p* < 10^–34^; binomial test), and repetitive elements (9.1-fold; 8 Kb; *p* < 10^–318^; binomial test) (Additional file 1: Fig. S2). Additionally, we recovered the hotspot sequence motif in insulators (upstream AGT, fivefold, *p* = 10^–308^; downstream AGAC, 18-fold, *p* = 10^–296^; kpLogo test) and parts of the motif in strong enhancers (AGT; ninefold), weak enhancers (AGAC; 12-fold), transcribed elongation (AGAC; 25-fold), transcribed transition (AGAC; 27-fold), and repressed regions (AGAC; 21-fold) all at the same significance levels (*p* = 10^–296^; kpLogo test). The recovery of parts of the hotspot motif (AGTTCGAGAC) in multiple genomic regions supports a localized behavior associated with POLE-deficiency.

We found increased mutation rates for the B-cell lymphoma signature 72 in active promoters (21.6-fold; 0.2 Kb; *p* < 10^–10^; binomial test), weak promoter (12.1-fold;; 0.8 Kb; *p* < 10^–20^; binomial test), and poised promoters (43.8-fold; 0.8 Kb; *p* < 10^–142^; binomial test), all of which were enriched for motifs compatible with the AID-motif (AGCT; Additional file 1: Fig. S2). Similarly, signature 19 subsets had dramatically increased mutation rates and AID-compatible motifs in poised promoters (WGCT; 143-fold; 0.1 Kb; *p* < 10^–11^; binomial test) and weak promoters (AGCT; 86-fold; 0.1 Kb, *p* < 10^–2^; binomial test; Additional file 1: Fig. S2).

### Several signatures exhibit strongly localized behavior

In combination, we identified sets of positions in specific genomic regions that are targeted by localized mutational processes and subject to much elevated mutation rates (Fig. 6). We can decompose the increase in mutation rate into explanatory factors. Together, these factors each define increasingly smaller parts of the genome where the underlying processes are increasingly active. This allows us to identify the sequence characteristics of highly mutable contexts and the relative rate increase they contribute.

For instance, for signature 17b (Fig. 6a–c), the exposure-cohort has a mutation rate (8.72 SNV/Mb/patient; 2300 Mb) slightly higher (1.5 ×) than the pan-cancer baseline (5.96 SNV/Mb/patient; 2653 Mb). The subset of 11-mers likely targeted by signature 17b showed an increased (1.4 × over the cohort rate) mutation rate (12.5 SNV/Mb/patient; 699 Mb) with a remarkable nucleotide bias for a 5’-A-tract (Additional file 1: Fig. S2). Recurrently mutated contexts (6.2 × ; 12.5 Mb; AAACTT) and repetitive regions (69.6 × ; 7.6 Kb; GAAACTTCTTT) further restrict the set of positions to well-defined contexts (Fig. 6c; Additional file 1: Fig. S2) with high mutation rate (434 SNV/Mb/patient). This mutational signature has been associated with gastrointestinal cancers and exposure to the genotoxic chemotherapy 5-fluorouracil, though no explanation exists for increased mutability in this highly defined nucleotide sequence [72]. Where available (136 out of 240 patients), the clinical data showed that no patients were exposed to neoadjuvant chemotherapy, thus these tumors are treatment naive and we can rule out 5-fluorouracil as the explanatory process for them.

Samples exposed to the main UV-signature (7a) generally have high mutation rates (60 SNV/Mb/patient; 2129 Mb). When restricted to contexts likely targeted by signature 7a (1.9 × ; 1002 Mb), contexts with mutational recurrence (17.7 × ; 9.1 Mb), and finally repetitive regions (54.7 × ; 23.8 Kb), the mutation rate increases at scales similar to signature 17b (Fig. 6d–f). Despite their differences in exposed tissues, the processes underlying signatures 7a (UV) and 17b (unknown) both prefer sequence motifs with A/T-tracts 5’-adjacent to the mutated nucleotide at similar rates.

Generally, patients exposed to POLE-signature 62 had very high mutation rates (463 SNV/Mb/patient; 2121 Mb) with high fractions (median exposure 17.9%) of mutations explained by this signature (Fig. 6g–i). There was only a modest increase in mutation rate (1.3 × ; 206 Mb) when focusing on likely signature 62 target contexts. When further narrowing the subset, both high mutational recurrence (5.2 × ; 3.3 Mb) and location in repetitive regions (47.3 × ; 8.0 Kb) contributed large mutation rate increases. When comparing rate increases across multiple POLE signatures, mutational recurrence in POLE-assigned 11-mers contributed slightly less to the mutation rate for signatures 10a (4.6 × ; 3 Mb) and 61 (2.4 × ; 15 Mb) compared to signature 62 (5.2 × ; 3.3 Mb). In addition, the preferred core motif is different from signature 10a and 61 (TTCT) to signature 62 (TTCG) (Additional file 1: Fig. S1 and S2). This may reflect distinct mechanistic processes of POLE deficiency.

The signature 72-exposed cohort generally had a low mutation rate (4.81 SNV/Mb/patient; 1533 Mb) and focusing on the likely target context contributed only a slight mutation rate increase (1.1 × ; 185 Mb). However, mutational recurrence captured a nucleotide pattern (AGCT) known as the AID-hotspot motif [66, 67], an increased rate (5.5x; 0.9 Mb) similar to the effect seen for signatures 17b (6.2x), 7a (17.7x), and 62 (5.2x) (Fig. 6j–l).

In the four cases above (Fig. 6), hotspots associated 11-mers contributed a 5–18 × increase of mutation rate over the cohorts and 4–9 × increase of mutation rate over the mutational signature cohorts. The latter being consistent with our signature-agnostic hotspot-characterization (4.3 × ; Fig. 4d). The analysis of mutational hotspots and their associated 11-mers have facilitated a characterization of the mutation rates and sequence contexts of localized mutational processes.

## Discussion

In this study, we exploited mutational hotspots to define subsets of the genome that are targeted by localized mutational processes and systematically catalog their mutation rates and sequence preferences. We found that mutation rates of contexts subject to localized mutational processes (UV-signature 7a, POLE-signature 62, lymphoma-signature 72, and unknown etiology-signature 17b) were 4–9-fold increased compared to what can be explained by cancer type and mutational signature alone. Additionally, we found that mutation rates are further elevated in sequence motifs within genomic regions related to repetitive DNA (3–11-fold; signatures 17b, 7a, 62) and promoters (44-fold; signature 72) (Fig. 6). We provide a comprehensive catalog of localized mutational processes, their sequence motifs, and their observed mutation rates (Additional file 1: Fig. S1 and S2).

In our analysis of signature-assigned 11-mers, we found that signatures associated with endogenous mutational processes, such as age, MMR, POLE, and APOBEC, generally spanned low fractions of the genome (2–8%), while the genomic spans of 11-mers assigned to signatures associated with exogenous mutational processes, such as tobacco (42%), UV (37%), and signature 17b (26%; likely exogenous cause [73, 74]), were much larger (Fig. 3g). It is tempting to speculate that the large genomic span differences may result from endogenous mutational processes depleting their target contexts from the germline genome, resulting in lower steady-state abundances over evolutionary time. Contrarily, it is less likely that exogenous mutational processes prevalent in somatic evolution deplete target contexts in the germline genome, resulting in higher steady-state abundances, supporting the genomic span differences of targets of endogenous and exogenous mutational processes.

Mutational signature analysis has become a well-established statistical inference method for studying mutational processes. We use the approach to assign individual mutations to the signature that best explain its occurence. As for all statistical methods, there is a risk of misclassification. This risk will be especially prevalent for mutational signatures with overlapping mutation type profiles. For the hotspot-enriched signatures, we found little risk of misclassification, as they had low similarities.

Consistent with literature, we found that UV-associated mutagenesis (signature 7a) targets TTTCST-sequences (S = C|G), which are highly mutated across multiple genomic regions [21, 26, 30]. In addition to this highly mutated context, we observed a neighboring TCCG motif in promoter regions suggesting a combined TTTCSTCCG motif. Interestingly, melanoma genomes frequently harbor hotspot mutations in promoter elements explained by ETS-mediated sensitization of DNA to UV-induced cyclobutane pyrimidine dimer formation [27, 28, 75, 76]. The binding of DNA by ETS-transcription factors is estimated to contribute a 16–170-fold elevated mutation rate at ETS-binding sites (CTTCCGG and YYTTCC) [28, 76]. We did not observe this ETS motif in our analyses. For UV-assigned 11-mers with high recurrence, we found a bimodal distribution of mutation rates associated with different sequence preferences (TTTCST [high] and WSYT [low]), thus potentially capturing multiple mechanisms by which UV may induce mutations. This shows that our k-mer-centric and rate-based analysis approach can aid in the generation of mechanistic hypotheses for mutational processes. Similar approaches will gain increased power in future large whole-genome cancer datasets.

We observed that two signatures of unknown etiology (signatures 19 and 72) are associated with a hotspot motif (WGCT), which is compatible with the known AID hotspot motif (AGCT) [66, 67]. Additionally, these processes have increased mutability in promoters, which is in line with reported AID off-target effects [77]. Thus, the potential of capturing AID mutagenesis through signatures 19 and 72 may be further explored.

We found that the rate of signature 17b-mutations is elevated (9-fold) in a genome-wide hotspot motif (AAACTT) (Fig. 5c–d), which adds more context to the previously identified signature 17-motifs (ACTTA and AACTT) [21, 22, 73].

Consistent with signature 17 mutations being enriched in cohesin/CTCF-binding sites [78–80], we found a 2-fold mutation rate increase in certain contexts within insulator elements (Additional file 1: Fig. S2). However, in these regions, we did not observe the signature 17b-characteristic 5’-A-tract before the CTT core nucleotides. Thus, the mutational mechanism acting in these elements may be distinct from those causing AAACTT-hotspot mutations in the rest of the genome.

Unexpectedly, we also found a highly enriched 11-mer (GAAACTTCTTT) in the alpha satellite repeats of centromeric regions (Additional file 1: Fig. S7), which was associated with both signature 17b and the POLE-deficiency signature 63 (Additional file 1: Fig. S2). This 11-mer contains the reported 5’-A-tract; however, it also contains some intrinsic repeat structure that may be broken down into triplicates of the repeat unit, S(W)_2–3_ (S = C|G; W = A|T). Such repeats may adopt secondary DNA structures that facilitate mutagenesis by certain processes, similar to APOBEC targeting single-stranded DNA in stem-loops [36, 38, 40] or MMR deficiency leading to increased mutability of AT-rich short inverted repeats [39]. As alpha satellite repeats are replicated in the late S-phase [81], the mutational processes shaping this part of the genome are likely linked to late replication. Mutagenesis from POLE deficiency and the signature 17 process are both associated with late replication [36, 52]. Taken together, this is consistent with GAAACTTCTTT being associated with these processes in our analyses.

Just like the other motifs subject to tissue-specific localized mutational processes, the AAACTT motif possesses properties that either increase susceptibility to DNA damage, avoidance of repair, or both. Replication-timing and strand-asymmetry profiles of signature 17 mutations have been shown to be similar to those found for signatures of tobacco and UV exposure. Thus, they may share the property of being linked to environmental DNA damage mechanisms [52]. Specifically, oxidative damage to the dGTP pool has been proposed as a possible explanation for signature 17 mutations, resulting from exposure to gastric acid in gastrointestinal tumors or exposure to the genotoxic chemotherapeutic 5-fluorouracil in treated tumors [19, 52, 73, 74]. However, these hypotheses do not explain the characteristic motif of signature 17 mutagenesis and the mechanisms involved remain largely unexplained [72].

The signature 17 mutational process has been shown to correlate with the helical periodicity of DNA wound around the nucleosome core [82]. The highest mutation rates are found in the nucleosome-facing minor grooves, likely explained by hindered base excision repair in these sites [82]. While the rigid structure of long A-tracts may constrain DNA winding around the nucleosome [83], short A-tracts likely affect nucleosomal DNA flexibility and thus direct their positioning within the nucleosome with respect to the dyad [84, 85]. Such intra-nucleosomal forces may in turn hinder DNA repair at nucleosome-facing minor groove CTT lesions, thus in part explaining the A-tract motif associated with these mutations. At least, it is possible that lesions in proximity of A-tracts are repaired at different rates than the rest of the genome [86].

In agreement with existing literature [21, 22, 64, 65], we found POLE deficiency mutagenesis to be associated with two highly mutated motifs (TTTCTTT [signature 10a & 61] and AGTTCGAGAC [signature 62]) and that their mutation rates increased over the signature-explained rates (2-4-fold). Mutations localized to the TTCG motif seem to be more pronounced for signature 62 than any other POLE signature, suggesting multiple mutagenic mechanisms of POLE deficiency. Fang et al. (2020) [65] suggest that mutations acquired in distinct domains of the POLE gene may give rise to distinct mutational patterns depending on the mutant POLE DNA affinity. Thus, it is possible that there exists even more examples of single mutagenic mechanisms generating different mutation types dependent on their specific loss- or gain-of-function mutants.

## Conclusions

Our findings provide higher resolution of the sequences targeted by localized mutational processes and contribute mutation rate estimates of these. Our comprehensive catalogs (Additional file 1: Fig. S1 and S2) of mutational processes may aid the construction of more accurate models of the mutational processes in cancer, which capture the mutation rate variation. Such models are important for accurate statistical driver identification among the landscape of passenger hotspot mutations caused by localized processes [87]. In addition, the models may also contribute to deeper understanding of cancer risk, somatic evolution, cancer development, and tumor biology.

The mutational patterns of localized processes active across cancers may serve as future biomarkers for detection of such processes and their associated etiologies in cancer samples. In samples with weak mutation signals, catalogs of localized mutational processes may power detection of active processes through targeted sequencing of their possible genomic targets. For cancer-associated mutational processes, this may translate to new opportunities for liquid biopsies to enable early cancer detection and surveillance of cancer evolution in the patient.

### Supplementary Information

**Additional file 1: Fig. S1**. Catalog of localized mutational processes and their mutation rates. **Fig. S2.** Catalog of sequence dependencies. **Fig. S3.** Cosine similarities between signatures. **Fig. S4.** Mutation rates of 11-mers with and without hotspots. **Fig. S5.** Background 11-mer sets derived from mutational signatures. **Fig. S6.** UV-signature sequence characteristics across mutation rates. **Fig. S7.** Characterization of GAAACTTCTTT-sequences in repetitive elements. **Table S1.** K-mer statistics.

## Data Availability

This study is based on the somatic mutations from the Pan-Cancer Analysis of Whole Genomes (PCAWG) consortium [6]. The data can be accessed through gbGaP (TCGA: phs000178.v11.p8) and ICGC DACO (ICGC: EGAS00001001692), with procedures described on this site: https://docs.icgc.org/pcawg/data/. Core scripts used in the analysis are available at GitHub https://github.com/JakobSkouPedersenLab/localized_mutation_rates_analysis.git [88].
