# Supplementary material for: Sequence dependencies and mutation rates of localized mutational processes in cancer
Source: Genome Med. 2023 Aug 17;15:63. doi: 10.1186/s13073-023-01217-z (PMC10436389; doi:10.1186/s13073-023-01217-z)
Supplement: Supplementary file 1 — Additional file 1: Fig. S1. Catalog of localized mutational processes and their mutation rates. Fig. S2. Catalog of sequence dependencies. Fig. S3. Cosine similarities between signatures. Fig. S4. Mutation rates of 11-mers with and without hotspots. Fig. S5. Background 11-mer sets derived from mutational signatures. Fig. S6. UV-signature sequence characteristics across mutation rates. Fig. S7. Characterization of GAAACTTCTTT-sequences in repetitive elements. Table S1. K-mer statistics. [file 13073_2023_1217_MOESM1_ESM.pdf]

Figure S1

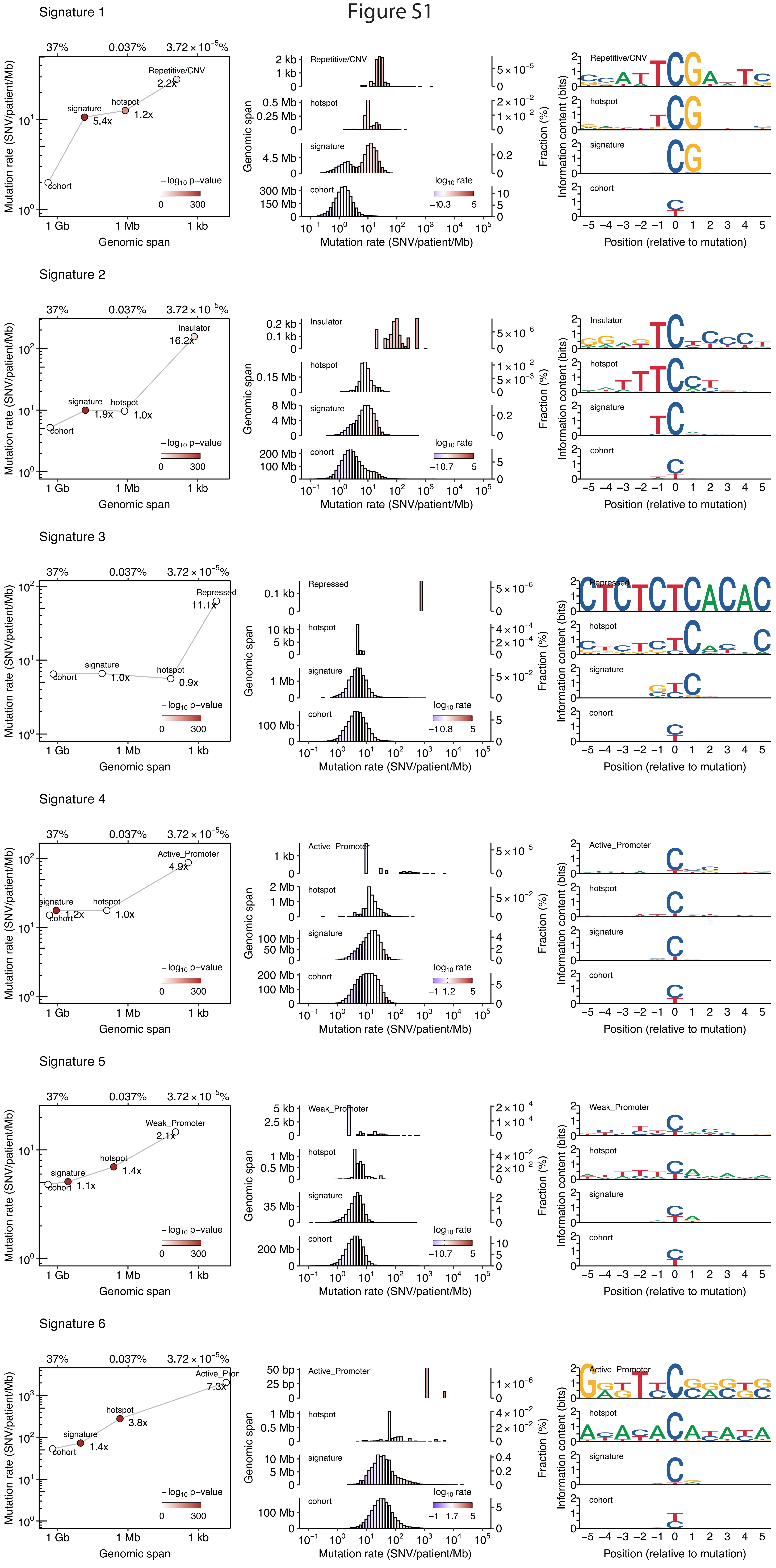
Signature 1
Signature 2
Signature 3
Signature 4
Signature 5
Signature 6
37%
0.037%
3.72 × 10^-5 %
Mutation rate (SNV/patient/Mb)
Genomic span
1 Gb
1 Mb
1 kb
−log10 p-value
0
300
cohort
signature
hotspot
Repetitive/CNV
Insulator
Repressed
Active_Promoter
Weak_Promoter
2.2x
5.4x
1.2x
16.2x
1.9x
1.0x
11.1x
0.9x
4.9x
1.2x
2.1x
1.4x
1.1x
7.3x
3.8x
Genomic span
Fraction (%)
Mutation rate (SNV/patient/Mb)
log10 rate
−10.3
−10.7
−10.8
−1 1.2
−1 1.7
5
Information content (bits)
Position (relative to mutation)
−5 −4 −3 −2 −1 0 1 2 3 4 5

Signature 7a

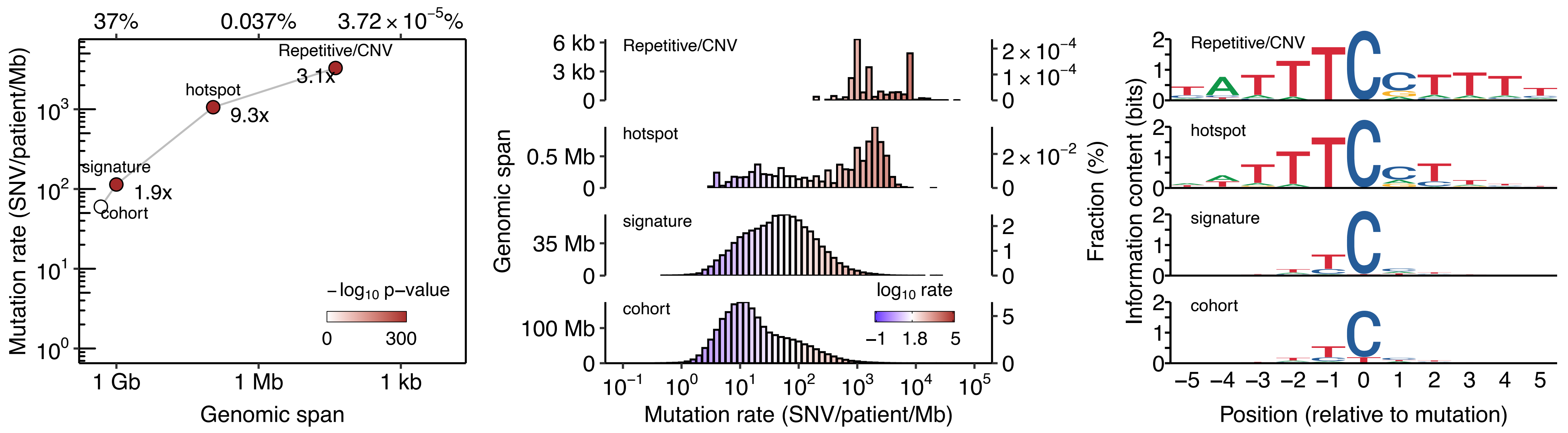

Signature 7b

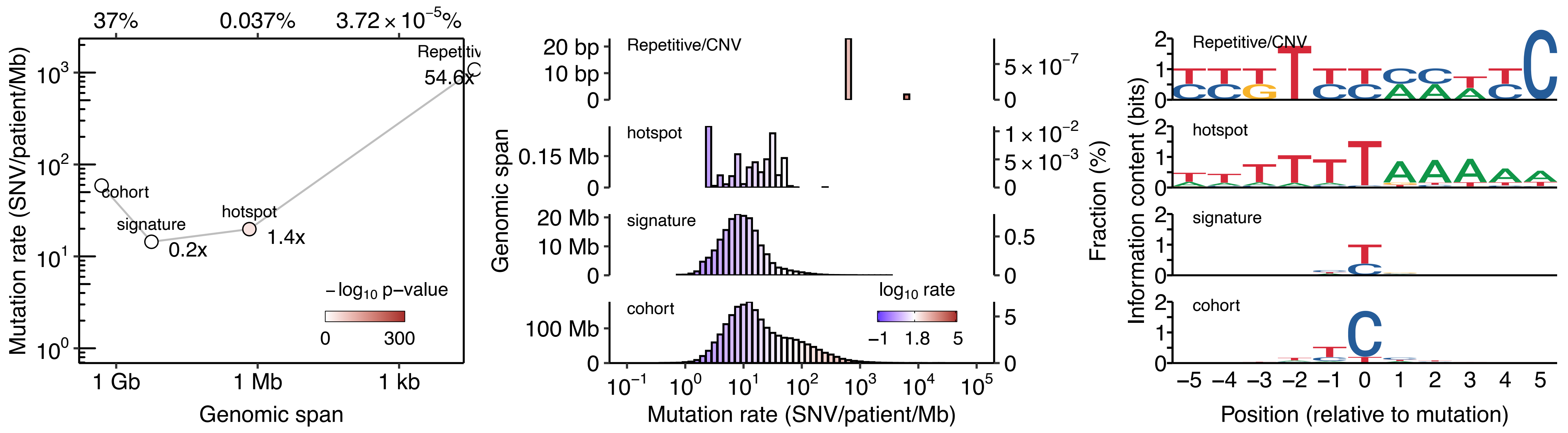

Signature 7c

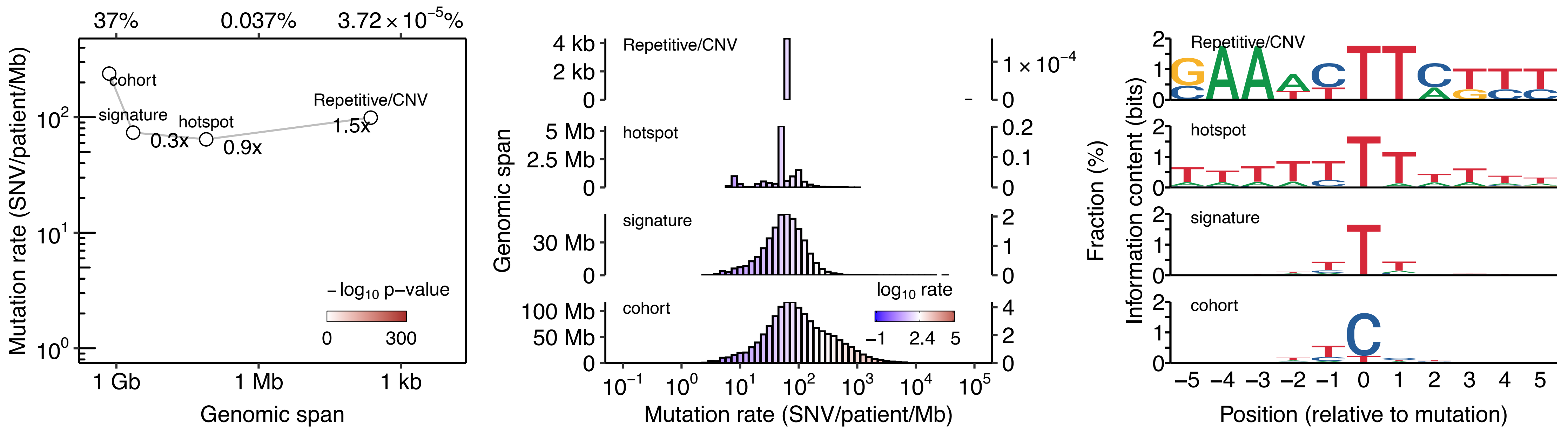

Signature 8

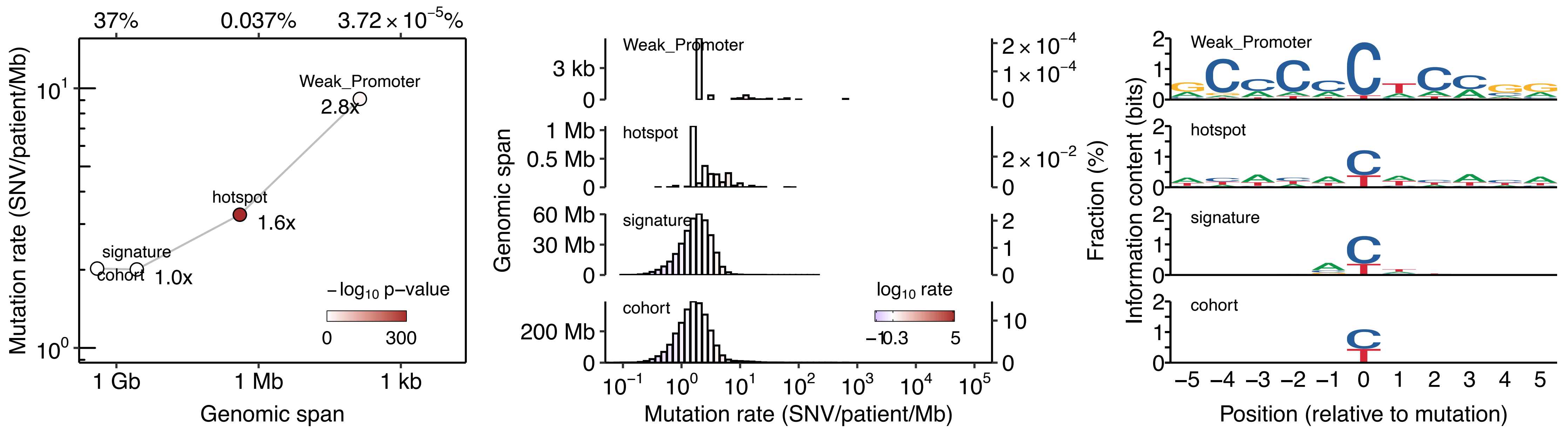

Signature 9

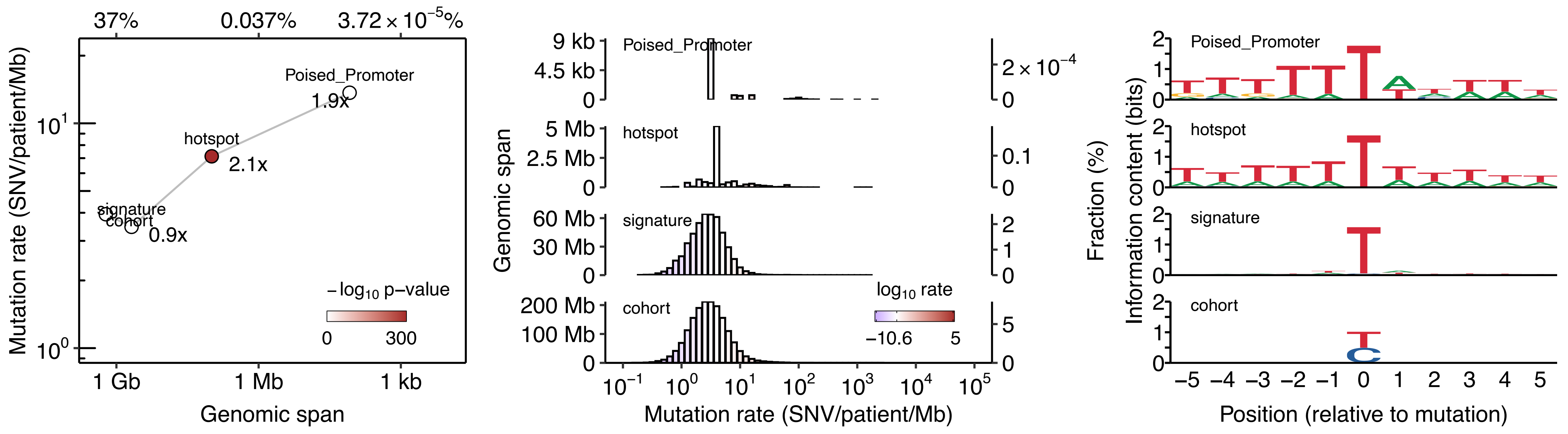

Signature 10a

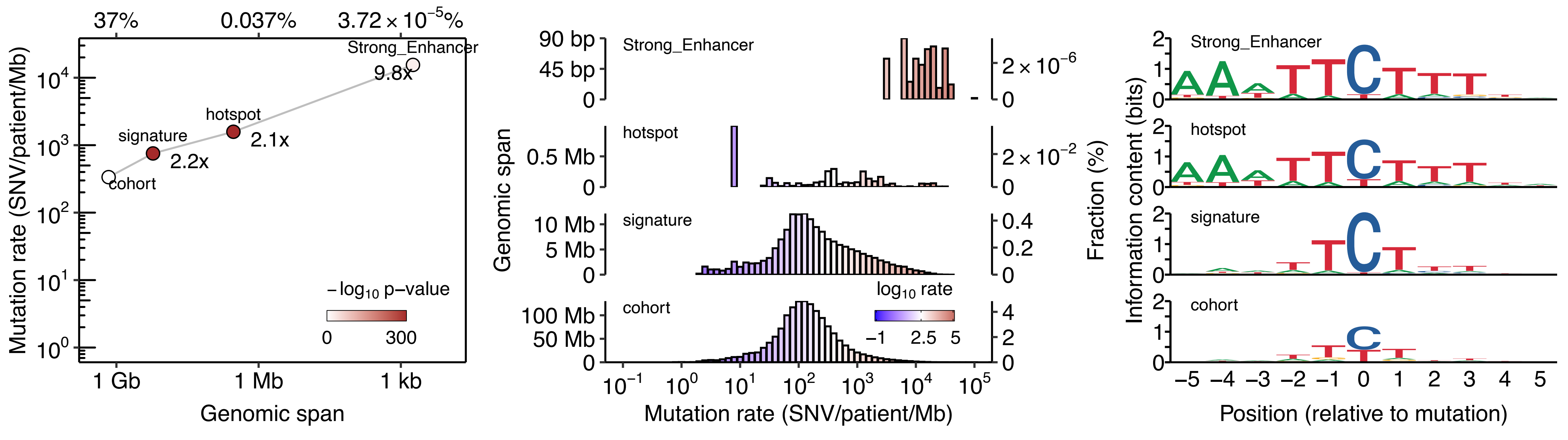

Signature 11

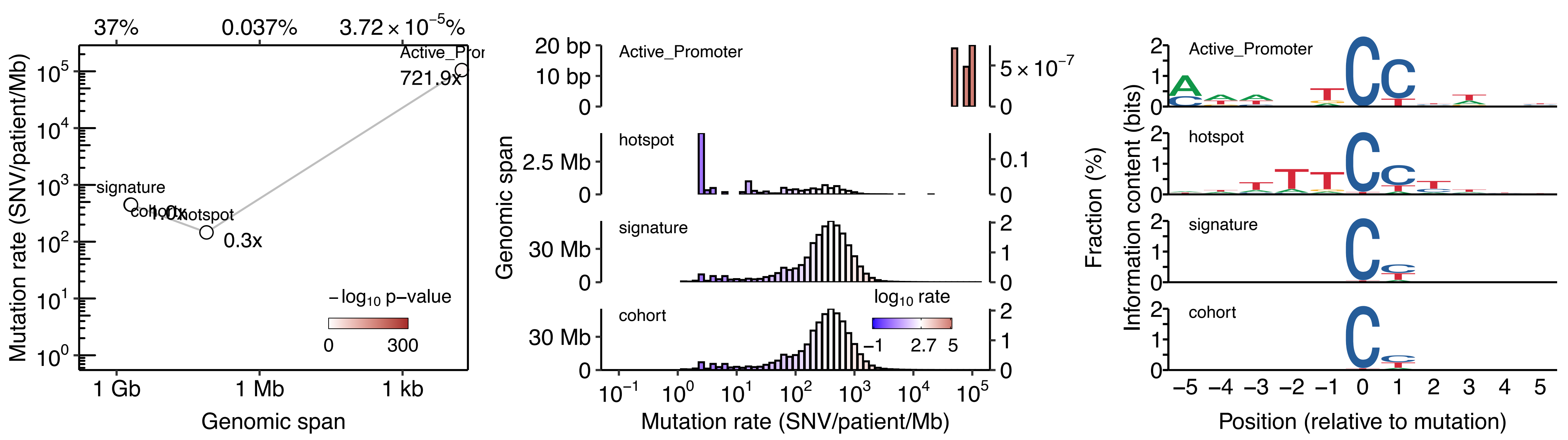

Signature 12

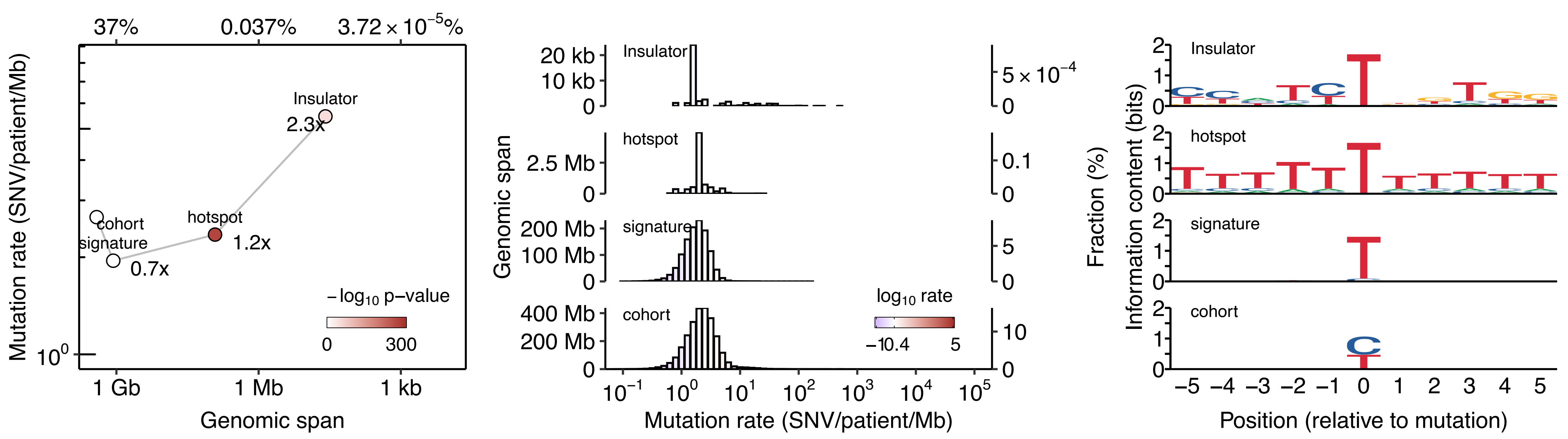

Signature 13

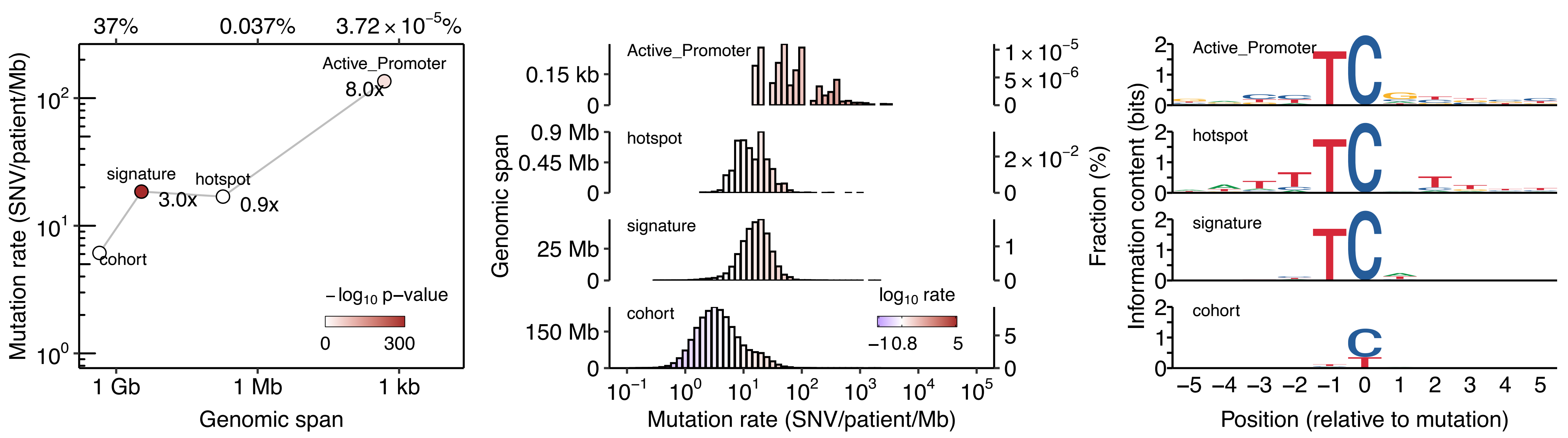

Signature 14

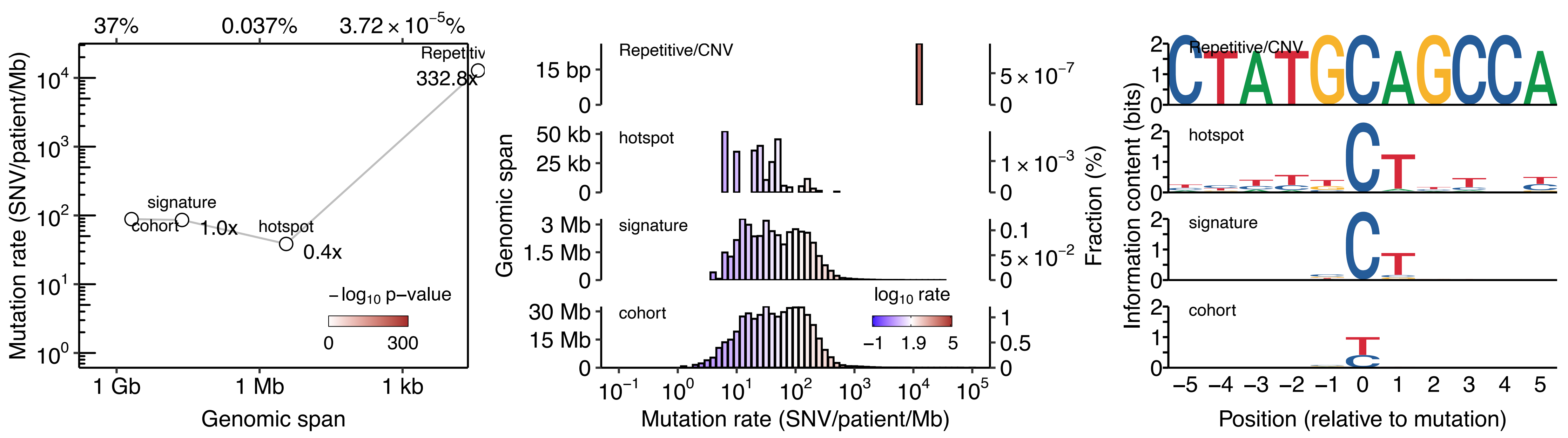

Signature 15

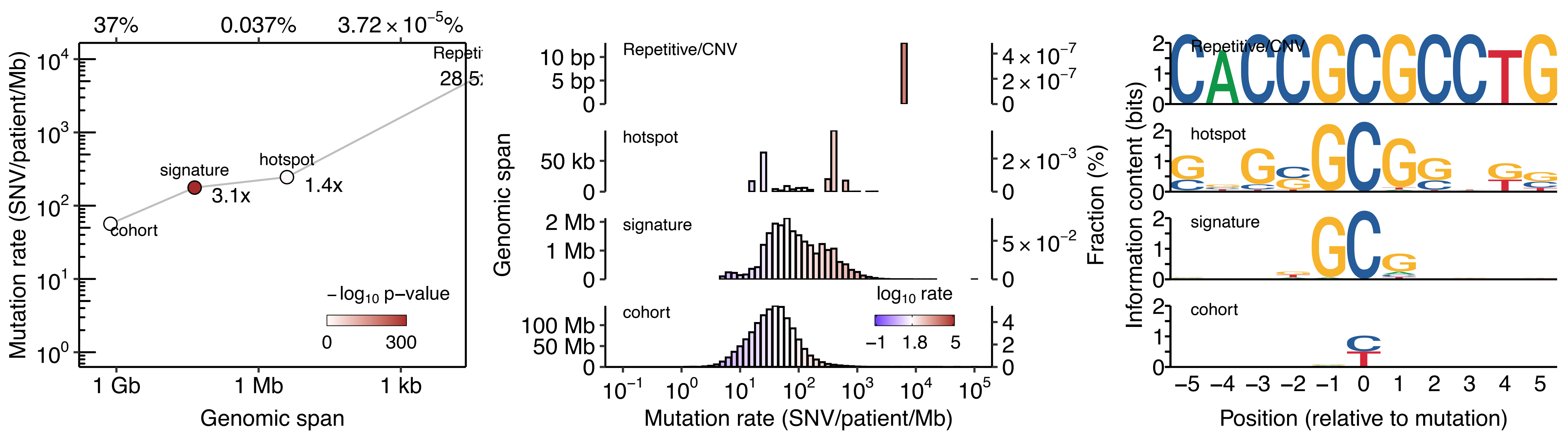

Signature 16

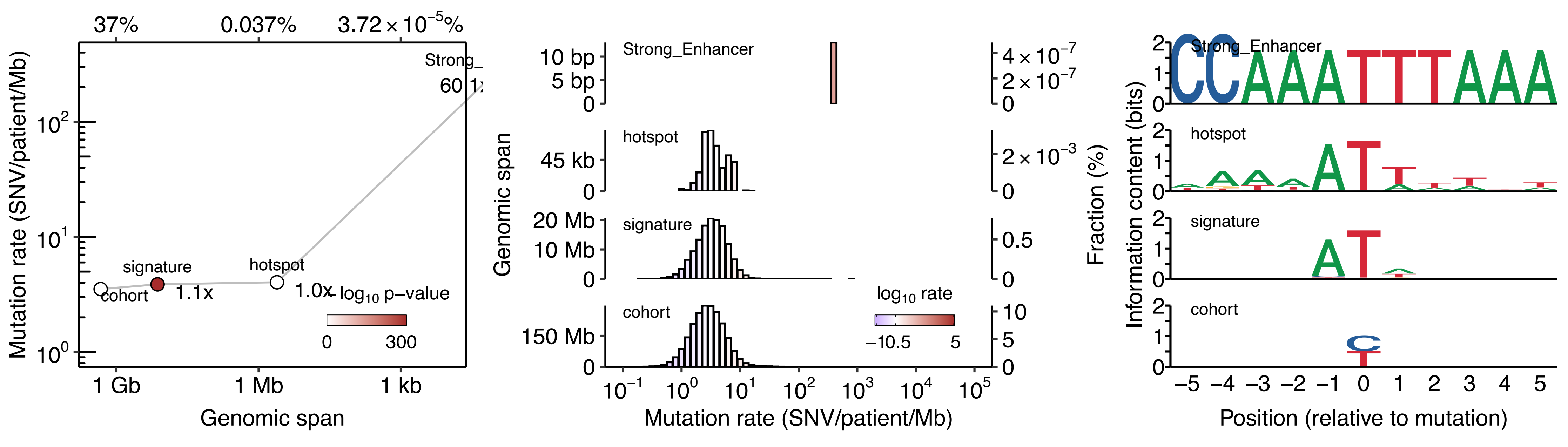

Signature 17a

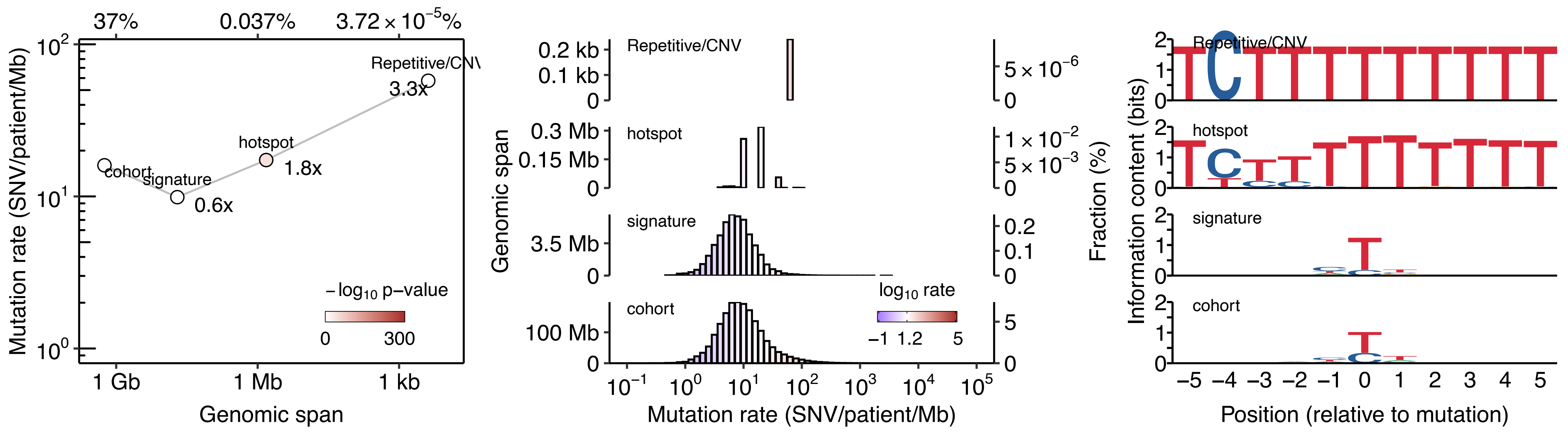

Signature 17b

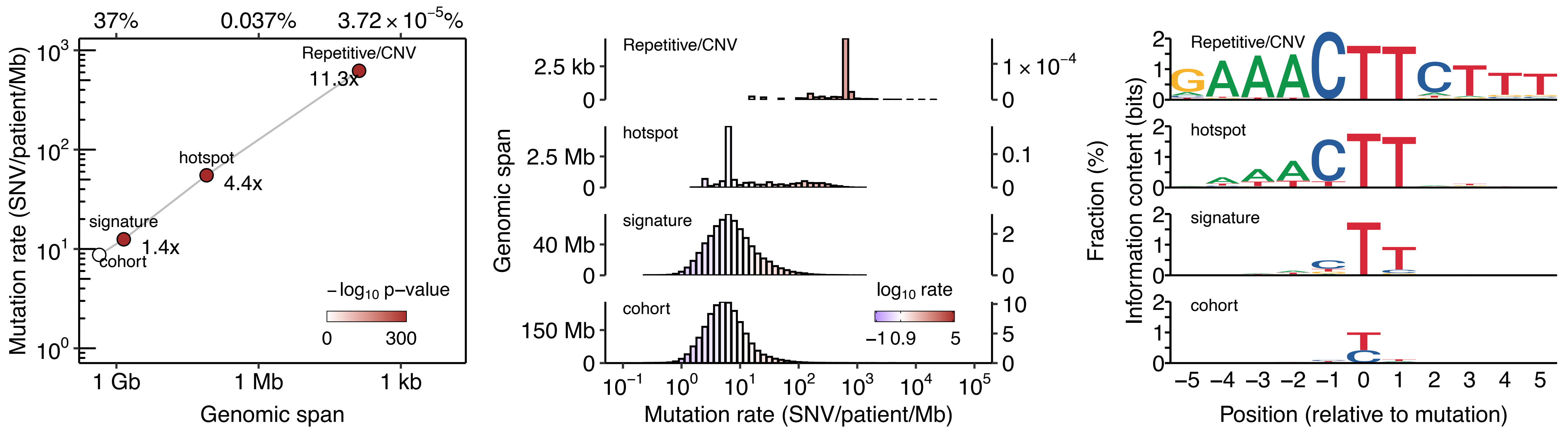

Signature 18

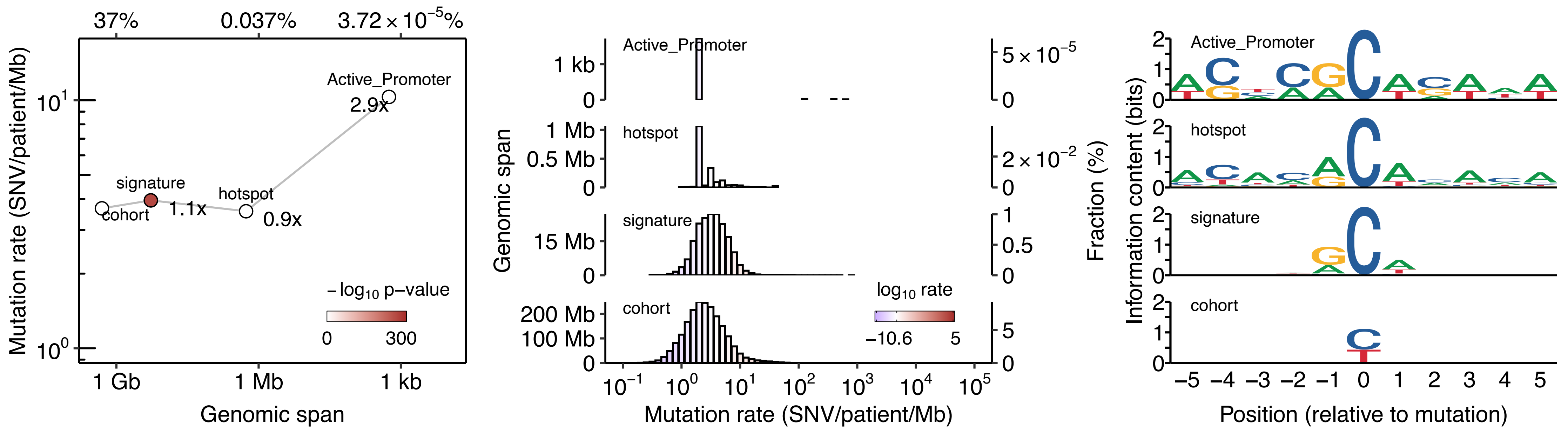

Signature 19

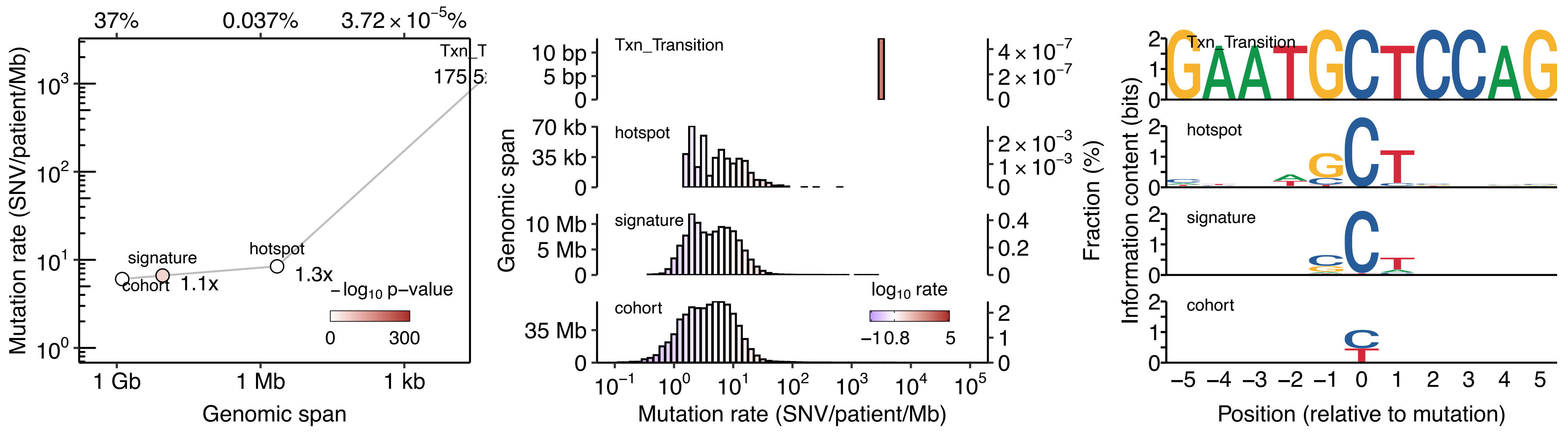

Signature 21

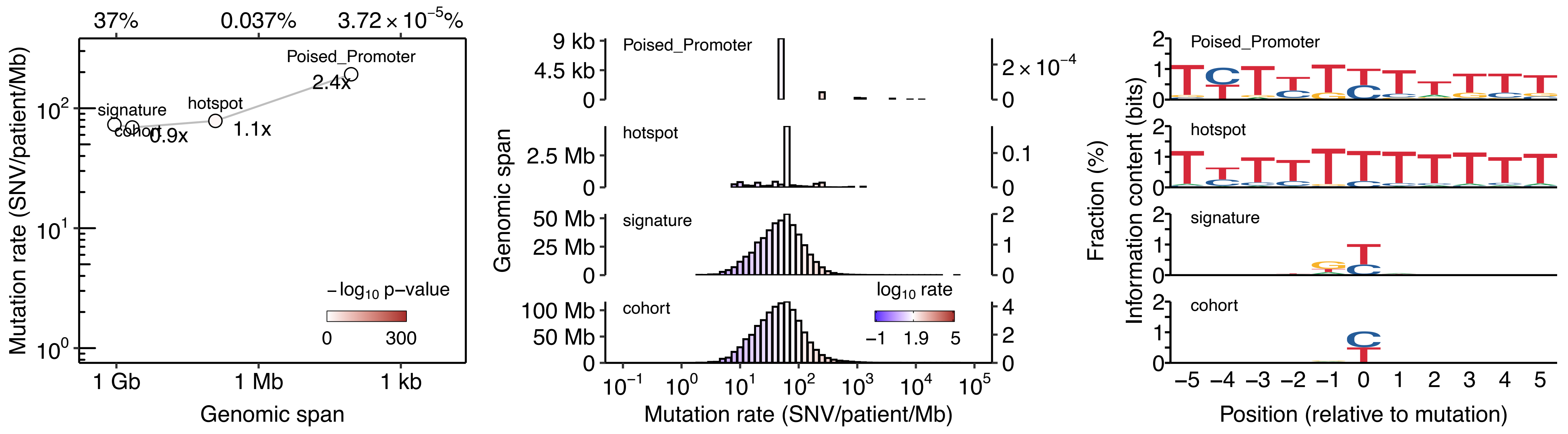

Signature 22

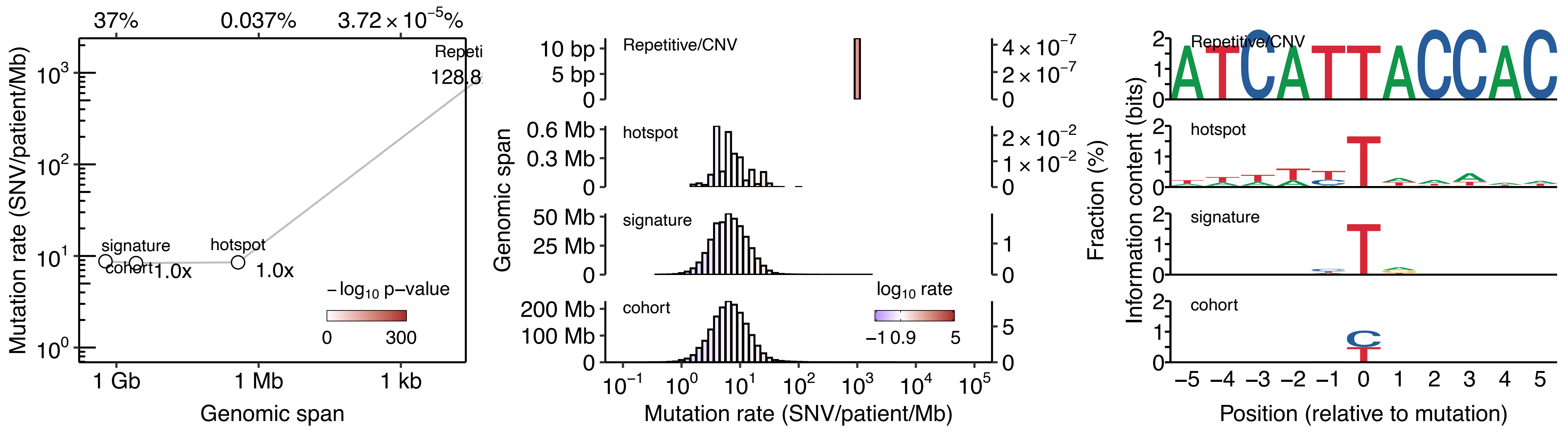

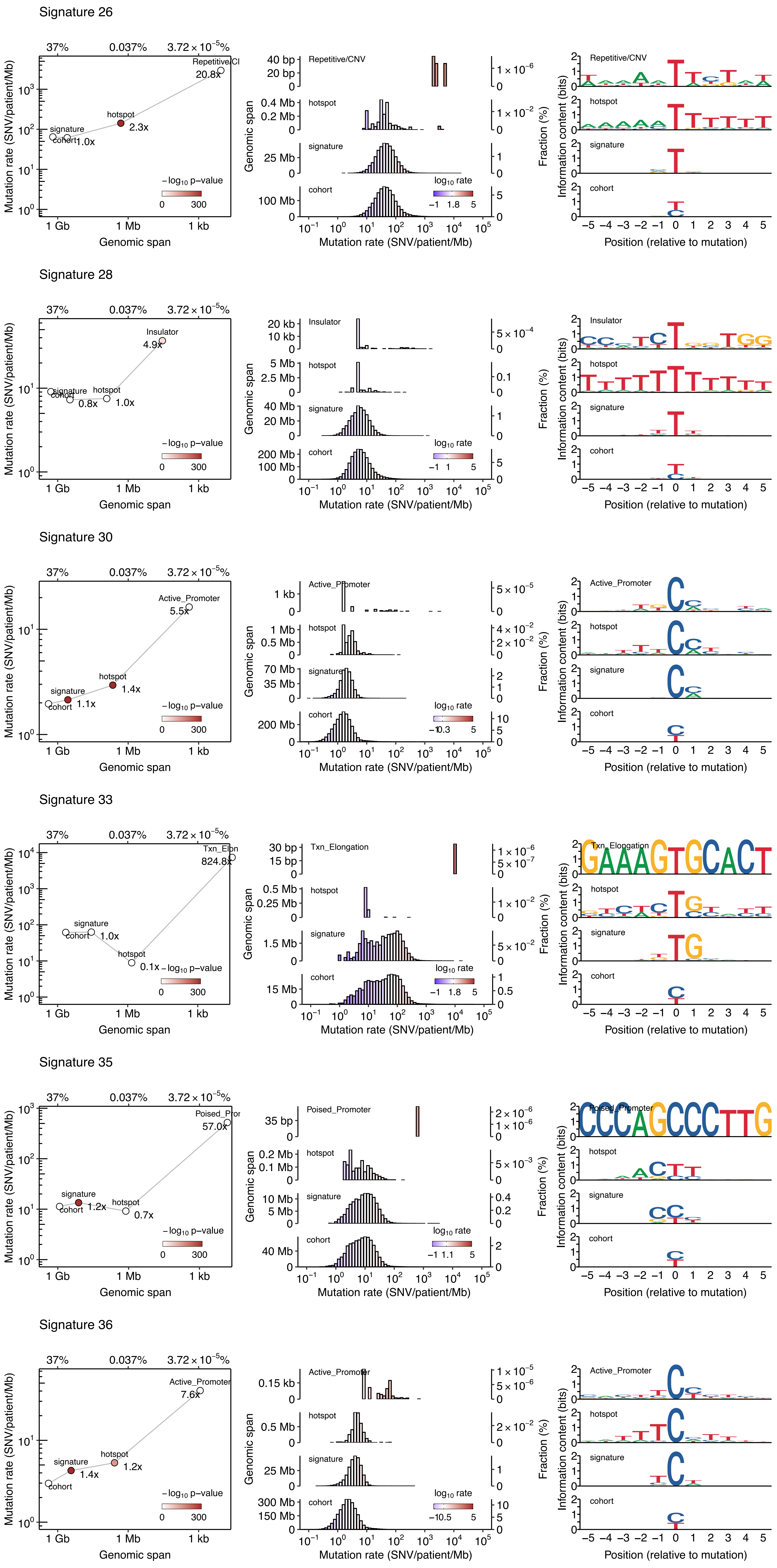

Signature 26
Repetitive/CNV
hotspot
signature
cohort
Mutation rate (SNV/patient/Mb)
Genomic span
Mutation rate (SNV/patient/Mb)
Fraction (%)
Information content (bits)
Position (relative to mutation)
−log10 p-value
log10 rate
Signature 28
Insulator
Signature 30
Active_Promoter
Signature 33
Txn_Elongation
Signature 35
Poised_Promoter
Signature 36
Active_Promoter

Signature 37

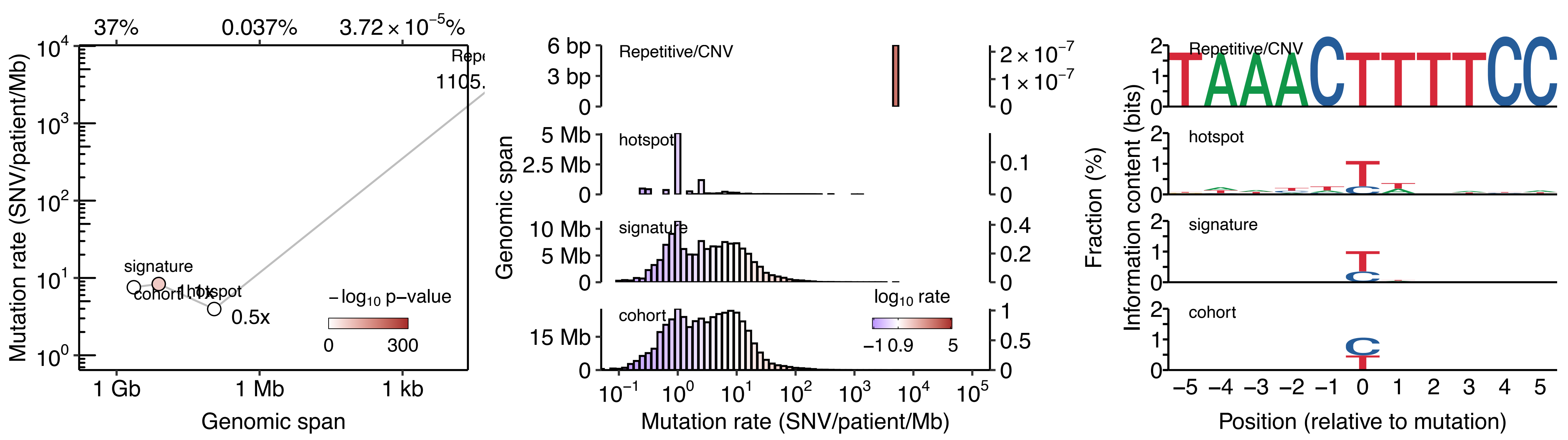

Signature 38

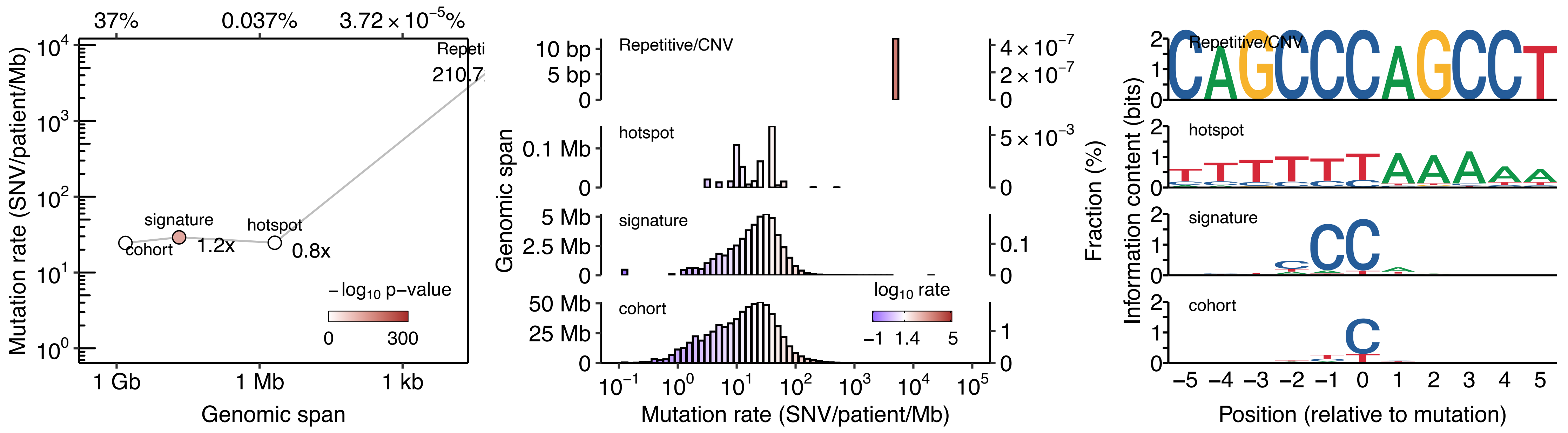

Signature 39

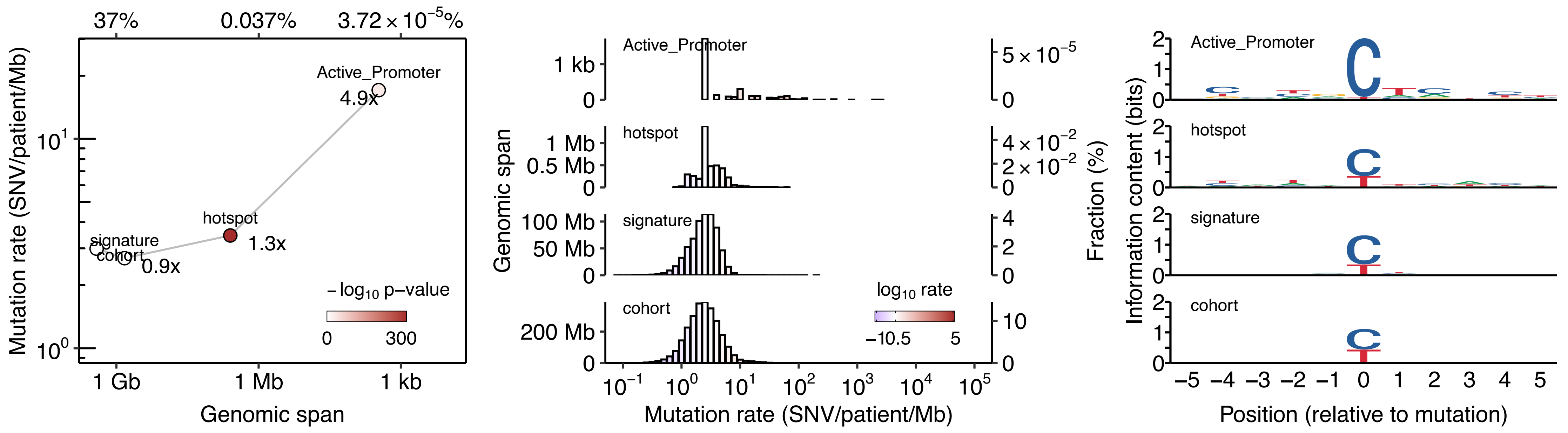

Signature 40

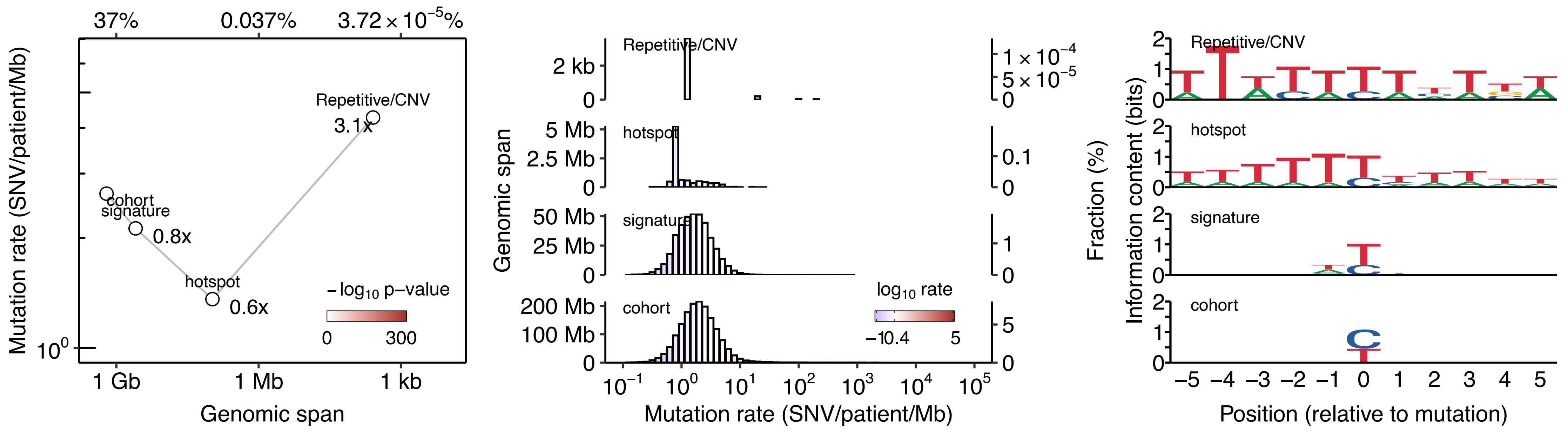

Signature 55

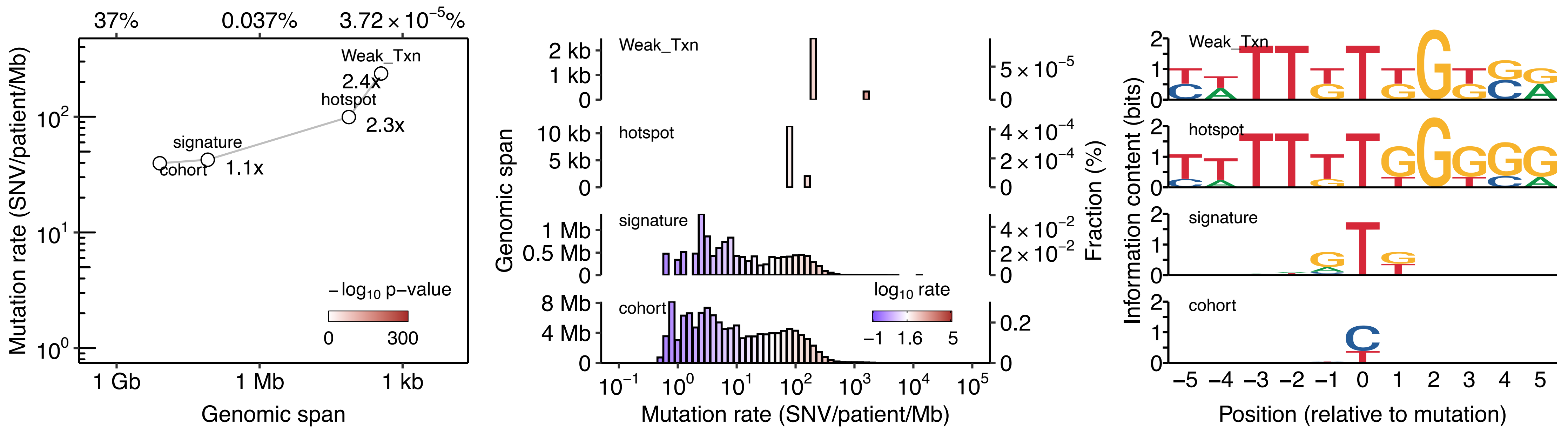

Signature 44

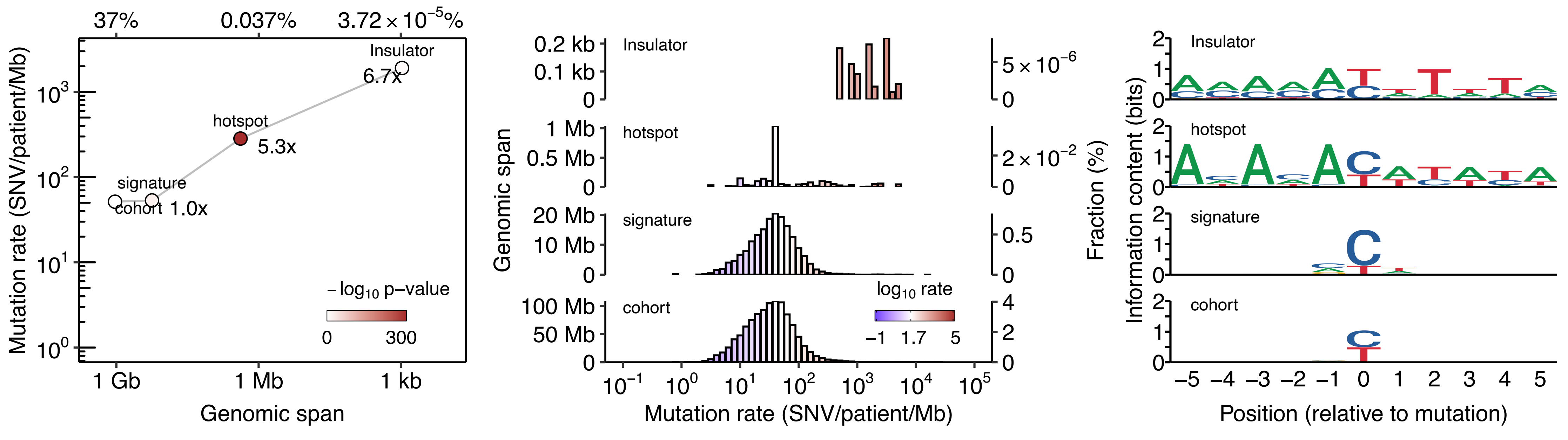

Signature 61

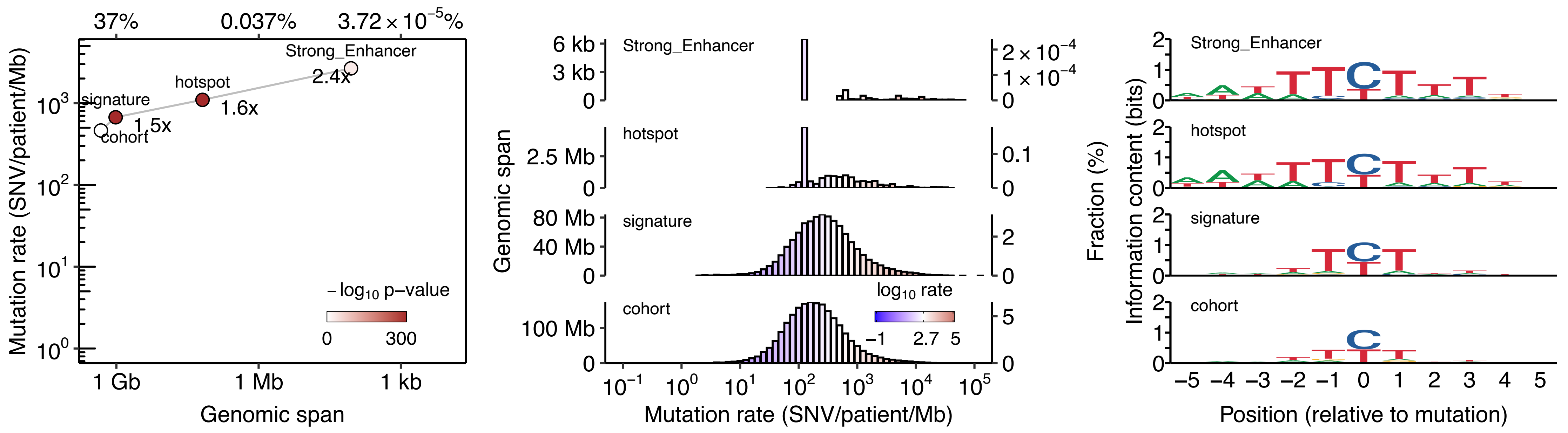

Signature 62

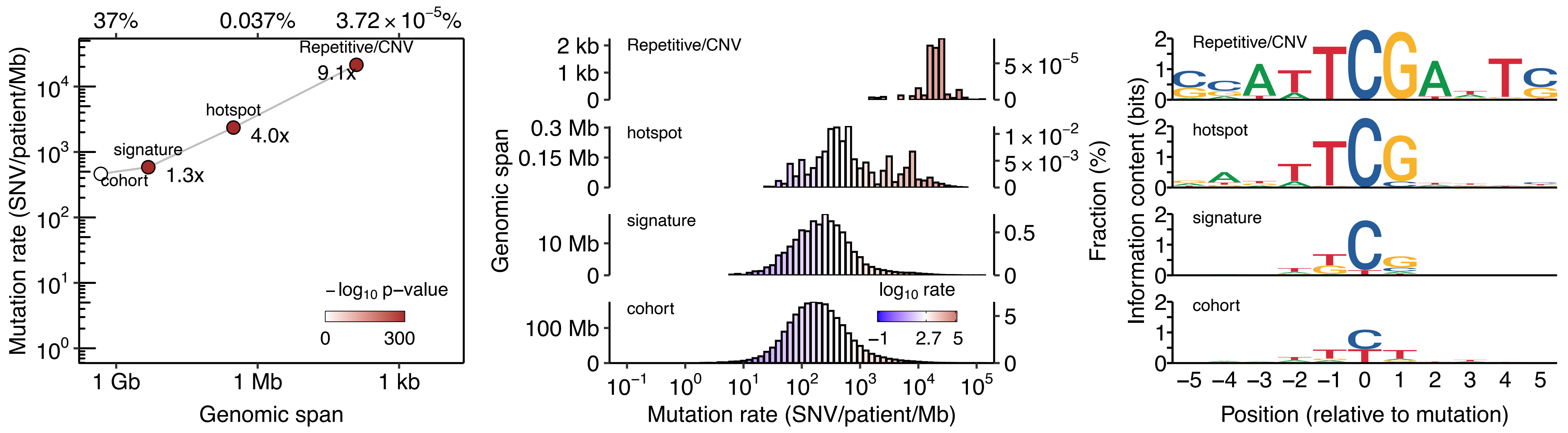

Signature 63

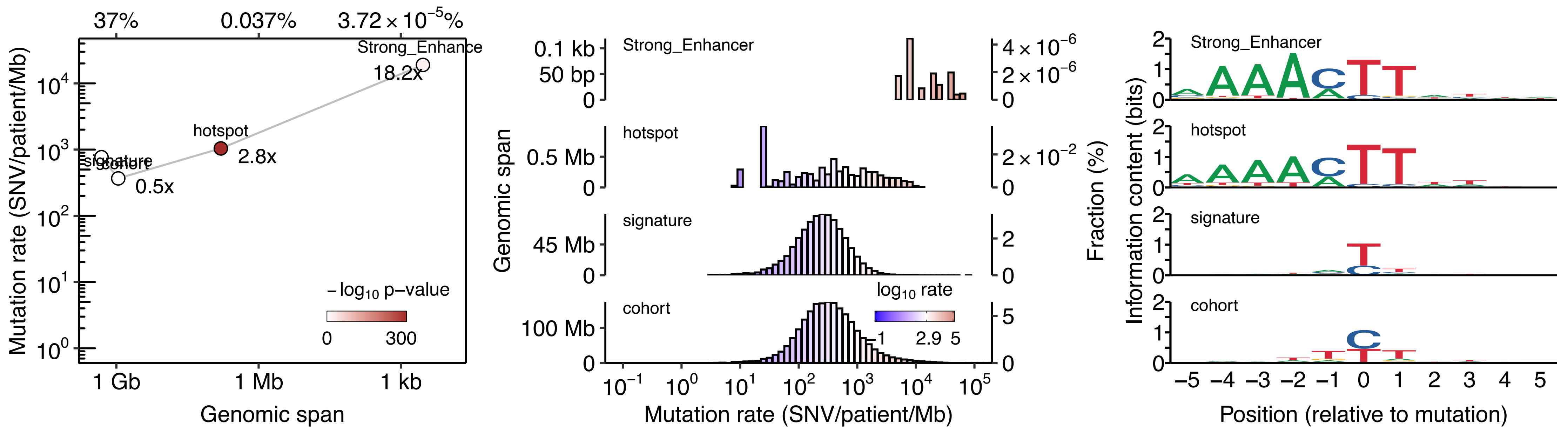

Signature 64

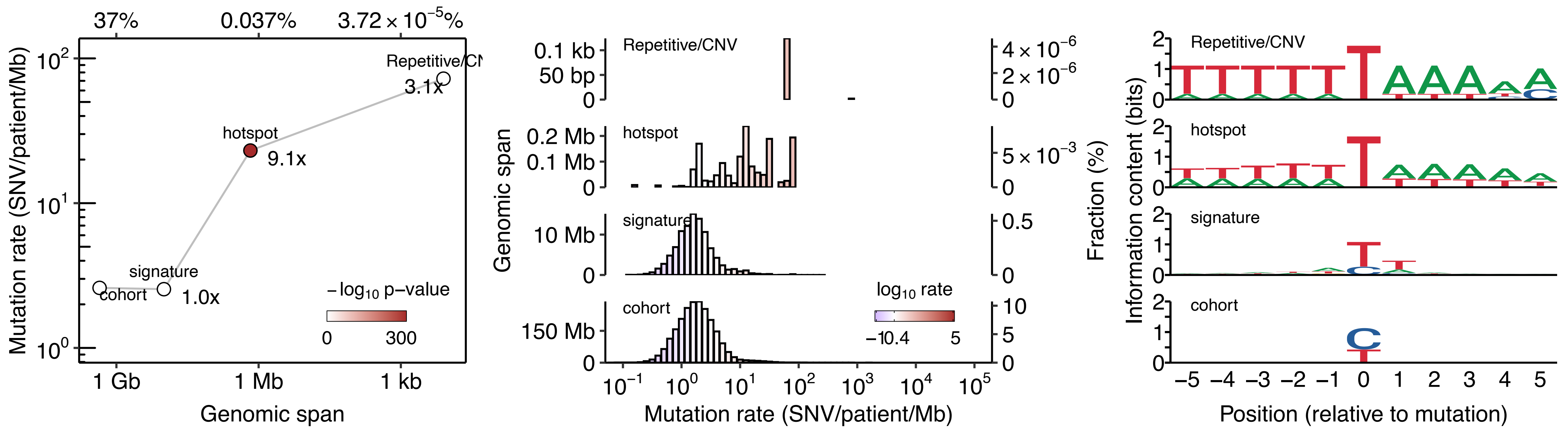

Signature 65

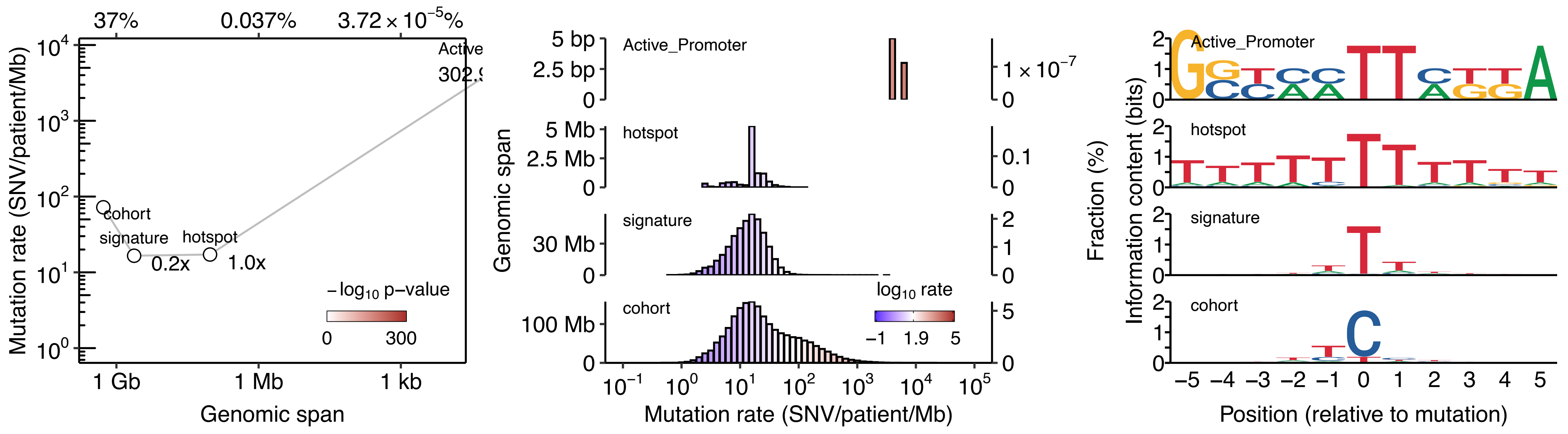

Signature 66

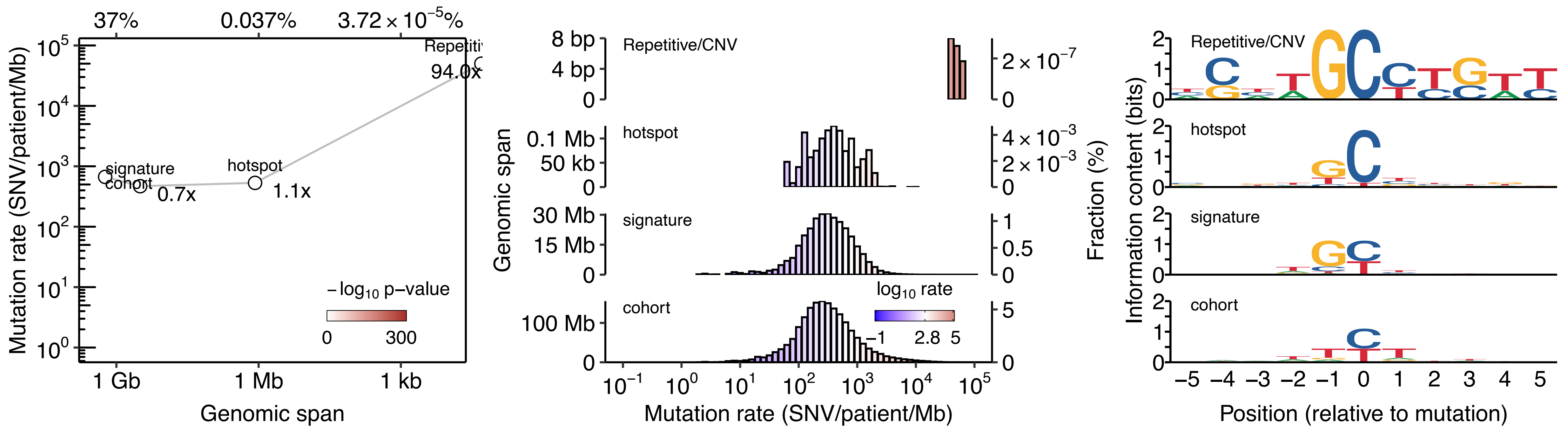

Signature 67

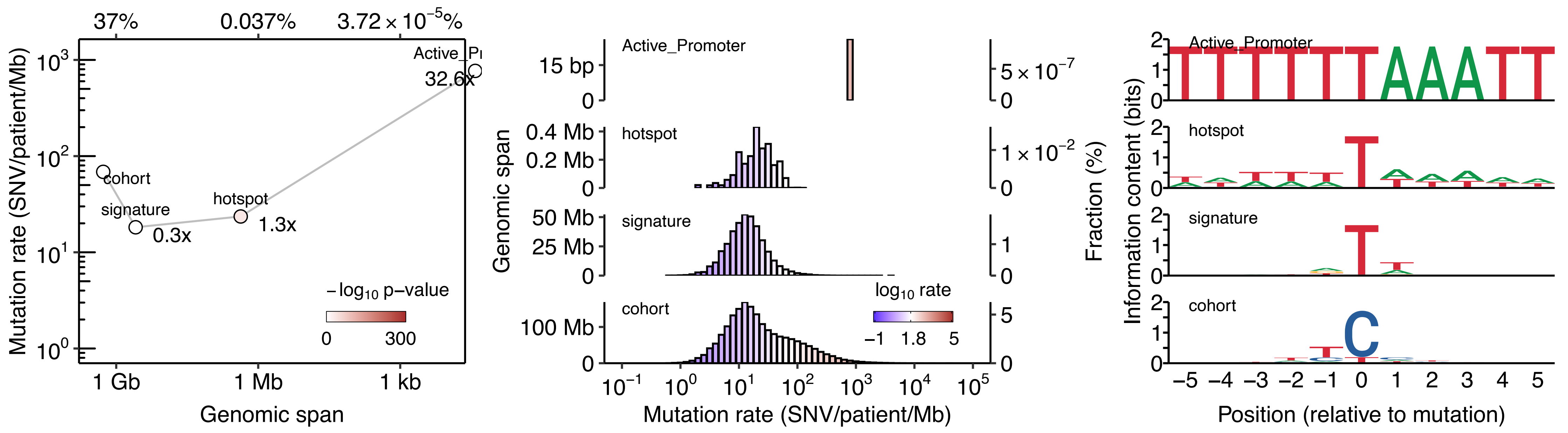

Signature 68

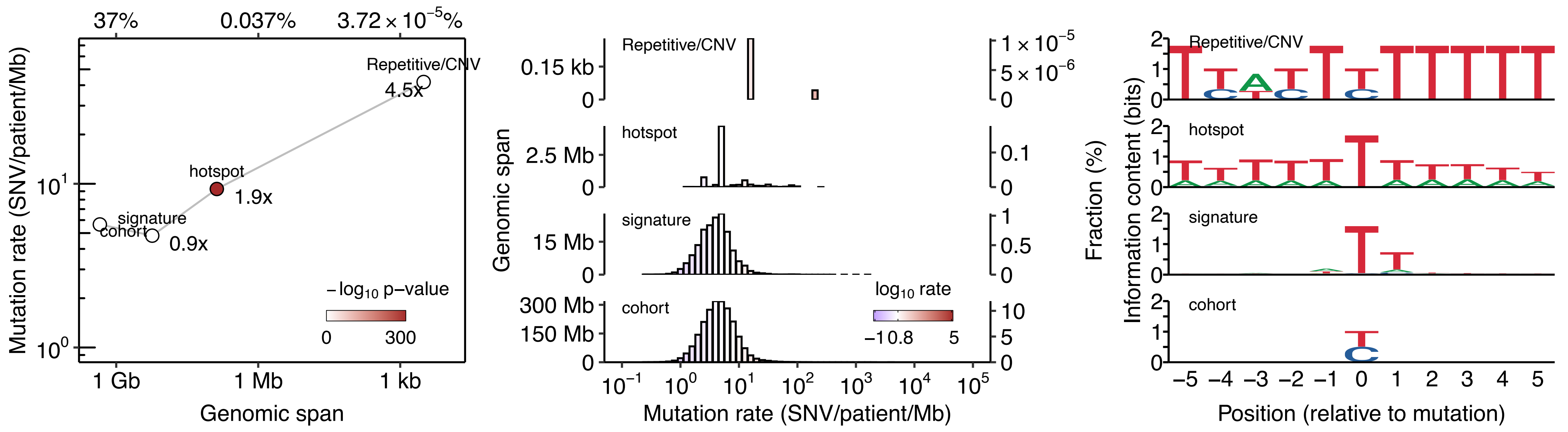

Signature 69

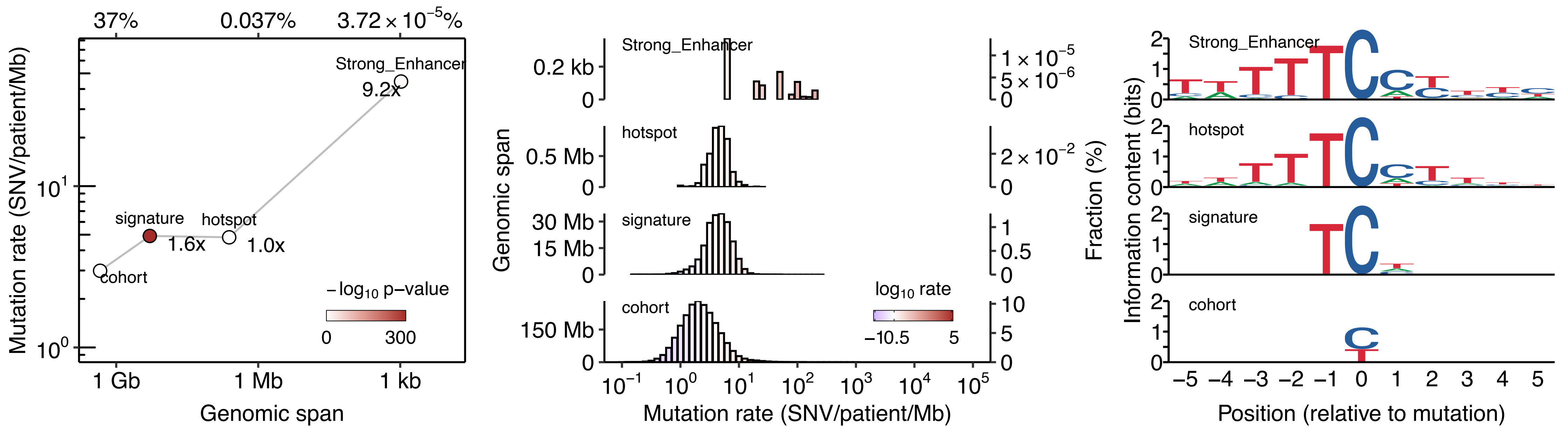

Signature 70

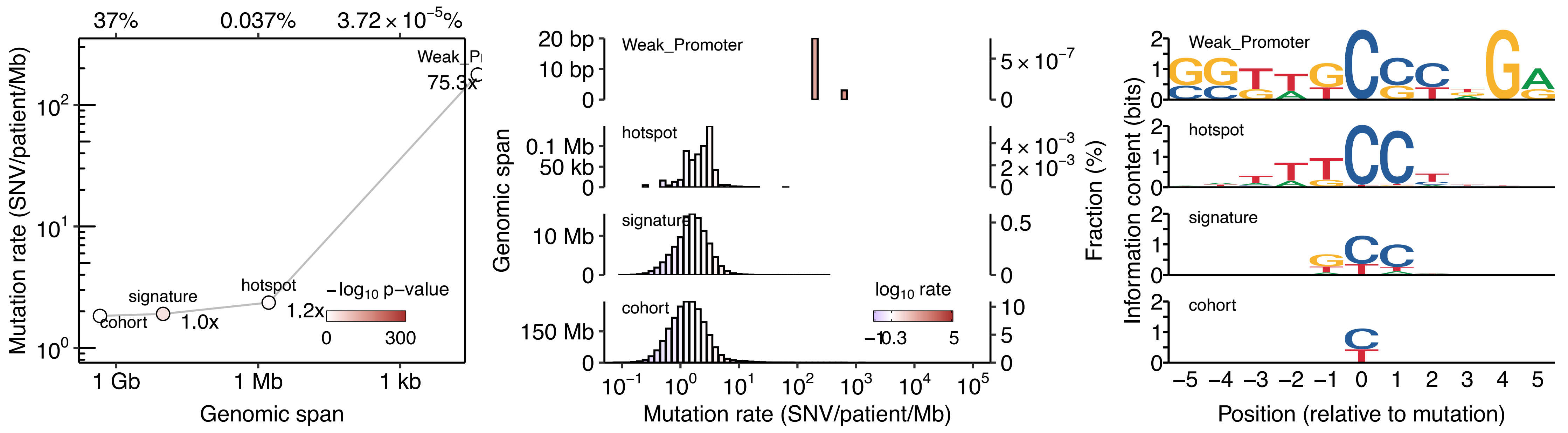

Signature 71

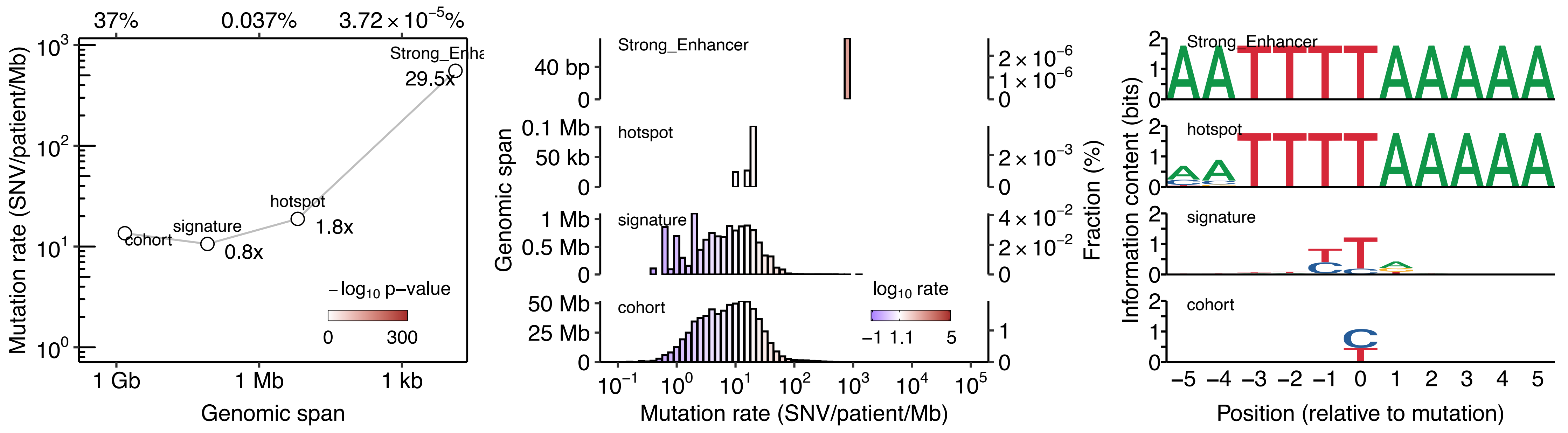

Signature 72

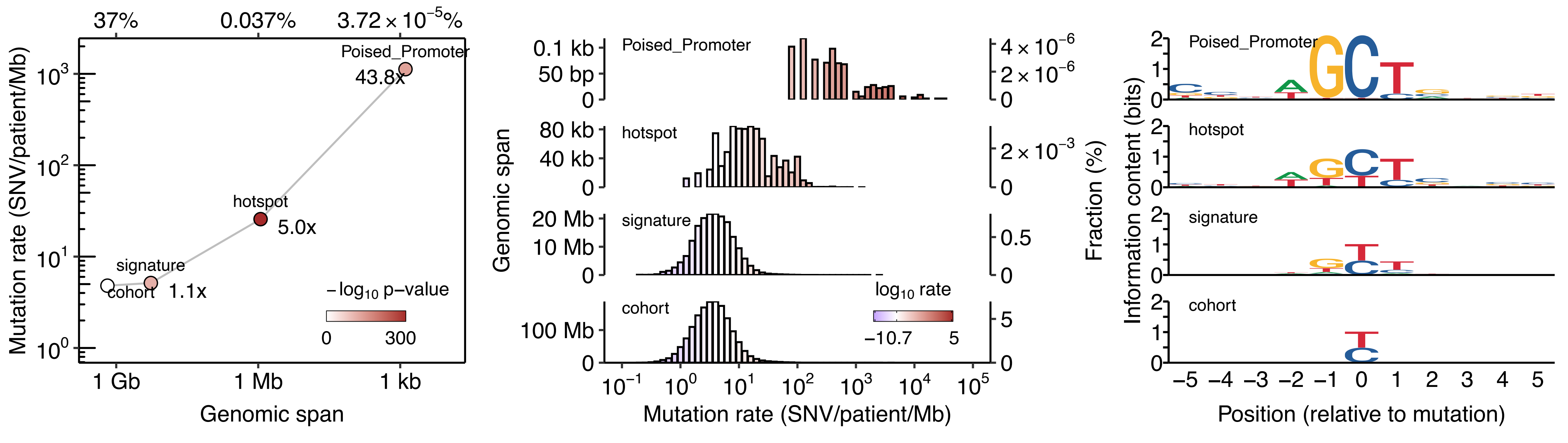

Signature 73

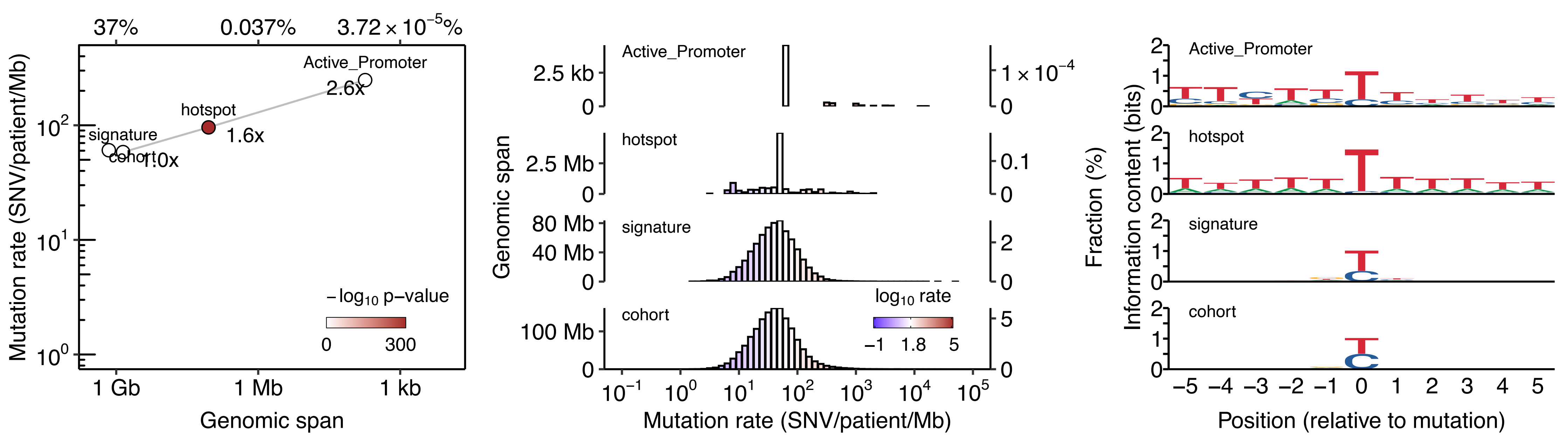

Signature 74

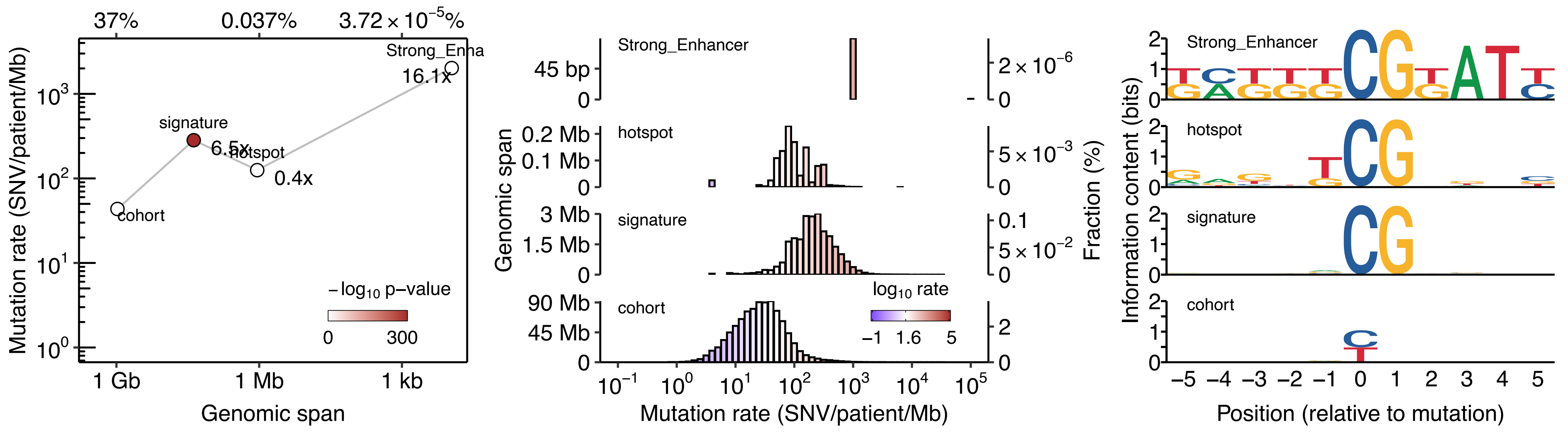

Signature 75

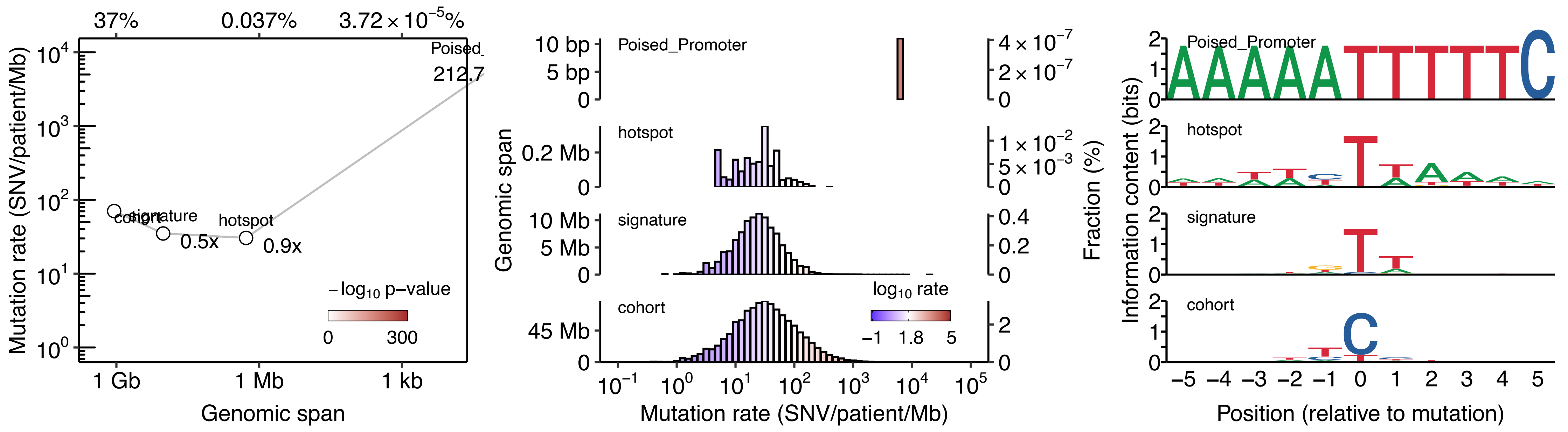

Signature 76

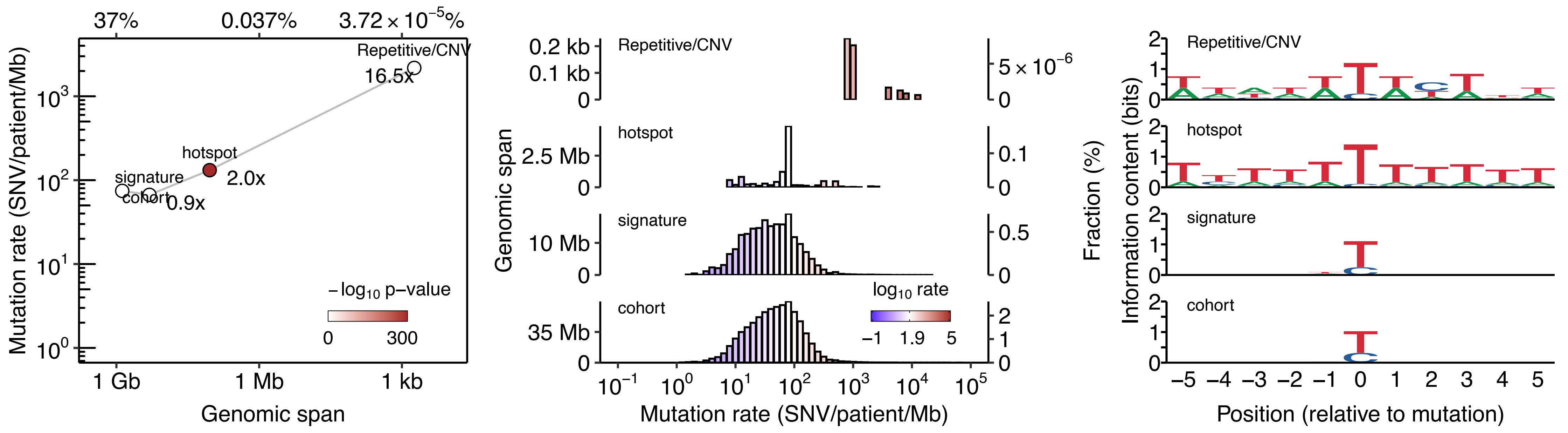

Signature 77

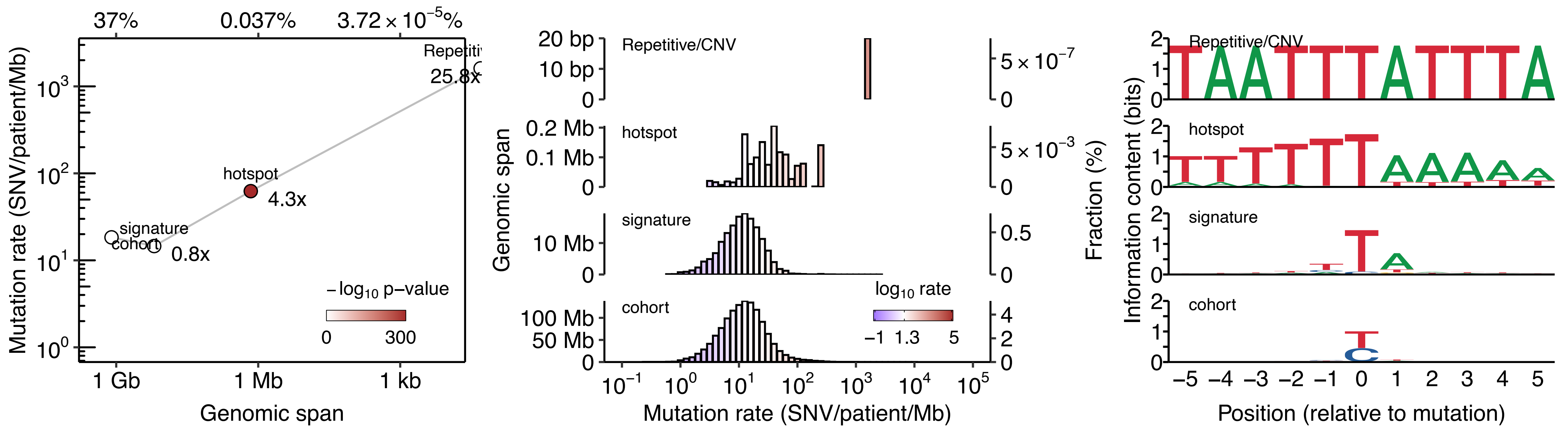

Signature 78

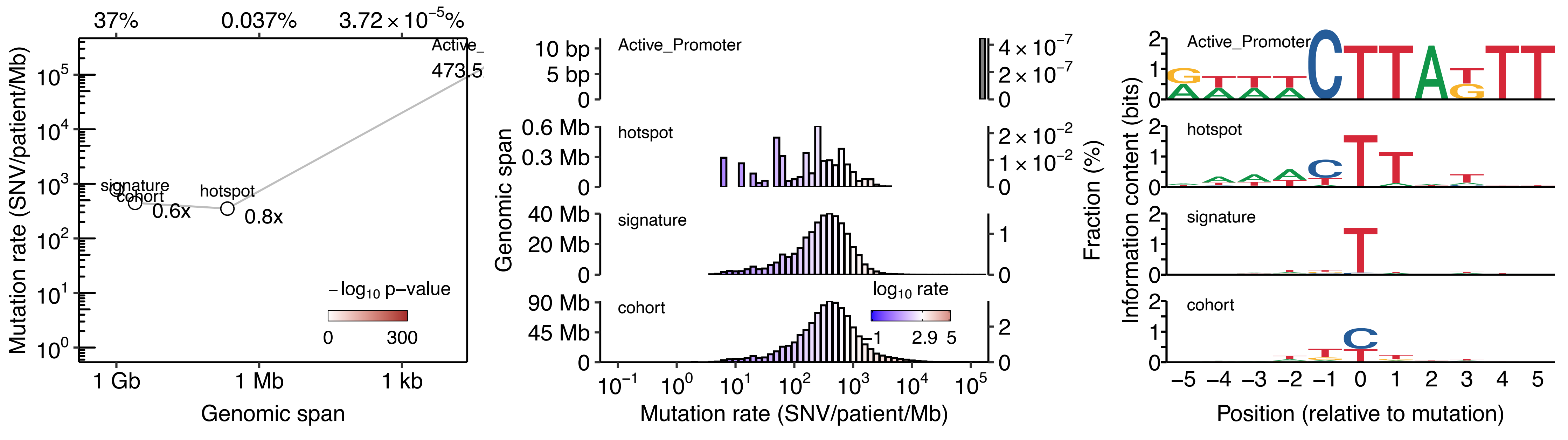

Figure S1. Catalog of localized mutational processes and their mutation rates.

(**Left panel**) The decreasing genomic spans (x-axis) and increasing mutation rates (y-axis) are shown for nested genomic subsets of 54 mutational signature cohorts (as in **Fig. 6**). The cohort mutation rate is based on the entire non-coding genome, followed by the signature assigned 11-mers, hotspot-associated 11-mers, and finally, the subset falling in the genomic region with the highest (significant) observed mutation rate. The relative mutation rate increase from the prior set is shown and its significance indicated (red color-scale; Bonferroni corrected p-value based on all 817 tests in full study; see **Fig. S2** for specific values). The overall total rate change compared with the cohort is given parenthetically. Mutation rate confidence intervals (CI-99%) are narrow and therefore invisible. (**Middle panel**) The genomic spans (y-axis) of genomic positions binned by their mutation rates (x-axis; log-scale) for the cohort, signature, hotspot, and genomic region subsets as defined above. The level of a mutation rate increase (red) or decrease (blue) is shown relative to the mean cohort mutation rate (white). (**Right panels**) Sequence information content surrounding the SNVs for each of the four genomic subsets defined in a.

Figure S2

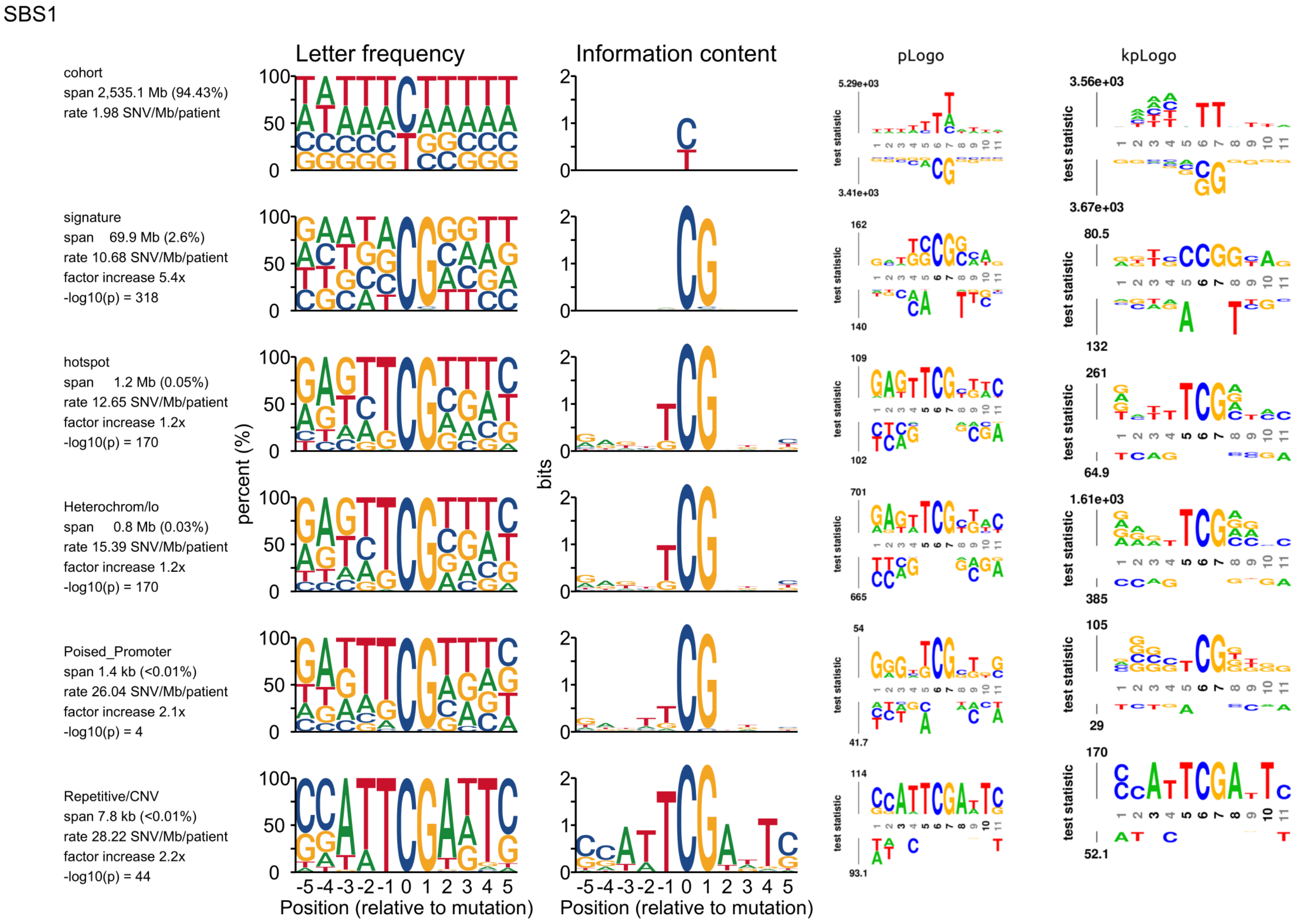
SBS1
Letter frequency
Information content
pLogo
kpLogo
cohort
span 2,535.1 Mb (94.43%)
rate 1.98 SNV/Mb/patient
signature
span 69.9 Mb (2.6%)
rate 10.68 SNV/Mb/patient
factor increase 5.4x
-log10(p) = 318
hotspot
span 1.2 Mb (0.05%)
rate 12.65 SNV/Mb/patient
factor increase 1.2x
-log10(p) = 170
Heterochrom/lo
span 0.8 Mb (0.03%)
rate 15.39 SNV/Mb/patient
factor increase 1.2x
-log10(p) = 170
Poised_Promoter
span 1.4 kb (<0.01%)
rate 26.04 SNV/Mb/patient
factor increase 2.1x
-log10(p) = 4
Repetitive/CNV
span 7.8 kb (<0.01%)
rate 28.22 SNV/Mb/patient
factor increase 2.2x
-log10(p) = 44
percent (%)
bits
test statistic
Position (relative to mutation)
5.29e+03
3.41e+03
3.56e+03
3.67e+03
162
140
80.5
132
109
102
261
64.9
701
665
1.61e+03
385
54
41.7
105
29
114
93.1
170
52.1

SBS2

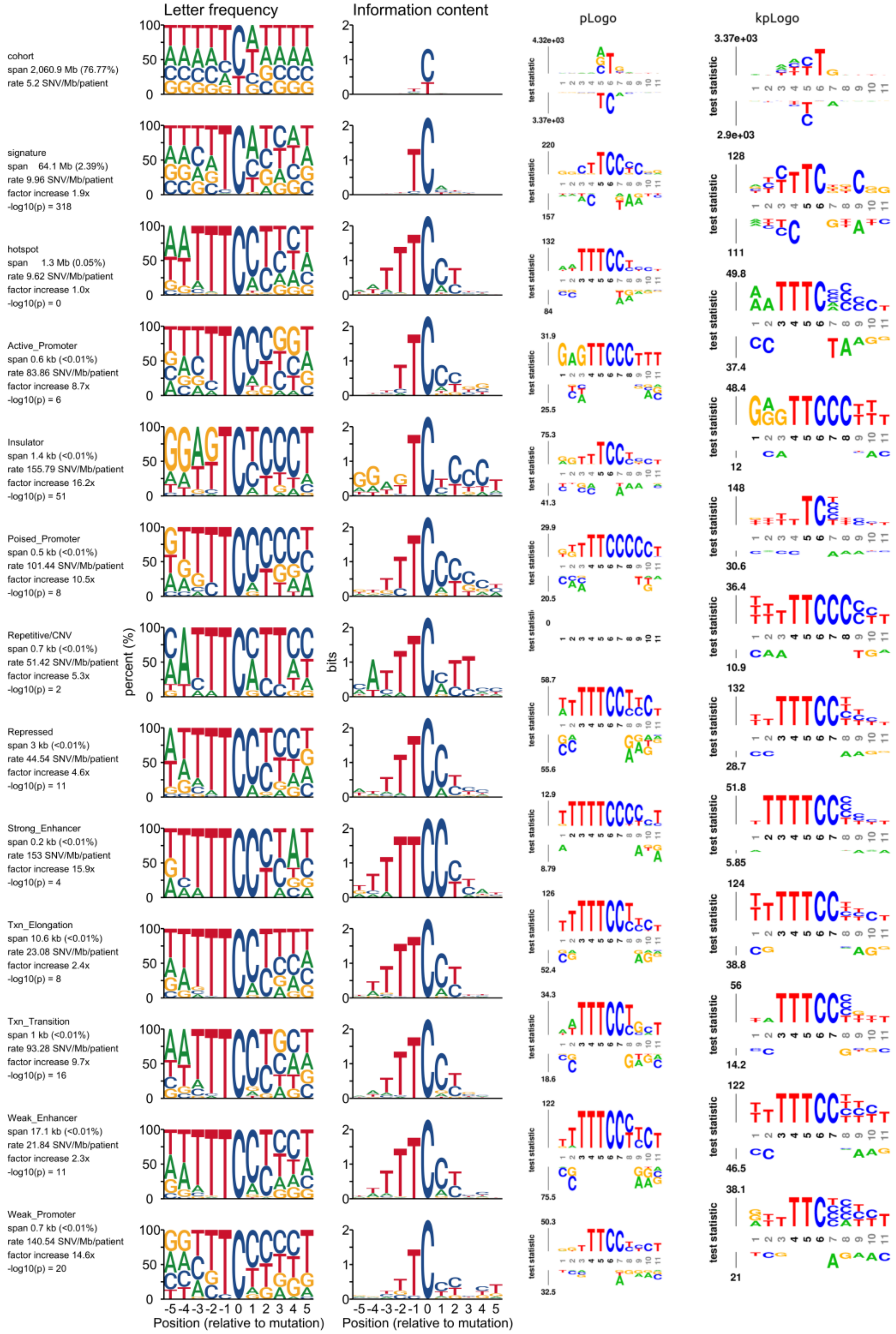
Letter frequency
Information content
pLogo
kpLogo
cohort
span 2,060.9 Mb (76.77%)
rate 5.2 SNV/Mb/patient
signature
span 64.1 Mb (2.39%)
rate 9.96 SNV/Mb/patient
factor increase 1.9x
-log10(p) = 318
hotspot
span 1.3 Mb (0.05%)
rate 9.62 SNV/Mb/patient
factor increase 1.0x
-log10(p) = 0
Active_Promoter
span 0.6 kb (<0.01%)
rate 83.86 SNV/Mb/patient
factor increase 8.7x
-log10(p) = 6
Insulator
span 1.4 kb (<0.01%)
rate 155.79 SNV/Mb/patient
factor increase 16.2x
-log10(p) = 51
Poised_Promoter
span 0.5 kb (<0.01%)
rate 101.44 SNV/Mb/patient
factor increase 10.5x
-log10(p) = 8
Repetitive/CNV
span 0.7 kb (<0.01%)
rate 51.42 SNV/Mb/patient
factor increase 5.3x
-log10(p) = 2
Repressed
span 3 kb (<0.01%)
rate 44.54 SNV/Mb/patient
factor increase 4.6x
-log10(p) = 11
Strong_Enhancer
span 0.2 kb (<0.01%)
rate 153 SNV/Mb/patient
factor increase 15.9x
-log10(p) = 4
Txn_Elongation
span 10.6 kb (<0.01%)
rate 23.08 SNV/Mb/patient
factor increase 2.4x
-log10(p) = 8
Txn_Transition
span 1 kb (<0.01%)
rate 93.28 SNV/Mb/patient
factor increase 9.7x
-log10(p) = 16
Weak_Enhancer
span 17.1 kb (<0.01%)
rate 21.84 SNV/Mb/patient
factor increase 2.3x
-log10(p) = 11
Weak_Promoter
span 0.7 kb (<0.01%)
rate 140.54 SNV/Mb/patient
factor increase 14.6x
-log10(p) = 20
percent (%)
bits
test statistic
Position (relative to mutation)
Position (relative to mutation)

SBS4

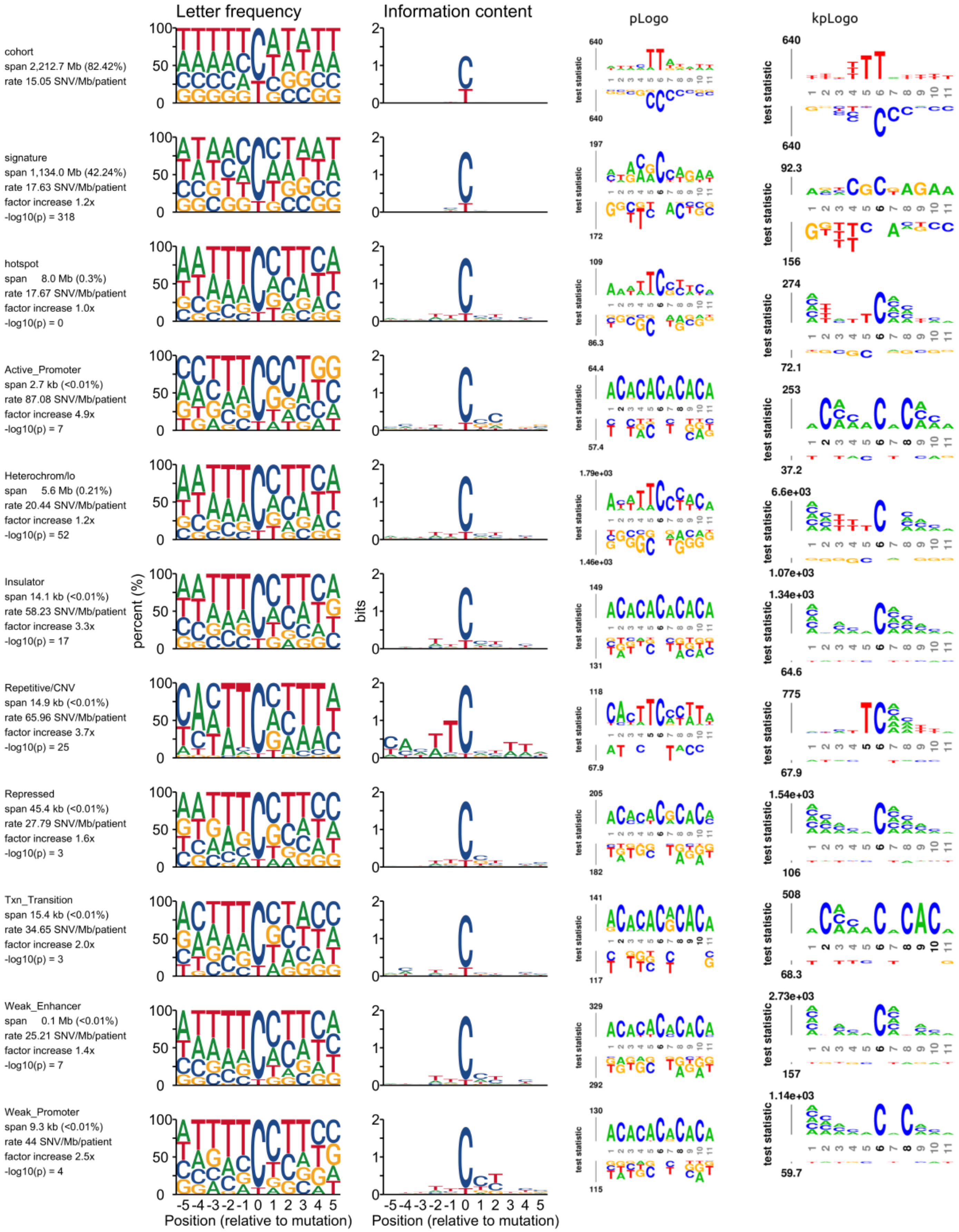
Letter frequency
Information content
pLogo
kpLogo
cohort
span 2,212.7 Mb (82.42%)
rate 15.05 SNV/Mb/patient
signature
span 1,134.0 Mb (42.24%)
rate 17.63 SNV/Mb/patient
factor increase 1.2x
-log10(p) = 318
hotspot
span 8.0 Mb (0.3%)
rate 17.67 SNV/Mb/patient
factor increase 1.0x
-log10(p) = 0
Active_Promoter
span 2.7 kb (<0.01%)
rate 87.08 SNV/Mb/patient
factor increase 4.9x
-log10(p) = 7
Heterochrom/lo
span 5.6 Mb (0.21%)
rate 20.44 SNV/Mb/patient
factor increase 1.2x
-log10(p) = 52
Insulator
span 14.1 kb (<0.01%)
rate 58.23 SNV/Mb/patient
factor increase 3.3x
-log10(p) = 17
Repetitive/CNV
span 14.9 kb (<0.01%)
rate 65.96 SNV/Mb/patient
factor increase 3.7x
-log10(p) = 25
Repressed
span 45.4 kb (<0.01%)
rate 27.79 SNV/Mb/patient
factor increase 1.6x
-log10(p) = 3
Txn_Transition
span 15.4 kb (<0.01%)
rate 34.65 SNV/Mb/patient
factor increase 2.0x
-log10(p) = 3
Weak_Enhancer
span 0.1 Mb (<0.01%)
rate 25.21 SNV/Mb/patient
factor increase 1.4x
-log10(p) = 7
Weak_Promoter
span 9.3 kb (<0.01%)
rate 44 SNV/Mb/patient
factor increase 2.5x
-log10(p) = 4
percent (%)
bits
test statistic
Position (relative to mutation)
Position (relative to mutation)

SBS5

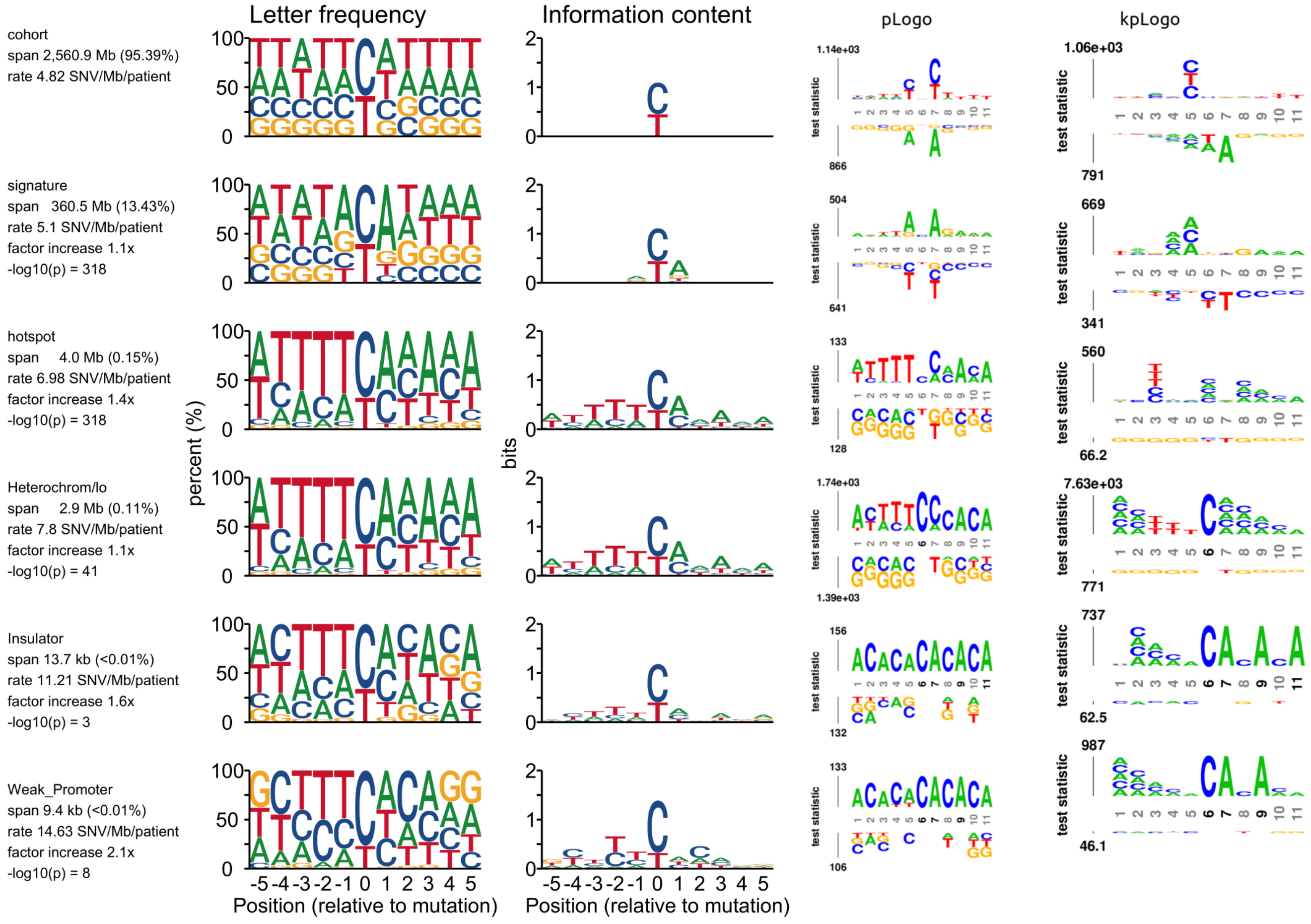
Letter frequency
Information content
pLogo
kpLogo
cohort
span 2,560.9 Mb (95.39%)
rate 4.82 SNV/Mb/patient
signature
span 360.5 Mb (13.43%)
rate 5.1 SNV/Mb/patient
factor increase 1.1x
-log10(p) = 318
hotspot
span 4.0 Mb (0.15%)
rate 6.98 SNV/Mb/patient
factor increase 1.4x
-log10(p) = 318
Heterochrom/lo
span 2.9 Mb (0.11%)
rate 7.8 SNV/Mb/patient
factor increase 1.1x
-log10(p) = 41
Insulator
span 13.7 kb (<0.01%)
rate 11.21 SNV/Mb/patient
factor increase 1.6x
-log10(p) = 3
Weak_Promoter
span 9.4 kb (<0.01%)
rate 14.63 SNV/Mb/patient
factor increase 2.1x
-log10(p) = 8
percent (%)
bits
test statistic
Position (relative to mutation)
Position (relative to mutation)

SBS6

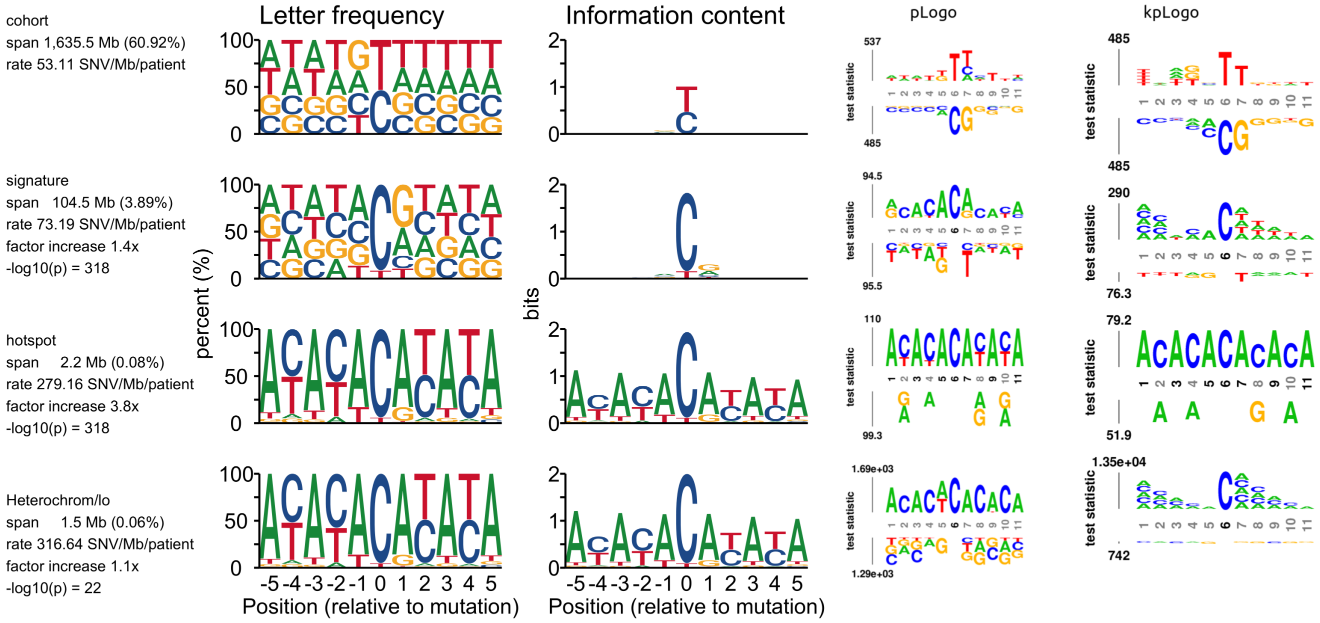
cohort
span 1,635.5 Mb (60.92%)
rate 53.11 SNV/Mb/patient
signature
span 104.5 Mb (3.89%)
rate 73.19 SNV/Mb/patient
factor increase 1.4x
-log10(p) = 318
hotspot
span 2.2 Mb (0.08%)
rate 279.16 SNV/Mb/patient
factor increase 3.8x
-log10(p) = 318
Heterochrom/lo
span 1.5 Mb (0.06%)
rate 316.64 SNV/Mb/patient
factor increase 1.1x
-log10(p) = 22
Letter frequency
Information content
pLogo
kpLogo
percent (%)
bits
test statistic
Position (relative to mutation)
Position (relative to mutation)

SBS7a

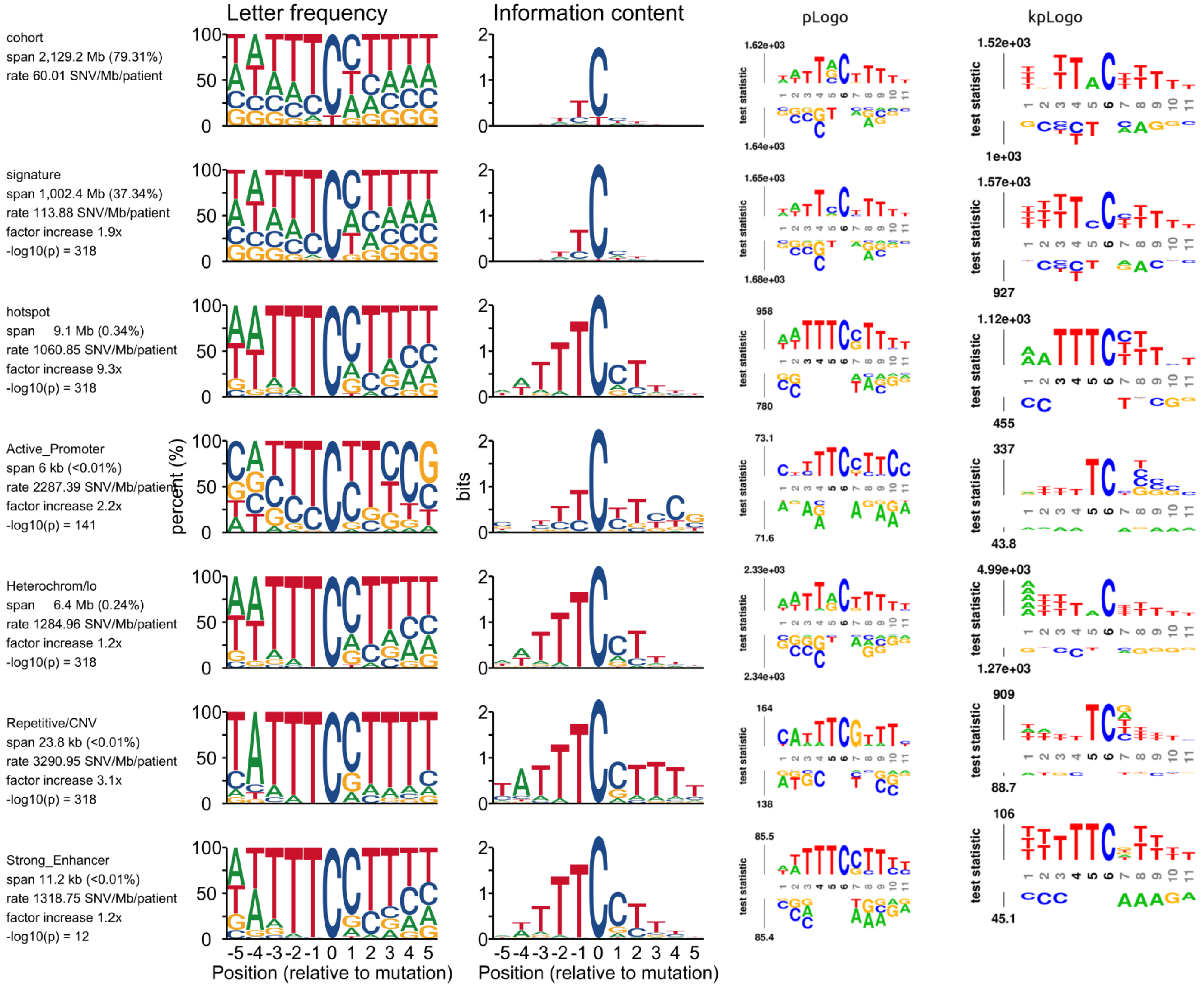
Letter frequency
Information content
pLogo
kpLogo
cohort
span 2,129.2 Mb (79.31%)
rate 60.01 SNV/Mb/patient
signature
span 1,002.4 Mb (37.34%)
rate 113.88 SNV/Mb/patient
factor increase 1.9x
-log10(p) = 318
hotspot
span 9.1 Mb (0.34%)
rate 1060.85 SNV/Mb/patient
factor increase 9.3x
-log10(p) = 318
Active_Promoter
span 6 kb (<0.01%)
rate 2287.39 SNV/Mb/patient
factor increase 2.2x
-log10(p) = 141
Heterochrom/lo
span 6.4 Mb (0.24%)
rate 1284.96 SNV/Mb/patient
factor increase 1.2x
-log10(p) = 318
Repetitive/CNV
span 23.8 kb (<0.01%)
rate 3290.95 SNV/Mb/patient
factor increase 3.1x
-log10(p) = 318
Strong_Enhancer
span 11.2 kb (<0.01%)
rate 1318.75 SNV/Mb/patient
factor increase 1.2x
-log10(p) = 12
percent (%)
bits
test statistic
Position (relative to mutation)
Position (relative to mutation)
1.62e+03
1.64e+03
1.52e+03
1e+03
1.65e+03
1.68e+03
1.57e+03
927
958
780
1.12e+03
455
73.1
71.6
337
43.8
2.33e+03
2.34e+03
4.99e+03
1.27e+03
164
138
909
88.7
85.5
85.4
106
45.1

SBS7b

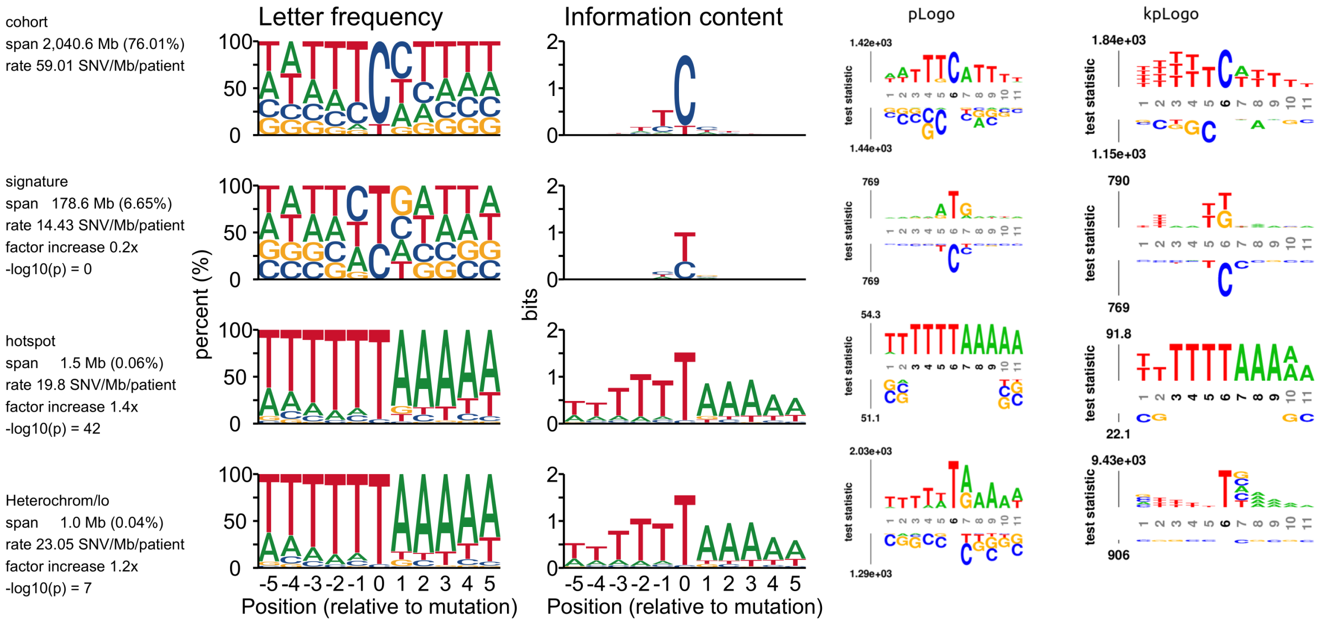
cohort
span 2,040.6 Mb (76.01%)
rate 59.01 SNV/Mb/patient
signature
span 178.6 Mb (6.65%)
rate 14.43 SNV/Mb/patient
factor increase 0.2x
-log10(p) = 0
hotspot
span 1.5 Mb (0.06%)
rate 19.8 SNV/Mb/patient
factor increase 1.4x
-log10(p) = 42
Heterochrom/lo
span 1.0 Mb (0.04%)
rate 23.05 SNV/Mb/patient
factor increase 1.2x
-log10(p) = 7
Letter frequency
Information content
pLogo
kpLogo
percent (%)
bits
test statistic
Position (relative to mutation)
Position (relative to mutation)

SBS7c

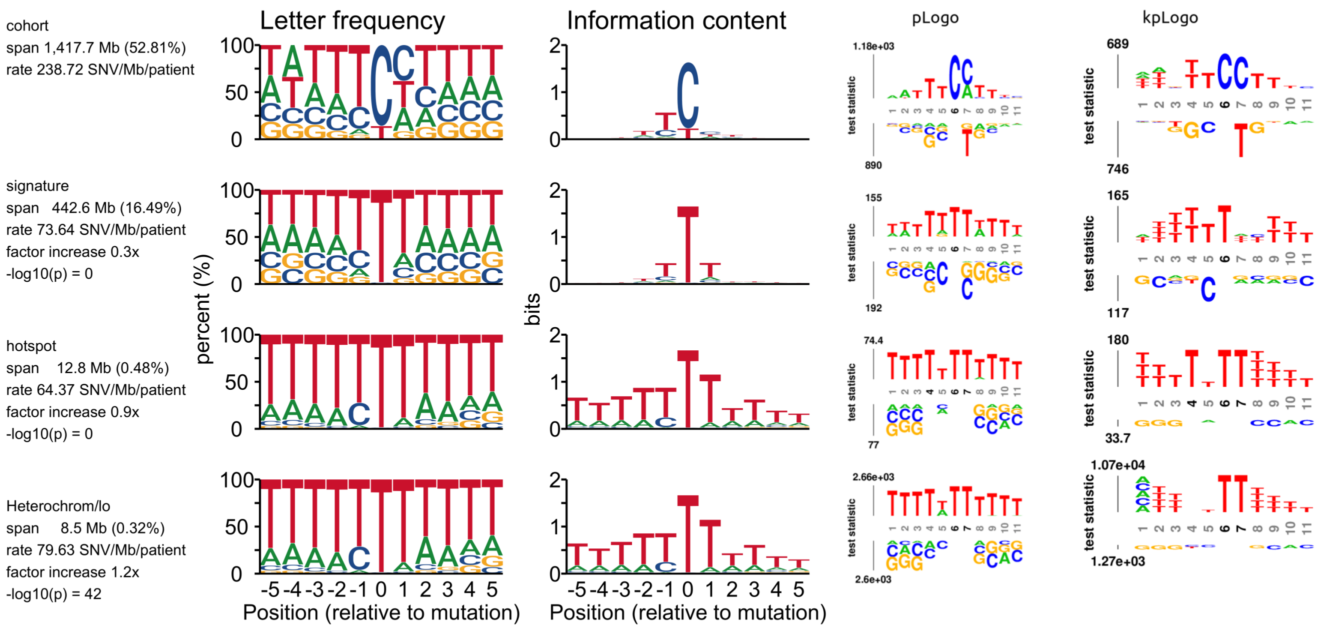
cohort
span 1,417.7 Mb (52.81%)
rate 238.72 SNV/Mb/patient
signature
span 442.6 Mb (16.49%)
rate 73.64 SNV/Mb/patient
factor increase 0.3x
-log10(p) = 0
hotspot
span 12.8 Mb (0.48%)
rate 64.37 SNV/Mb/patient
factor increase 0.9x
-log10(p) = 0
Heterochrom/lo
span 8.5 Mb (0.32%)
rate 79.63 SNV/Mb/patient
factor increase 1.2x
-log10(p) = 42
Letter frequency
Information content
pLogo
kpLogo
percent (%)
bits
test statistic
Position (relative to mutation)
Position (relative to mutation)

SBS8

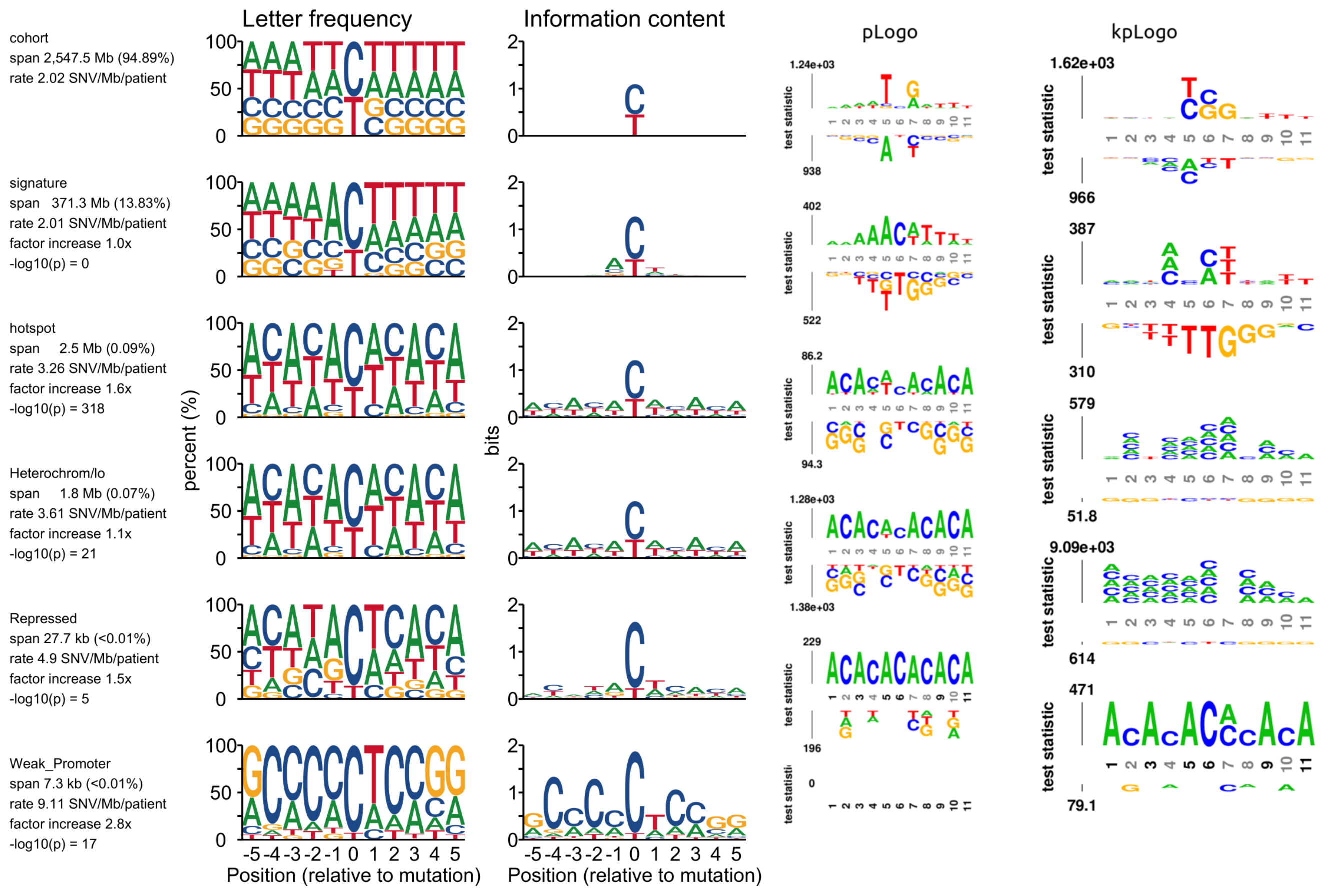
Letter frequency
Information content
pLogo
kpLogo
cohort
span 2,547.5 Mb (94.89%)
rate 2.02 SNV/Mb/patient
signature
span 371.3 Mb (13.83%)
rate 2.01 SNV/Mb/patient
factor increase 1.0x
-log10(p) = 0
hotspot
span 2.5 Mb (0.09%)
rate 3.26 SNV/Mb/patient
factor increase 1.6x
-log10(p) = 318
Heterochrom/lo
span 1.8 Mb (0.07%)
rate 3.61 SNV/Mb/patient
factor increase 1.1x
-log10(p) = 21
Repressed
span 27.7 kb (<0.01%)
rate 4.9 SNV/Mb/patient
factor increase 1.5x
-log10(p) = 5
Weak_Promoter
span 7.3 kb (<0.01%)
rate 9.11 SNV/Mb/patient
factor increase 2.8x
-log10(p) = 17
percent (%)
bits
Position (relative to mutation)
Position (relative to mutation)
test statistic
1.24e+03
938
402
522
86.2
94.3
1.28e+03
1.38e+03
229
196
0
1.62e+03
966
387
310
579
51.8
9.09e+03
614
471
79.1

SBS9

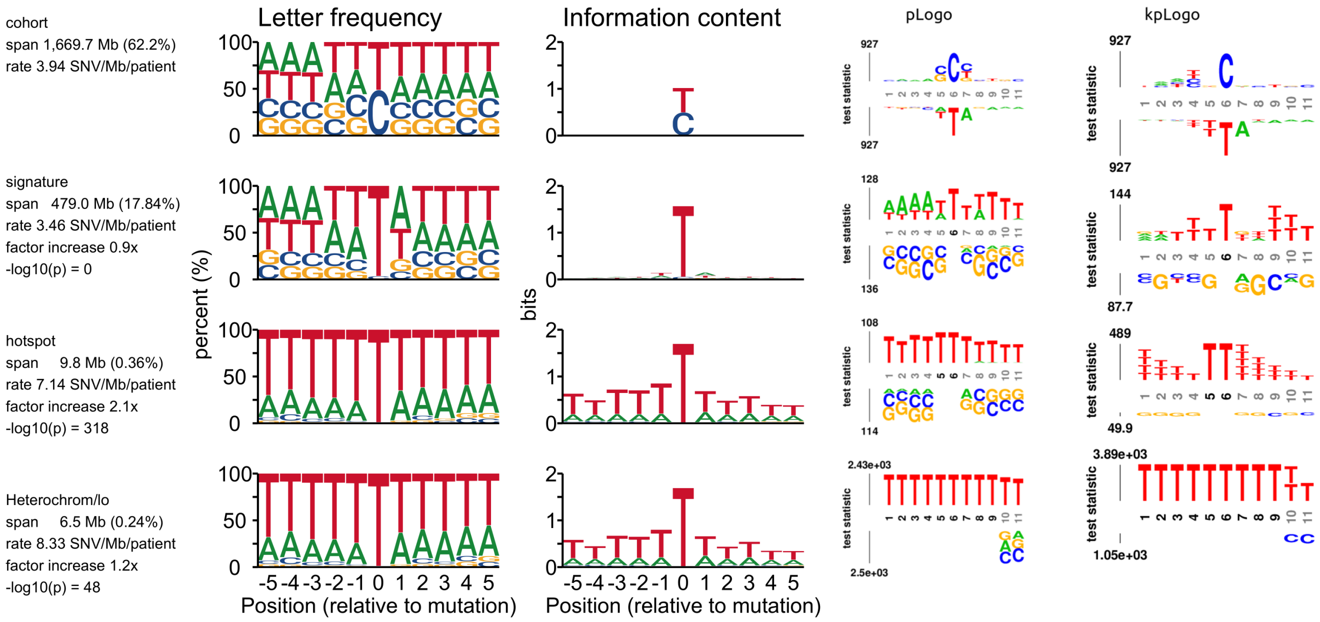
cohort
span 1,669.7 Mb (62.2%)
rate 3.94 SNV/Mb/patient
signature
span 479.0 Mb (17.84%)
rate 3.46 SNV/Mb/patient
factor increase 0.9x
-log10(p) = 0
hotspot
span 9.8 Mb (0.36%)
rate 7.14 SNV/Mb/patient
factor increase 2.1x
-log10(p) = 318
Heterochrom/lo
span 6.5 Mb (0.24%)
rate 8.33 SNV/Mb/patient
factor increase 1.2x
-log10(p) = 48
Letter frequency
Information content
pLogo
kpLogo
percent (%)
bits
test statistic
Position (relative to mutation)
927
128
136
108
114
2.43e+03
2.5e+03
144
87.7
489
49.9
3.89e+03
1.05e+03

SBS10a

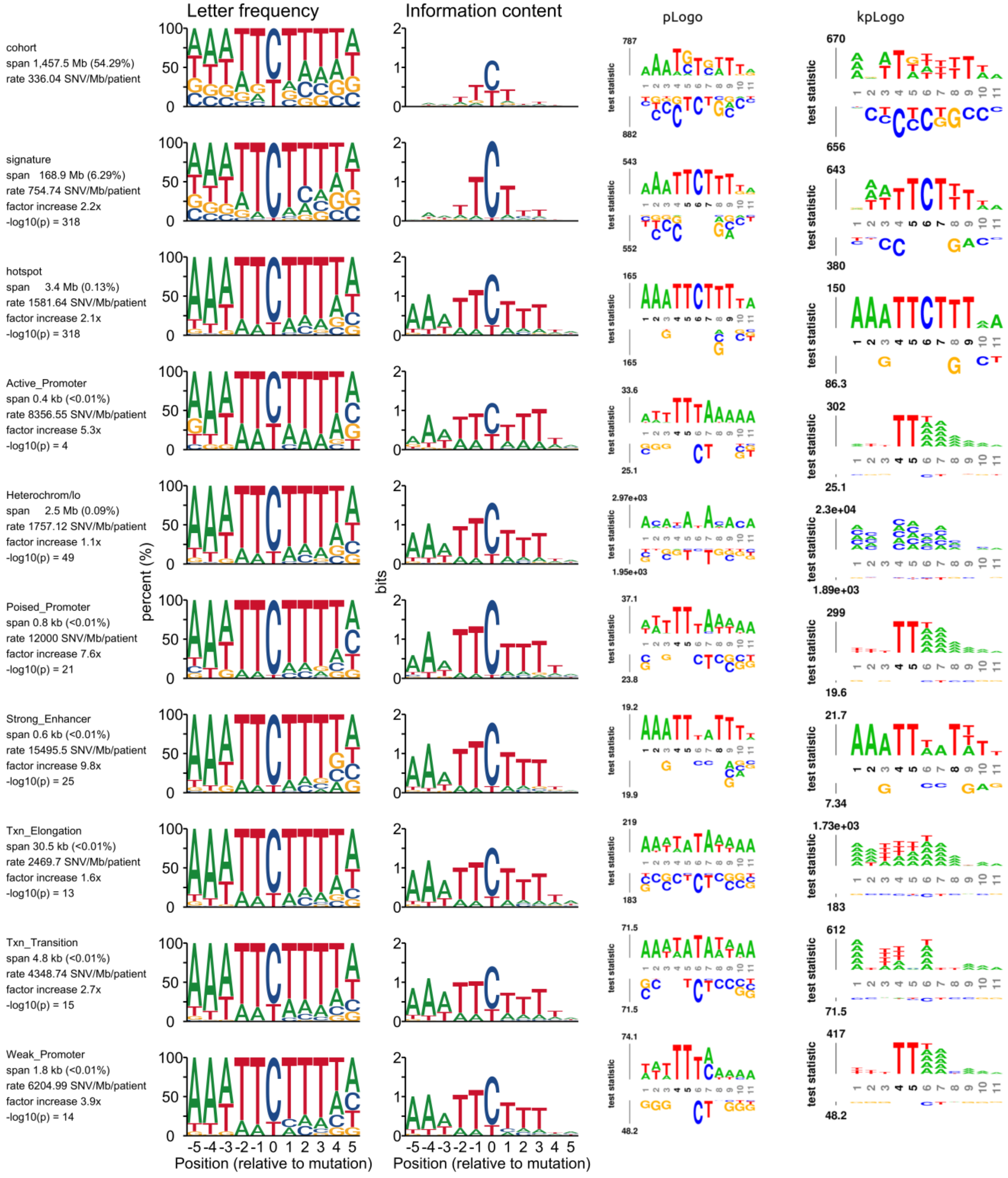

Letter frequency
Information content
pLogo
kpLogo
cohort
span 1,457.5 Mb (54.29%)
rate 336.04 SNV/Mb/patient
signature
span 168.9 Mb (6.29%)
rate 754.74 SNV/Mb/patient
factor increase 2.2x
-log10(p) = 318
hotspot
span 3.4 Mb (0.13%)
rate 1581.64 SNV/Mb/patient
factor increase 2.1x
-log10(p) = 318
Active_Promoter
span 0.4 kb (<0.01%)
rate 8356.55 SNV/Mb/patient
factor increase 5.3x
-log10(p) = 4
Heterochrom/lo
span 2.5 Mb (0.09%)
rate 1757.12 SNV/Mb/patient
factor increase 1.1x
-log10(p) = 49
Poised_Promoter
span 0.8 kb (<0.01%)
rate 12000 SNV/Mb/patient
factor increase 7.6x
-log10(p) = 21
Strong_Enhancer
span 0.6 kb (<0.01%)
rate 15495.5 SNV/Mb/patient
factor increase 9.8x
-log10(p) = 25
Txn_Elongation
span 30.5 kb (<0.01%)
rate 2469.7 SNV/Mb/patient
factor increase 1.6x
-log10(p) = 13
Txn_Transition
span 4.8 kb (<0.01%)
rate 4348.74 SNV/Mb/patient
factor increase 2.7x
-log10(p) = 15
Weak_Promoter
span 1.8 kb (<0.01%)
rate 6204.99 SNV/Mb/patient
factor increase 3.9x
-log10(p) = 14
percent (%)
bits
test statistic
Position (relative to mutation)
Position (relative to mutation)

SBS11

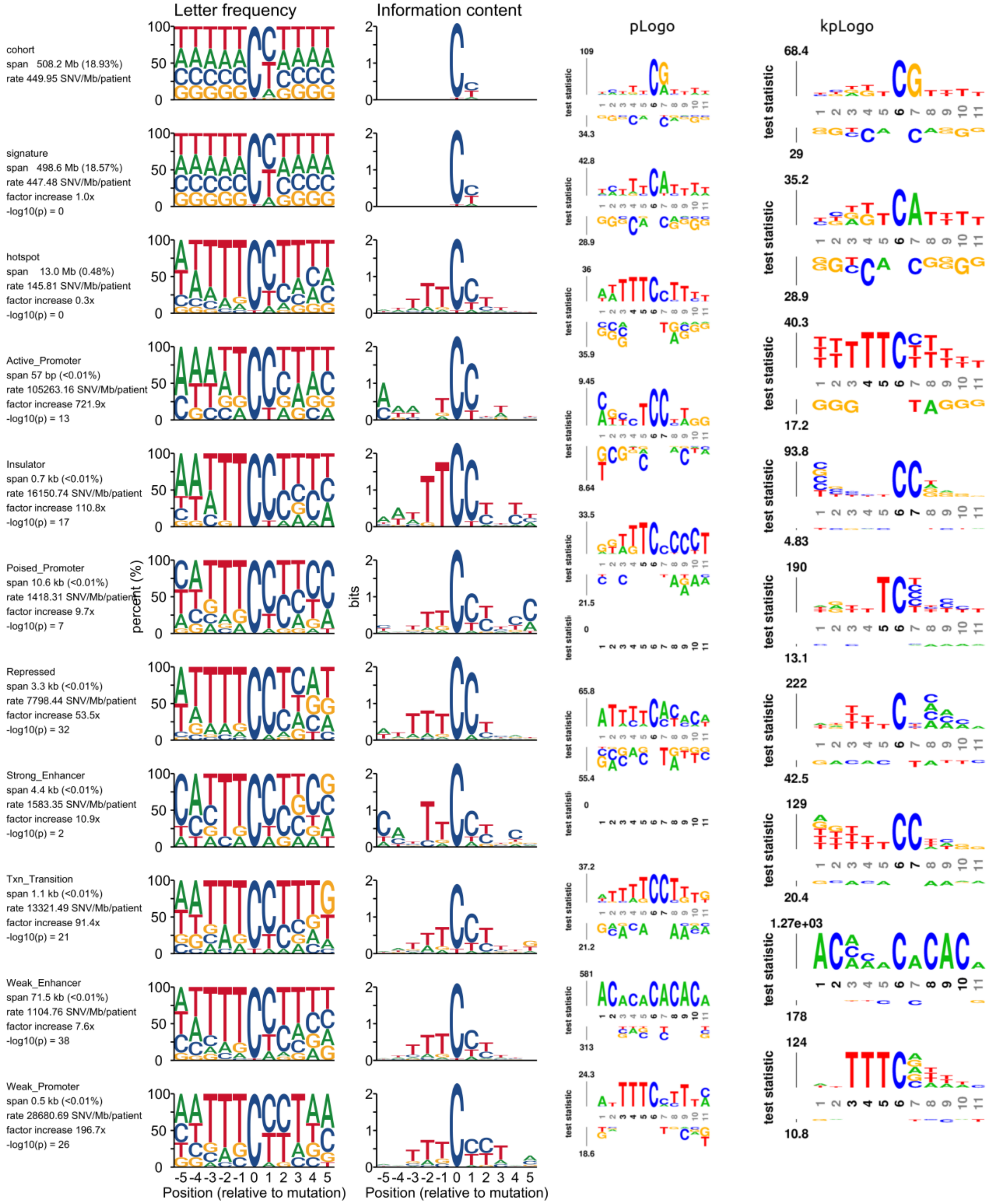
Letter frequency
Information content
pLogo
kpLogo
cohort
span 508.2 Mb (18.93%)
rate 449.95 SNV/Mb/patient
signature
span 498.6 Mb (18.57%)
rate 447.48 SNV/Mb/patient
factor increase 1.0x
-log10(p) = 0
hotspot
span 13.0 Mb (0.48%)
rate 145.81 SNV/Mb/patient
factor increase 0.3x
-log10(p) = 0
Active_Promoter
span 57 bp (<0.01%)
rate 105263.16 SNV/Mb/patient
factor increase 721.9x
-log10(p) = 13
Insulator
span 0.7 kb (<0.01%)
rate 16150.74 SNV/Mb/patient
factor increase 110.8x
-log10(p) = 17
Poised_Promoter
span 10.6 kb (<0.01%)
rate 1418.31 SNV/Mb/patient
factor increase 9.7x
-log10(p) = 7
Repressed
span 3.3 kb (<0.01%)
rate 7798.44 SNV/Mb/patient
factor increase 53.5x
-log10(p) = 32
Strong_Enhancer
span 4.4 kb (<0.01%)
rate 1583.35 SNV/Mb/patient
factor increase 10.9x
-log10(p) = 2
Txn_Transition
span 1.1 kb (<0.01%)
rate 13321.49 SNV/Mb/patient
factor increase 91.4x
-log10(p) = 21
Weak_Enhancer
span 71.5 kb (<0.01%)
rate 1104.76 SNV/Mb/patient
factor increase 7.6x
-log10(p) = 38
Weak_Promoter
span 0.5 kb (<0.01%)
rate 28680.69 SNV/Mb/patient
factor increase 196.7x
-log10(p) = 26
percent (%)
bits
test statistic
Position (relative to mutation)
Position (relative to mutation)

SBS12

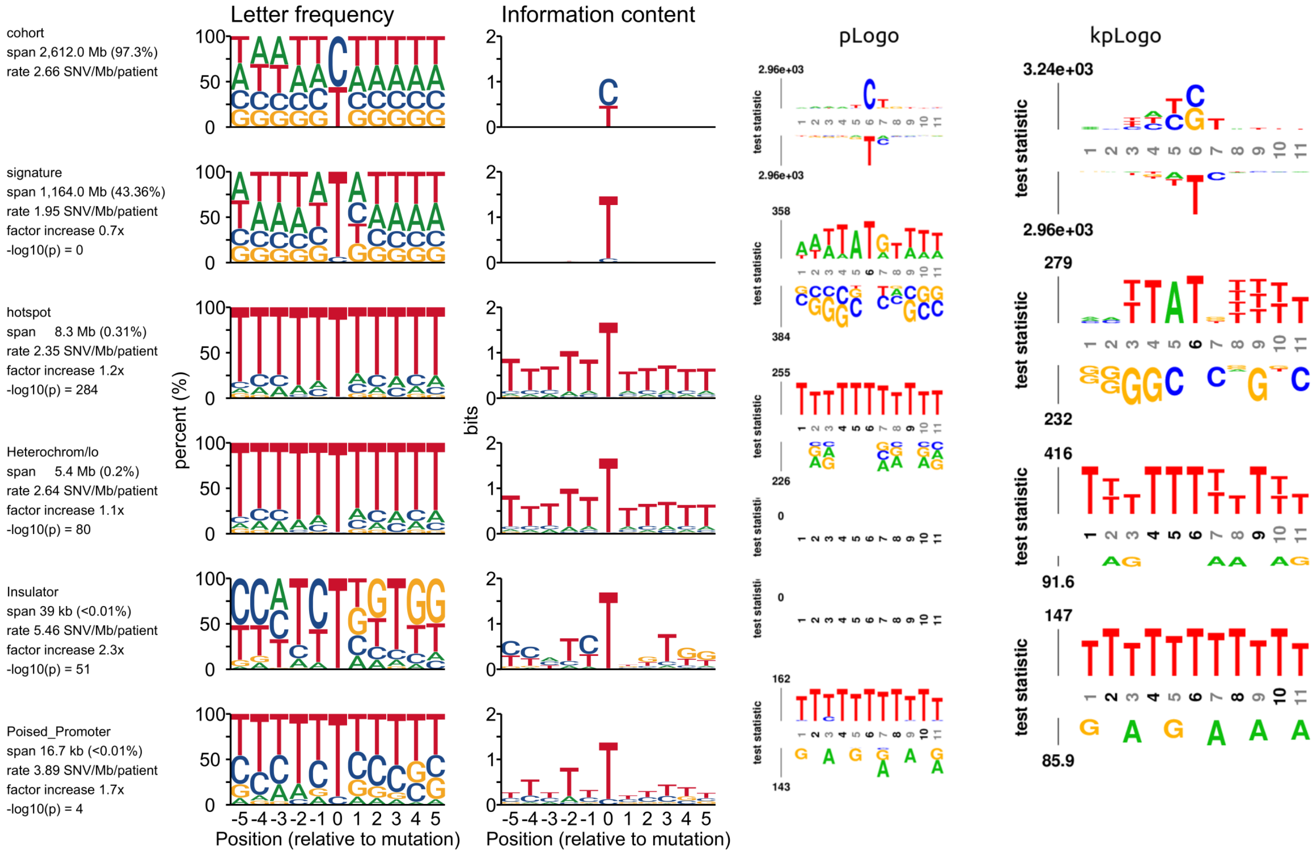

Letter frequency
Information content
pLogo
kpLogo
cohort
span 2,612.0 Mb (97.3%)
rate 2.66 SNV/Mb/patient
signature
span 1,164.0 Mb (43.36%)
rate 1.95 SNV/Mb/patient
factor increase 0.7x
-log10(p) = 0
hotspot
span 8.3 Mb (0.31%)
rate 2.35 SNV/Mb/patient
factor increase 1.2x
-log10(p) = 284
Heterochrom/lo
span 5.4 Mb (0.2%)
rate 2.64 SNV/Mb/patient
factor increase 1.1x
-log10(p) = 80
Insulator
span 39 kb (<0.01%)
rate 5.46 SNV/Mb/patient
factor increase 2.3x
-log10(p) = 51
Poised_Promoter
span 16.7 kb (<0.01%)
rate 3.89 SNV/Mb/patient
factor increase 1.7x
-log10(p) = 4
percent (%)
bits
Position (relative to mutation)
Position (relative to mutation)
test statistic
2.96e+03
2.96e+03
358
384
255
226
0
0
162
143
3.24e+03
2.96e+03
279
232
416
91.6
147
85.9

SBS13

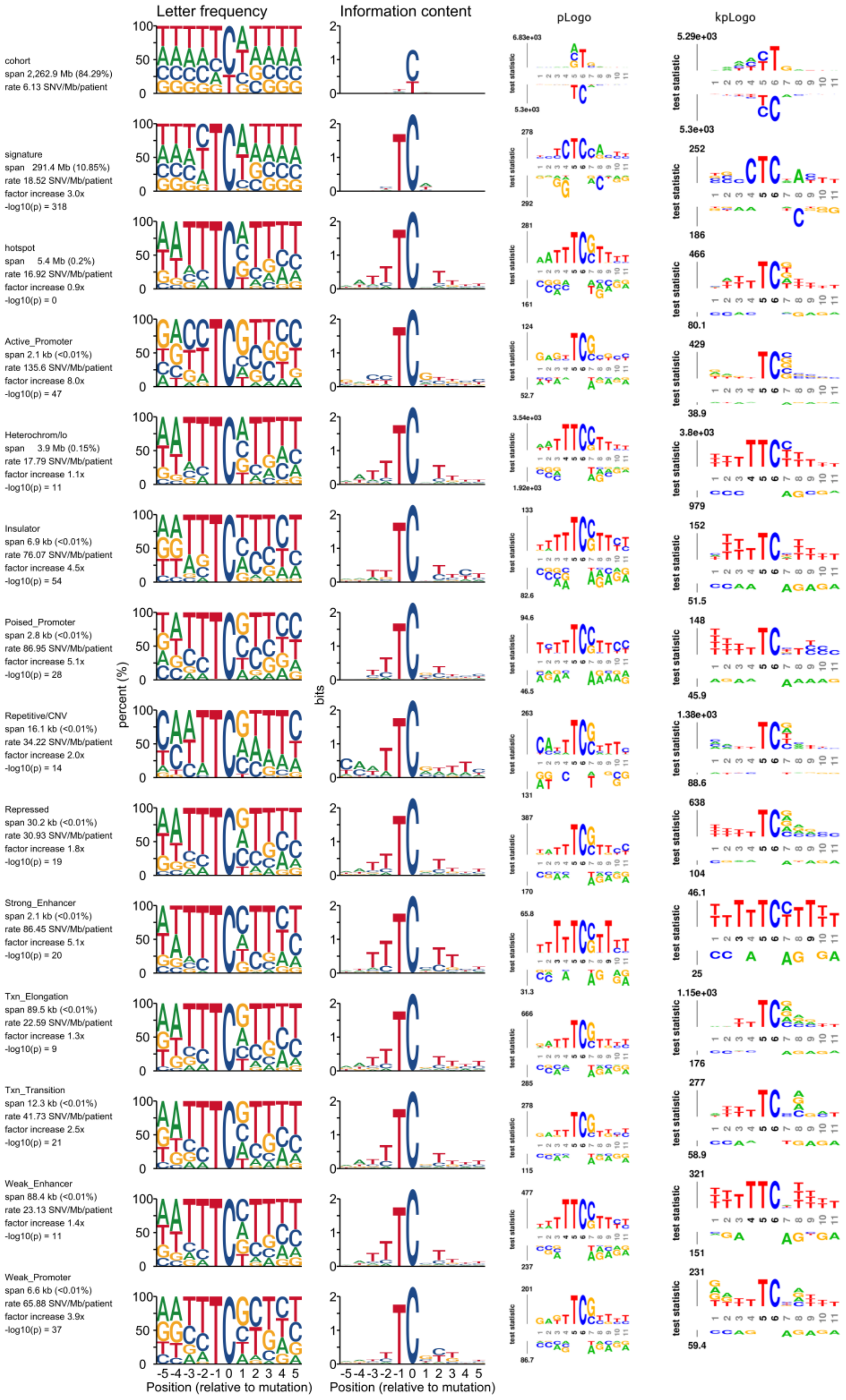
Letter frequency
Information content
pLogo
kpLogo
cohort
span 2,262.9 Mb (84.29%)
rate 6.13 SNV/Mb/patient
signature
span 291.4 Mb (10.85%)
rate 18.52 SNV/Mb/patient
factor increase 3.0x
-log10(p) = 318
hotspot
span 5.4 Mb (0.2%)
rate 16.92 SNV/Mb/patient
factor increase 0.9x
-log10(p) = 0
Active_Promoter
span 2.1 kb (<0.01%)
rate 135.6 SNV/Mb/patient
factor increase 8.0x
-log10(p) = 47
Heterochrom/lo
span 3.9 Mb (0.15%)
rate 17.79 SNV/Mb/patient
factor increase 1.1x
-log10(p) = 11
Insulator
span 6.9 kb (<0.01%)
rate 76.07 SNV/Mb/patient
factor increase 4.5x
-log10(p) = 54
Poised_Promoter
span 2.8 kb (<0.01%)
rate 86.95 SNV/Mb/patient
factor increase 5.1x
-log10(p) = 28
Repetitive/CNV
span 16.1 kb (<0.01%)
rate 34.22 SNV/Mb/patient
factor increase 2.0x
-log10(p) = 14
Repressed
span 30.2 kb (<0.01%)
rate 30.93 SNV/Mb/patient
factor increase 1.8x
-log10(p) = 19
Strong_Enhancer
span 2.1 kb (<0.01%)
rate 86.45 SNV/Mb/patient
factor increase 5.1x
-log10(p) = 20
Txn_Elongation
span 89.5 kb (<0.01%)
rate 22.59 SNV/Mb/patient
factor increase 1.3x
-log10(p) = 9
Txn_Transition
span 12.3 kb (<0.01%)
rate 41.73 SNV/Mb/patient
factor increase 2.5x
-log10(p) = 21
Weak_Enhancer
span 88.4 kb (<0.01%)
rate 23.13 SNV/Mb/patient
factor increase 1.4x
-log10(p) = 11
Weak_Promoter
span 6.6 kb (<0.01%)
rate 65.88 SNV/Mb/patient
factor increase 3.9x
-log10(p) = 37
percent (%)
bits
test statistic
Position (relative to mutation)
Position (relative to mutation)

SBS16

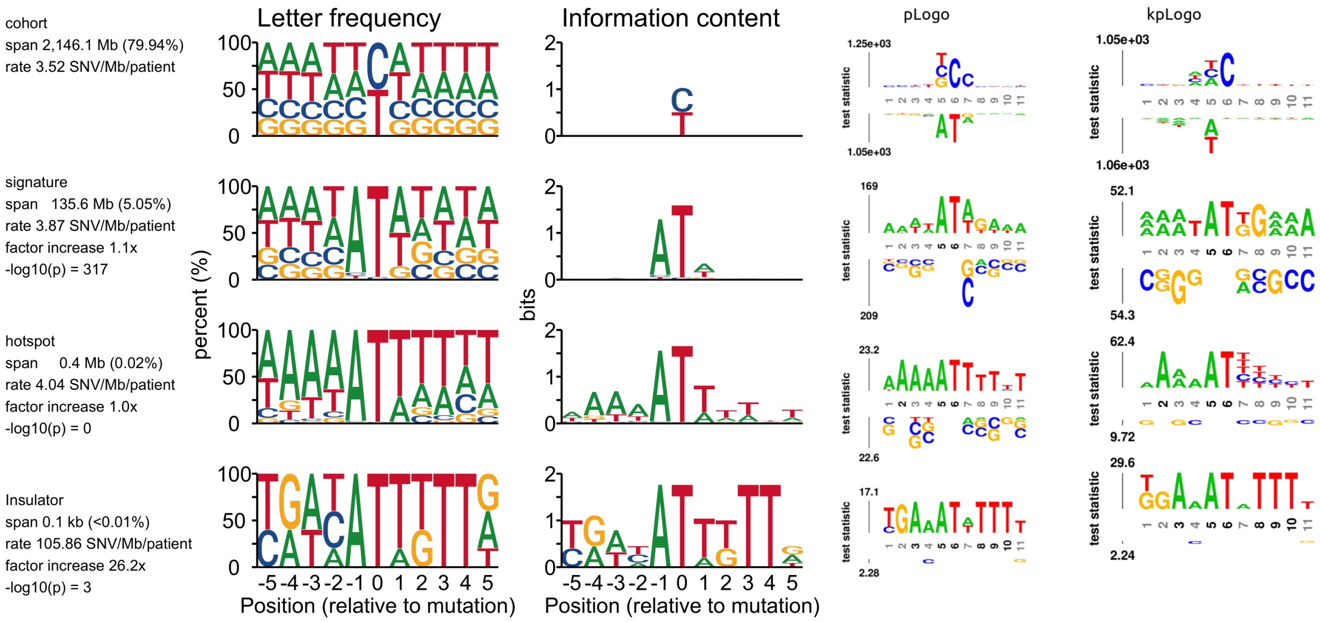
Letter frequency
Information content
pLogo
kpLogo
cohort
span 2,146.1 Mb (79.94%)
rate 3.52 SNV/Mb/patient
signature
span 135.6 Mb (5.05%)
rate 3.87 SNV/Mb/patient
factor increase 1.1x
-log10(p) = 317
hotspot
span 0.4 Mb (0.02%)
rate 4.04 SNV/Mb/patient
factor increase 1.0x
-log10(p) = 0
Insulator
span 0.1 kb (<0.01%)
rate 105.86 SNV/Mb/patient
factor increase 26.2x
-log10(p) = 3
percent (%)
bits
test statistic
Position (relative to mutation)
Position (relative to mutation)
1.25e+03
1.05e+03
1.05e+03
1.06e+03
169
209
52.1
54.3
23.2
22.6
62.4
9.72
17.1
2.28
29.6
2.24

SBS17b

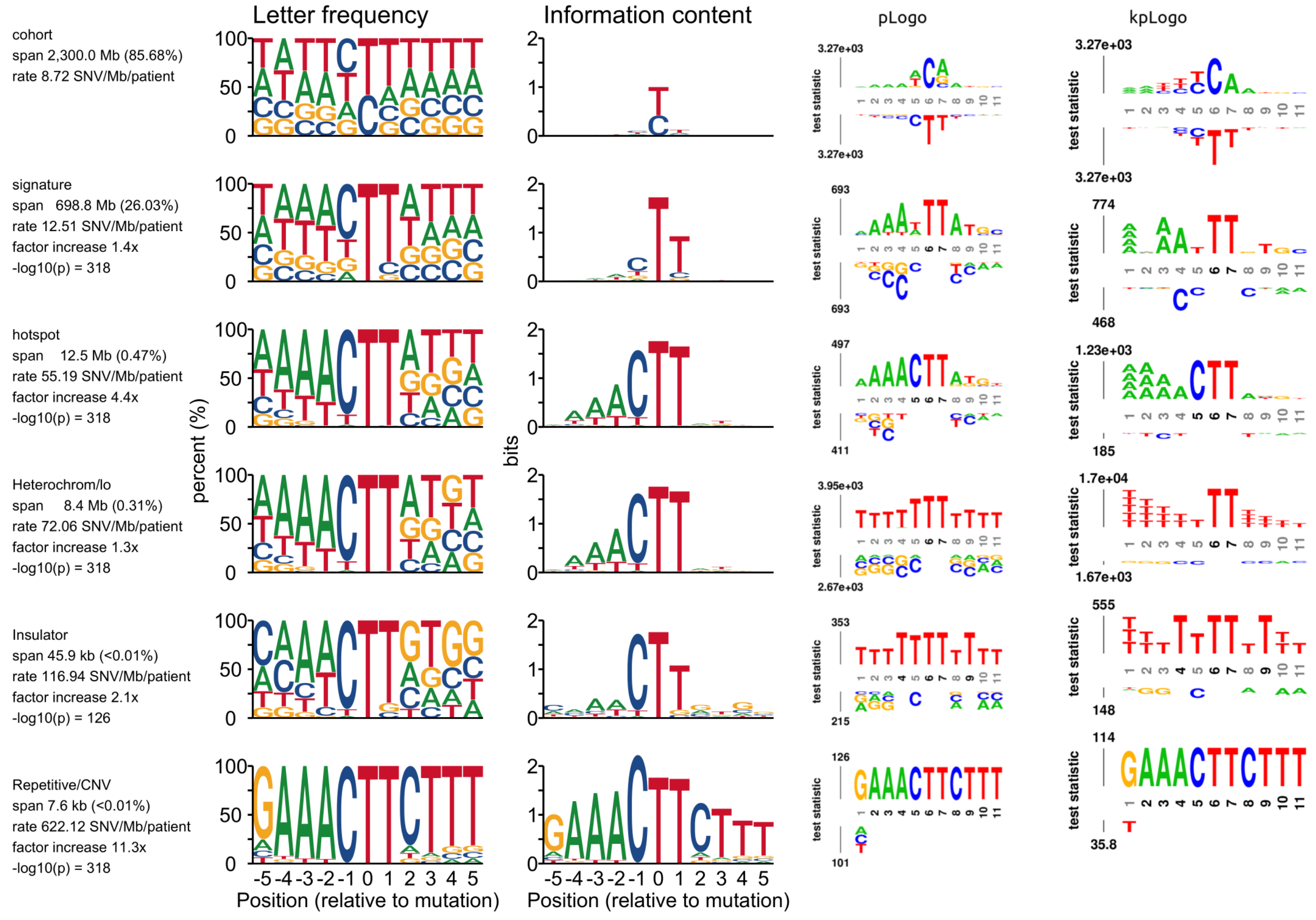
Letter frequency
Information content
pLogo
kpLogo
cohort
span 2,300.0 Mb (85.68%)
rate 8.72 SNV/Mb/patient
signature
span 698.8 Mb (26.03%)
rate 12.51 SNV/Mb/patient
factor increase 1.4x
-log10(p) = 318
hotspot
span 12.5 Mb (0.47%)
rate 55.19 SNV/Mb/patient
factor increase 4.4x
-log10(p) = 318
Heterochrom/lo
span 8.4 Mb (0.31%)
rate 72.06 SNV/Mb/patient
factor increase 1.3x
-log10(p) = 318
Insulator
span 45.9 kb (<0.01%)
rate 116.94 SNV/Mb/patient
factor increase 2.1x
-log10(p) = 126
Repetitive/CNV
span 7.6 kb (<0.01%)
rate 622.12 SNV/Mb/patient
factor increase 11.3x
-log10(p) = 318
percent (%)
bits
test statistic
Position (relative to mutation)
Position (relative to mutation)

SBS18

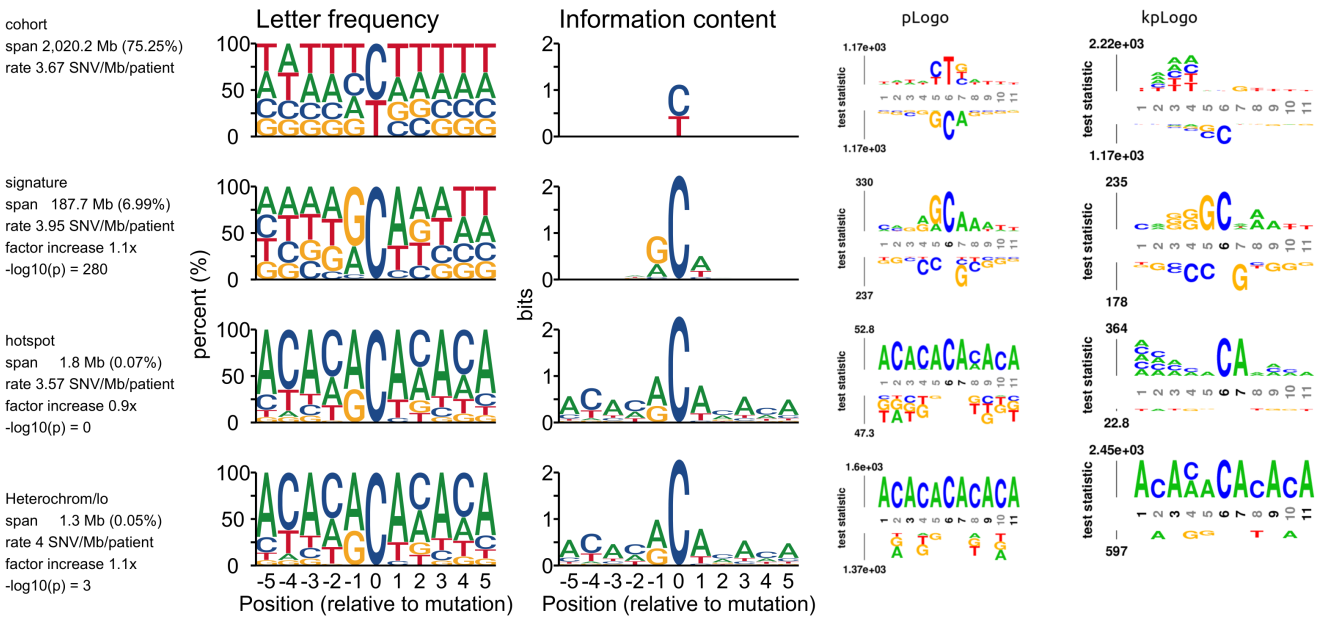

cohort
span 2,020.2 Mb (75.25%)
rate 3.67 SNV/Mb/patient
signature
span 187.7 Mb (6.99%)
rate 3.95 SNV/Mb/patient
factor increase 1.1x
-log10(p) = 280
hotspot
span 1.8 Mb (0.07%)
rate 3.57 SNV/Mb/patient
factor increase 0.9x
-log10(p) = 0
Heterochrom/lo
span 1.3 Mb (0.05%)
rate 4 SNV/Mb/patient
factor increase 1.1x
-log10(p) = 3
Letter frequency
Information content
pLogo
kpLogo
percent (%)
bits
test statistic
Position (relative to mutation)
Position (relative to mutation)

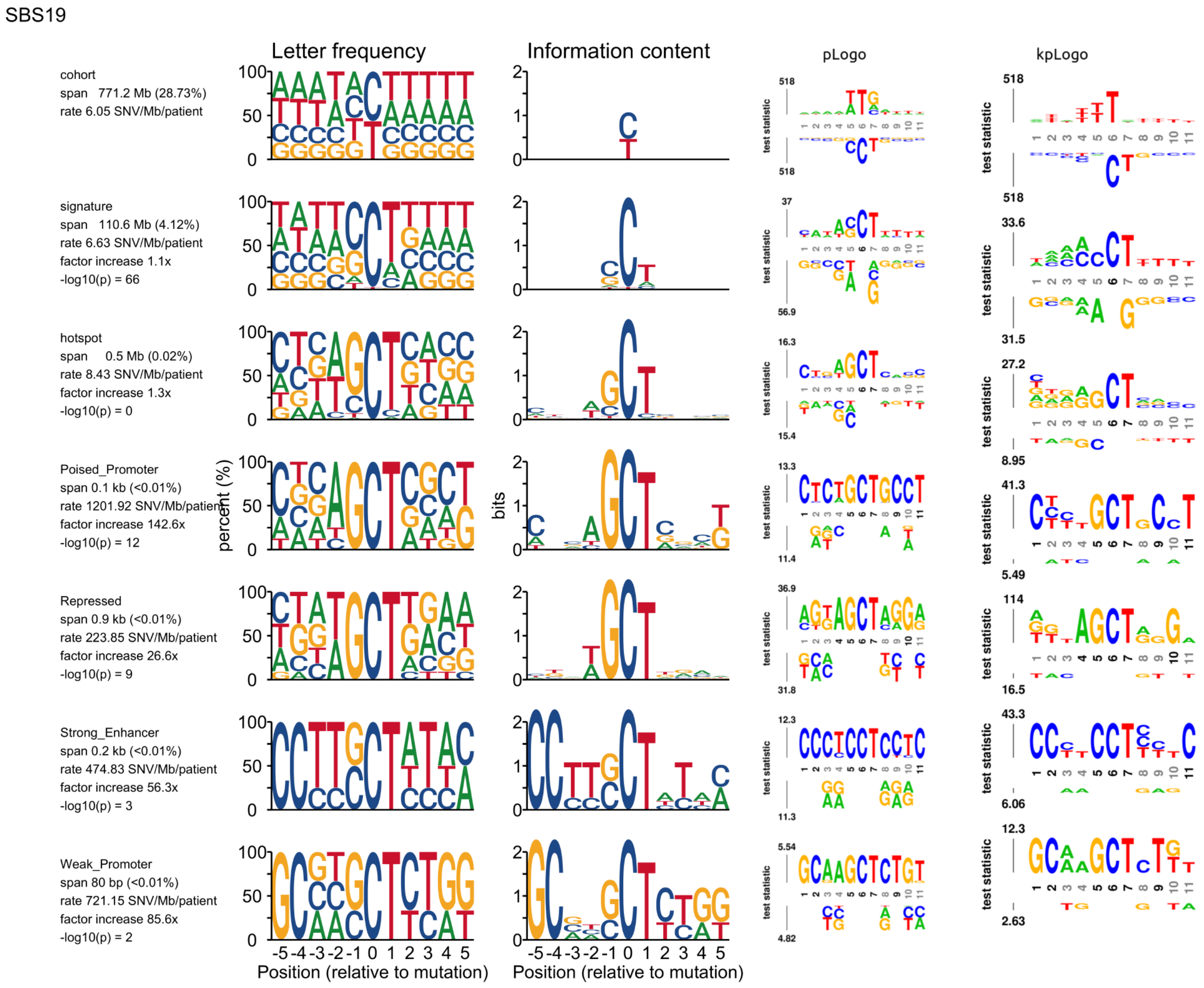
SBS19
Letter frequency
Information content
pLogo
kpLogo
cohort
span 771.2 Mb (28.73%)
rate 6.05 SNV/Mb/patient
signature
span 110.6 Mb (4.12%)
rate 6.63 SNV/Mb/patient
factor increase 1.1x
-log10(p) = 66
hotspot
span 0.5 Mb (0.02%)
rate 8.43 SNV/Mb/patient
factor increase 1.3x
-log10(p) = 0
Poised_Promoter
span 0.1 kb (<0.01%)
rate 1201.92 SNV/Mb/patient
factor increase 142.6x
-log10(p) = 12
Repressed
span 0.9 kb (<0.01%)
rate 223.85 SNV/Mb/patient
factor increase 26.6x
-log10(p) = 9
Strong_Enhancer
span 0.2 kb (<0.01%)
rate 474.83 SNV/Mb/patient
factor increase 56.3x
-log10(p) = 3
Weak_Promoter
span 80 bp (<0.01%)
rate 721.15 SNV/Mb/patient
factor increase 85.6x
-log10(p) = 2
percent (%)
bits
test statistic
Position (relative to mutation)
Position (relative to mutation)

SBS22

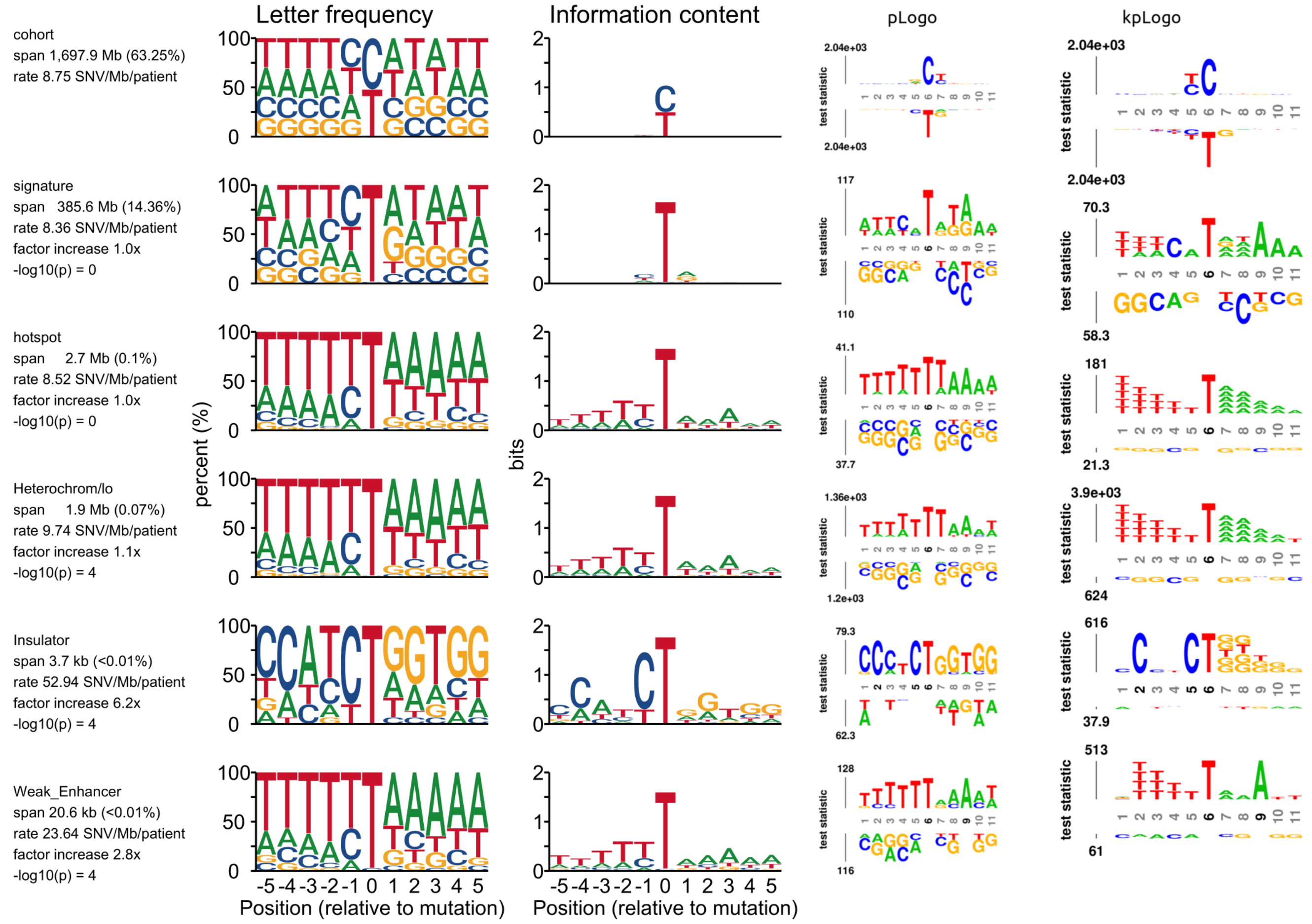

Letter frequency
Information content
pLogo
kpLogo
cohort
span 1,697.9 Mb (63.25%)
rate 8.75 SNV/Mb/patient
signature
span 385.6 Mb (14.36%)
rate 8.36 SNV/Mb/patient
factor increase 1.0x
-log10(p) = 0
hotspot
span 2.7 Mb (0.1%)
rate 8.52 SNV/Mb/patient
factor increase 1.0x
-log10(p) = 0
Heterochrom/lo
span 1.9 Mb (0.07%)
rate 9.74 SNV/Mb/patient
factor increase 1.1x
-log10(p) = 4
Insulator
span 3.7 kb (<0.01%)
rate 52.94 SNV/Mb/patient
factor increase 6.2x
-log10(p) = 4
Weak_Enhancer
span 20.6 kb (<0.01%)
rate 23.64 SNV/Mb/patient
factor increase 2.8x
-log10(p) = 4
percent (%)
bits
test statistic
Position (relative to mutation)
Position (relative to mutation)

SBS26

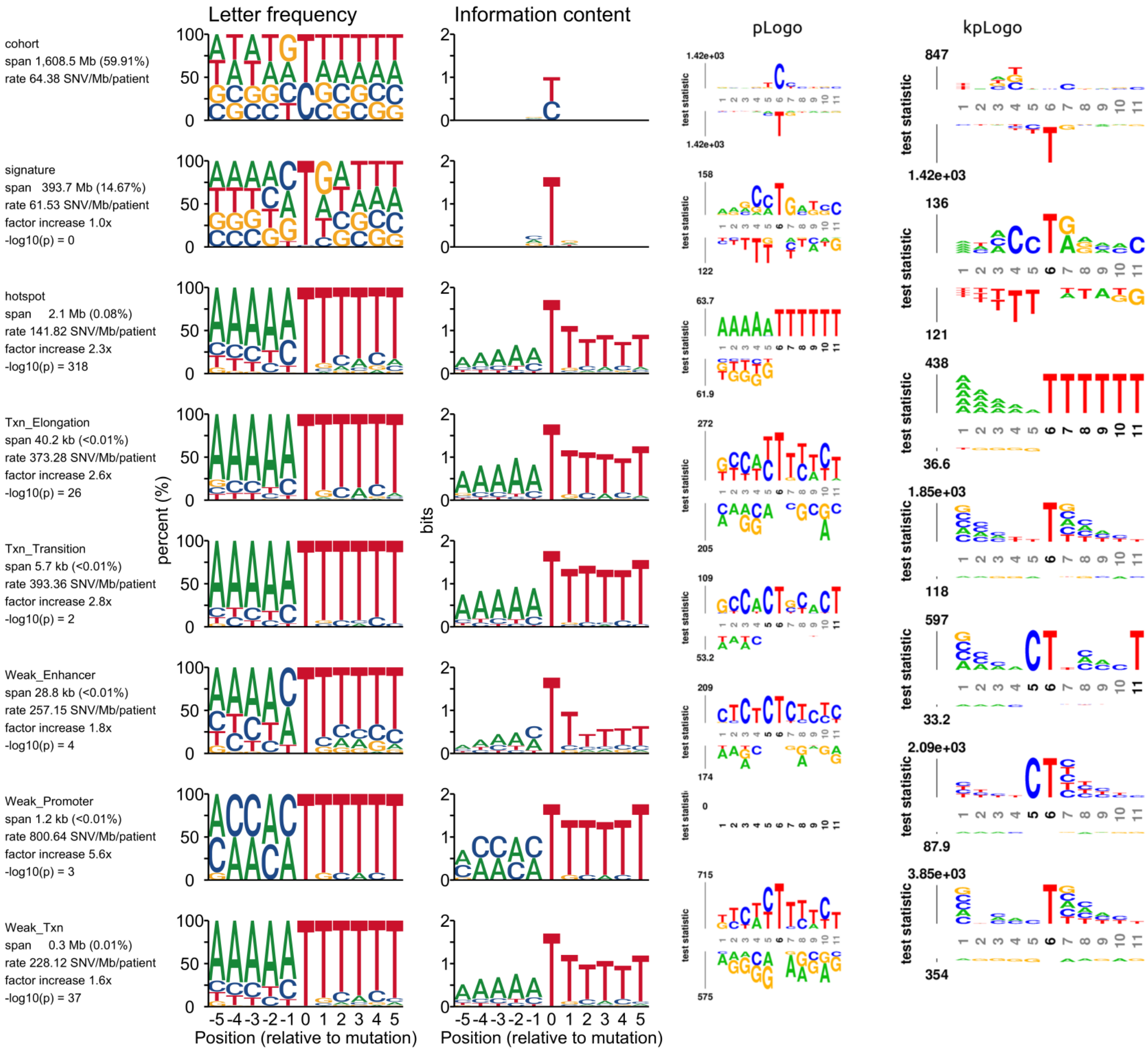
Letter frequency
Information content
pLogo
kpLogo
cohort
span 1,608.5 Mb (59.91%)
rate 64.38 SNV/Mb/patient
signature
span 393.7 Mb (14.67%)
rate 61.53 SNV/Mb/patient
factor increase 1.0x
-log10(p) = 0
hotspot
span 2.1 Mb (0.08%)
rate 141.82 SNV/Mb/patient
factor increase 2.3x
-log10(p) = 318
Txn_Elongation
span 40.2 kb (<0.01%)
rate 373.28 SNV/Mb/patient
factor increase 2.6x
-log10(p) = 26
Txn_Transition
span 5.7 kb (<0.01%)
rate 393.36 SNV/Mb/patient
factor increase 2.8x
-log10(p) = 2
Weak_Enhancer
span 28.8 kb (<0.01%)
rate 257.15 SNV/Mb/patient
factor increase 1.8x
-log10(p) = 4
Weak_Promoter
span 1.2 kb (<0.01%)
rate 800.64 SNV/Mb/patient
factor increase 5.6x
-log10(p) = 3
Weak_Txn
span 0.3 Mb (0.01%)
rate 228.12 SNV/Mb/patient
factor increase 1.6x
-log10(p) = 37
percent (%)
bits
Position (relative to mutation)
Position (relative to mutation)
test statistic

SBS30

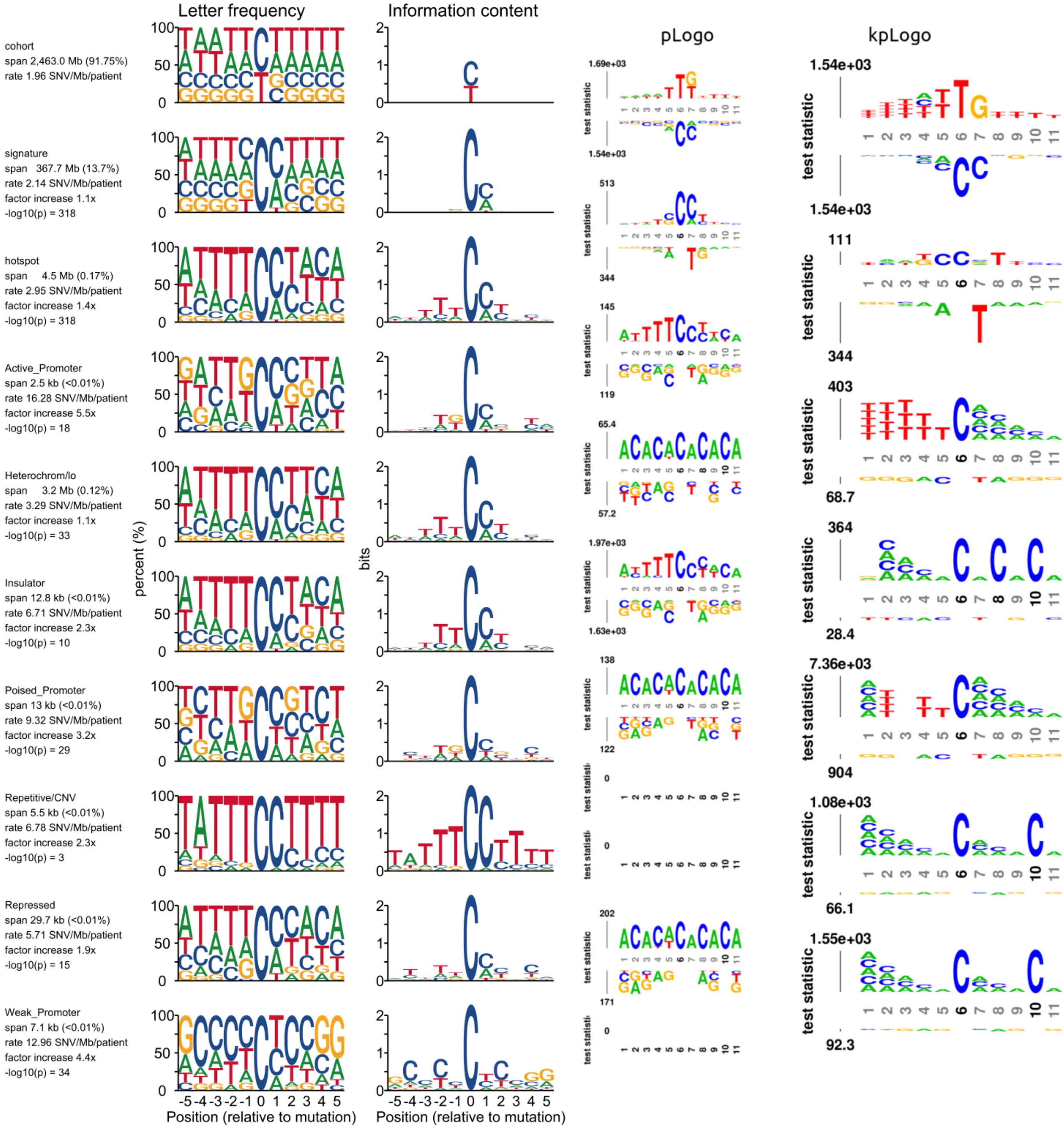
Letter frequency
Information content
pLogo
kpLogo
cohort
span 2,463.0 Mb (91.75%)
rate 1.96 SNV/Mb/patient
signature
span 367.7 Mb (13.7%)
rate 2.14 SNV/Mb/patient
factor increase 1.1x
-log10(p) = 318
hotspot
span 4.5 Mb (0.17%)
rate 2.95 SNV/Mb/patient
factor increase 1.4x
-log10(p) = 318
Active_Promoter
span 2.5 kb (<0.01%)
rate 16.28 SNV/Mb/patient
factor increase 5.5x
-log10(p) = 18
Heterochrom/lo
span 3.2 Mb (0.12%)
rate 3.29 SNV/Mb/patient
factor increase 1.1x
-log10(p) = 33
Insulator
span 12.8 kb (<0.01%)
rate 6.71 SNV/Mb/patient
factor increase 2.3x
-log10(p) = 10
Poised_Promoter
span 13 kb (<0.01%)
rate 9.32 SNV/Mb/patient
factor increase 3.2x
-log10(p) = 29
Repetitive/CNV
span 5.5 kb (<0.01%)
rate 6.78 SNV/Mb/patient
factor increase 2.3x
-log10(p) = 3
Repressed
span 29.7 kb (<0.01%)
rate 5.71 SNV/Mb/patient
factor increase 1.9x
-log10(p) = 15
Weak_Promoter
span 7.1 kb (<0.01%)
rate 12.96 SNV/Mb/patient
factor increase 4.4x
-log10(p) = 34
percent (%)
bits
test statistic
Position (relative to mutation)
Position (relative to mutation)

SBS35

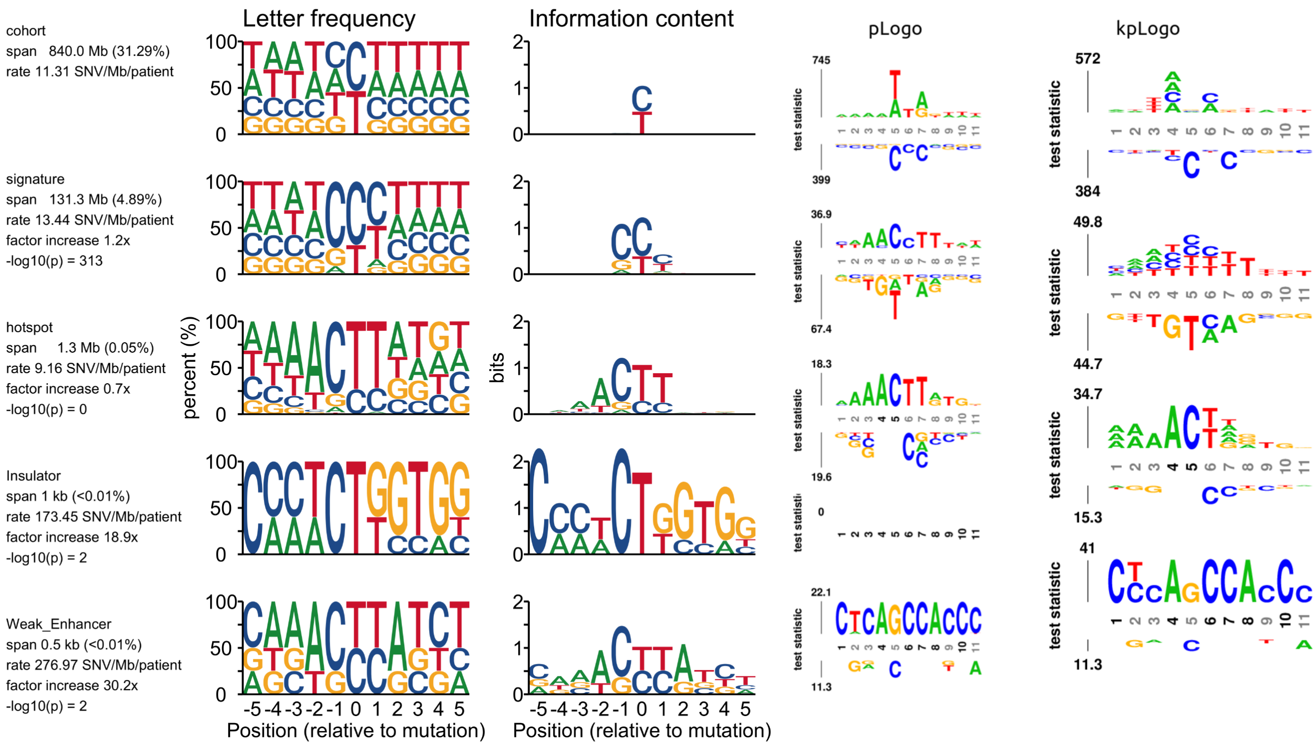

cohort
span 840.0 Mb (31.29%)
rate 11.31 SNV/Mb/patient
signature
span 131.3 Mb (4.89%)
rate 13.44 SNV/Mb/patient
factor increase 1.2x
-log10(p) = 313
hotspot
span 1.3 Mb (0.05%)
rate 9.16 SNV/Mb/patient
factor increase 0.7x
-log10(p) = 0
Insulator
span 1 kb (<0.01%)
rate 173.45 SNV/Mb/patient
factor increase 18.9x
-log10(p) = 2
Weak_Enhancer
span 0.5 kb (<0.01%)
rate 276.97 SNV/Mb/patient
factor increase 30.2x
-log10(p) = 2
Letter frequency
Information content
pLogo
kpLogo
percent (%)
bits
test statistic
Position (relative to mutation)

SBS36

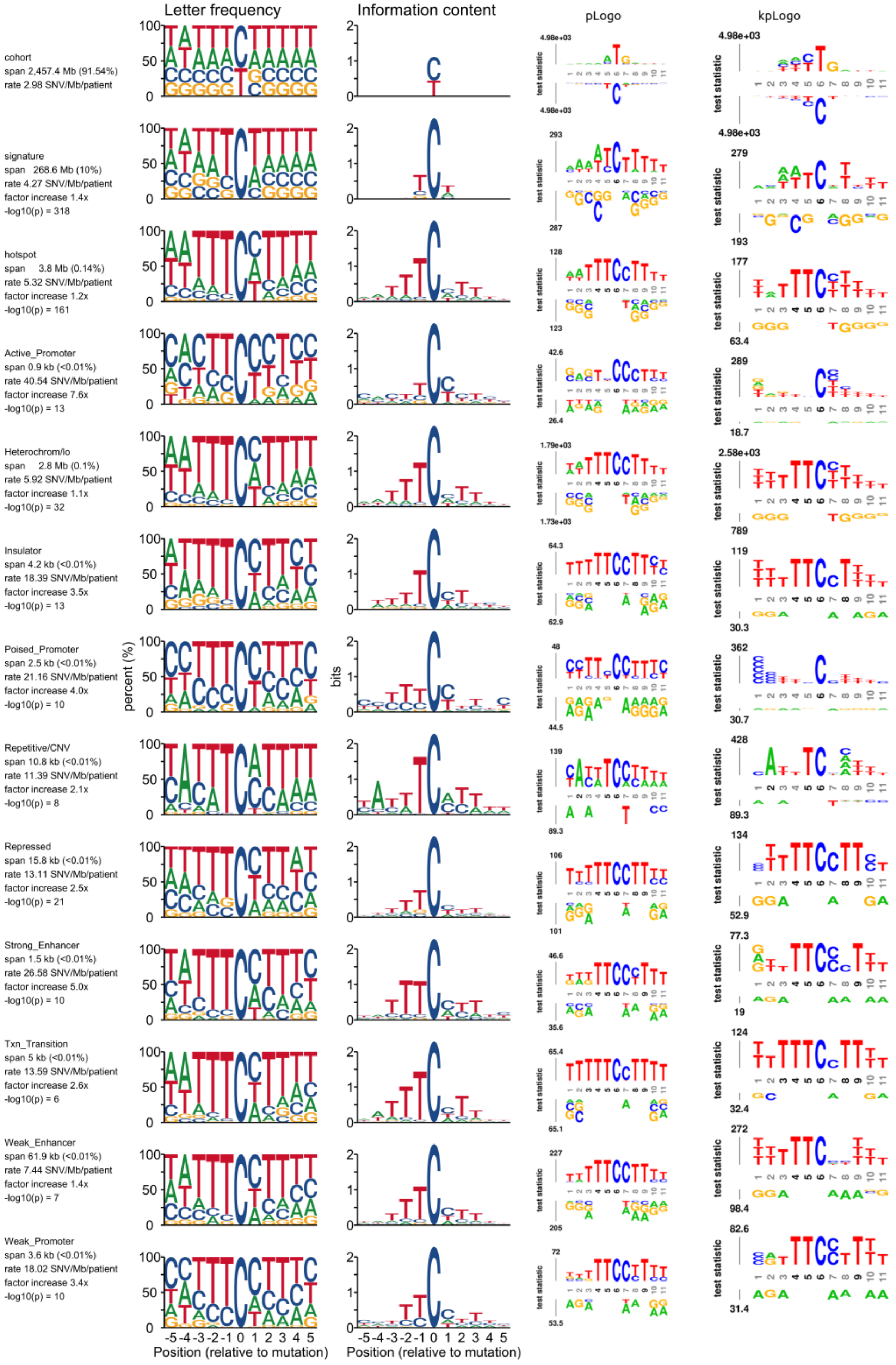
Letter frequency
Information content
pLogo
kpLogo
cohort
span 2,457.4 Mb (91.54%)
rate 2.98 SNV/Mb/patient
signature
span 268.6 Mb (10%)
rate 4.27 SNV/Mb/patient
factor increase 1.4x
-log10(p) = 318
hotspot
span 3.8 Mb (0.14%)
rate 5.32 SNV/Mb/patient
factor increase 1.2x
-log10(p) = 161
Active_Promoter
span 0.9 kb (<0.01%)
rate 40.54 SNV/Mb/patient
factor increase 7.6x
-log10(p) = 13
Heterochrom/lo
span 2.8 Mb (0.1%)
rate 5.92 SNV/Mb/patient
factor increase 1.1x
-log10(p) = 32
Insulator
span 4.2 kb (<0.01%)
rate 18.39 SNV/Mb/patient
factor increase 3.5x
-log10(p) = 13
Poised_Promoter
span 2.5 kb (<0.01%)
rate 21.16 SNV/Mb/patient
factor increase 4.0x
-log10(p) = 10
Repetitive/CNV
span 10.8 kb (<0.01%)
rate 11.39 SNV/Mb/patient
factor increase 2.1x
-log10(p) = 8
Repressed
span 15.8 kb (<0.01%)
rate 13.11 SNV/Mb/patient
factor increase 2.5x
-log10(p) = 21
Strong_Enhancer
span 1.5 kb (<0.01%)
rate 26.58 SNV/Mb/patient
factor increase 5.0x
-log10(p) = 10
Txn_Transition
span 5 kb (<0.01%)
rate 13.59 SNV/Mb/patient
factor increase 2.6x
-log10(p) = 6
Weak_Enhancer
span 61.9 kb (<0.01%)
rate 7.44 SNV/Mb/patient
factor increase 1.4x
-log10(p) = 7
Weak_Promoter
span 3.6 kb (<0.01%)
rate 18.02 SNV/Mb/patient
factor increase 3.4x
-log10(p) = 10
percent (%)
bits
test statistic
-5 -4 -3 -2 -1 0 1 2 3 4 5
Position (relative to mutation)
Position (relative to mutation)

SBS37

cohort
span 434.6 Mb (16.19%)
rate 7.58 SNV/Mb/patient

signature
span 130.6 Mb (4.86%)
rate 8.35 SNV/Mb/patient
factor increase 1.1x
-log10(p) = 80

hotspot
span 8.9 Mb (0.33%)
rate 3.97 SNV/Mb/patient
factor increase 0.5x
-log10(p) = 0

Heterochrom/lo
span 5.8 Mb (0.22%)
rate 5.26 SNV/Mb/patient
factor increase 1.3x
-log10(p) = 17

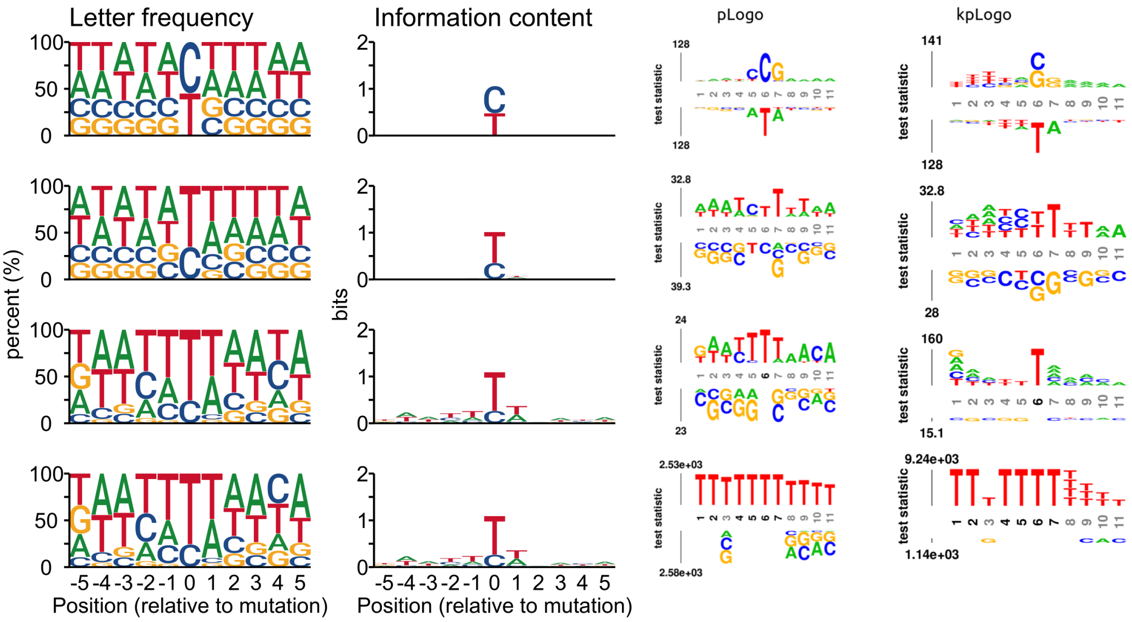

SBS39

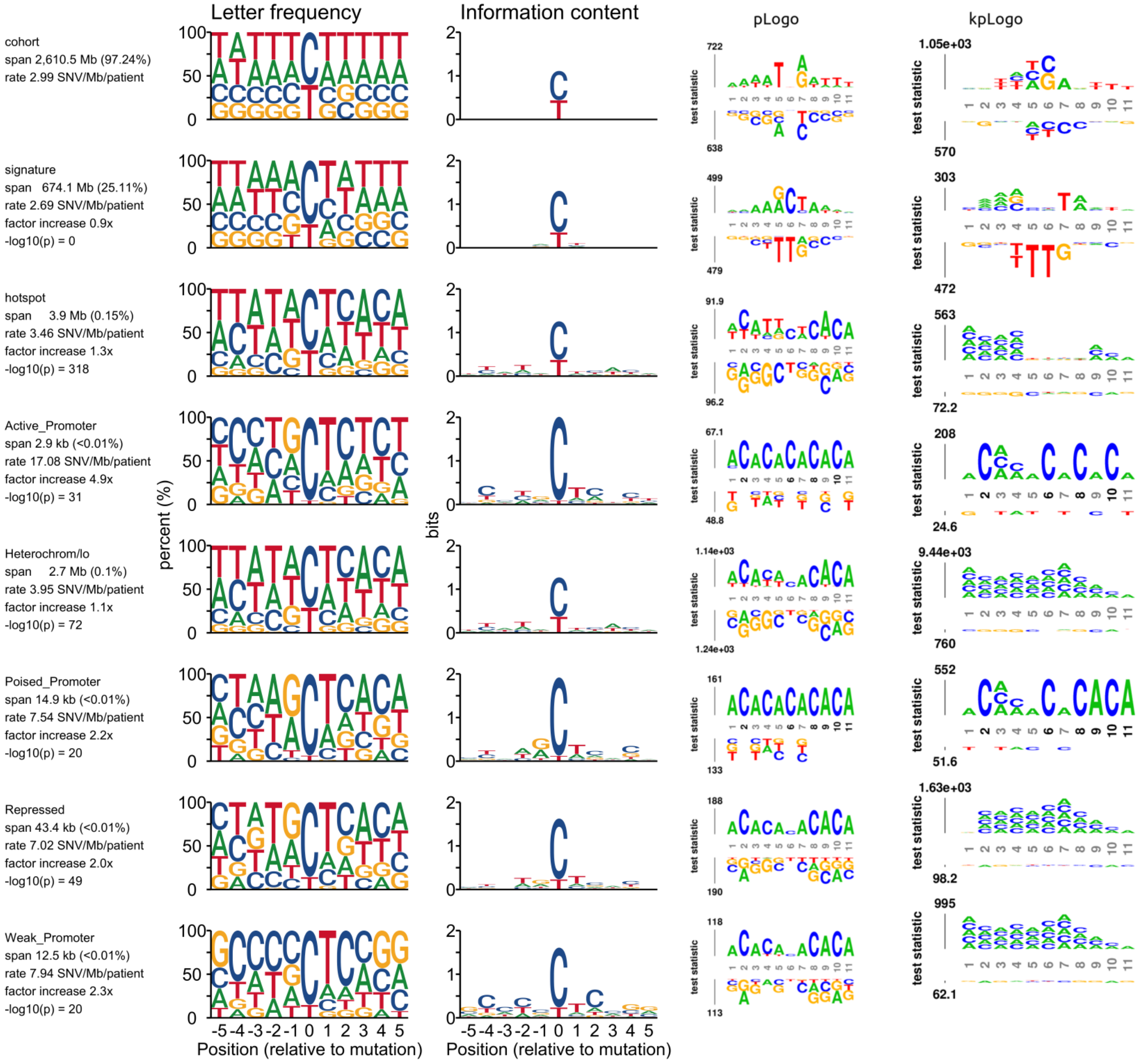
Letter frequency
Information content
pLogo
kpLogo
cohort
span 2,610.5 Mb (97.24%)
rate 2.99 SNV/Mb/patient
signature
span 674.1 Mb (25.11%)
rate 2.69 SNV/Mb/patient
factor increase 0.9x
-log10(p) = 0
hotspot
span 3.9 Mb (0.15%)
rate 3.46 SNV/Mb/patient
factor increase 1.3x
-log10(p) = 318
Active_Promoter
span 2.9 kb (<0.01%)
rate 17.08 SNV/Mb/patient
factor increase 4.9x
-log10(p) = 31
Heterochrom/lo
span 2.7 Mb (0.1%)
rate 3.95 SNV/Mb/patient
factor increase 1.1x
-log10(p) = 72
Poised_Promoter
span 14.9 kb (<0.01%)
rate 7.54 SNV/Mb/patient
factor increase 2.2x
-log10(p) = 20
Repressed
span 43.4 kb (<0.01%)
rate 7.02 SNV/Mb/patient
factor increase 2.0x
-log10(p) = 49
Weak_Promoter
span 12.5 kb (<0.01%)
rate 7.94 SNV/Mb/patient
factor increase 2.3x
-log10(p) = 20
percent (%)
bits
test statistic
Position (relative to mutation)
Position (relative to mutation)

SBS40

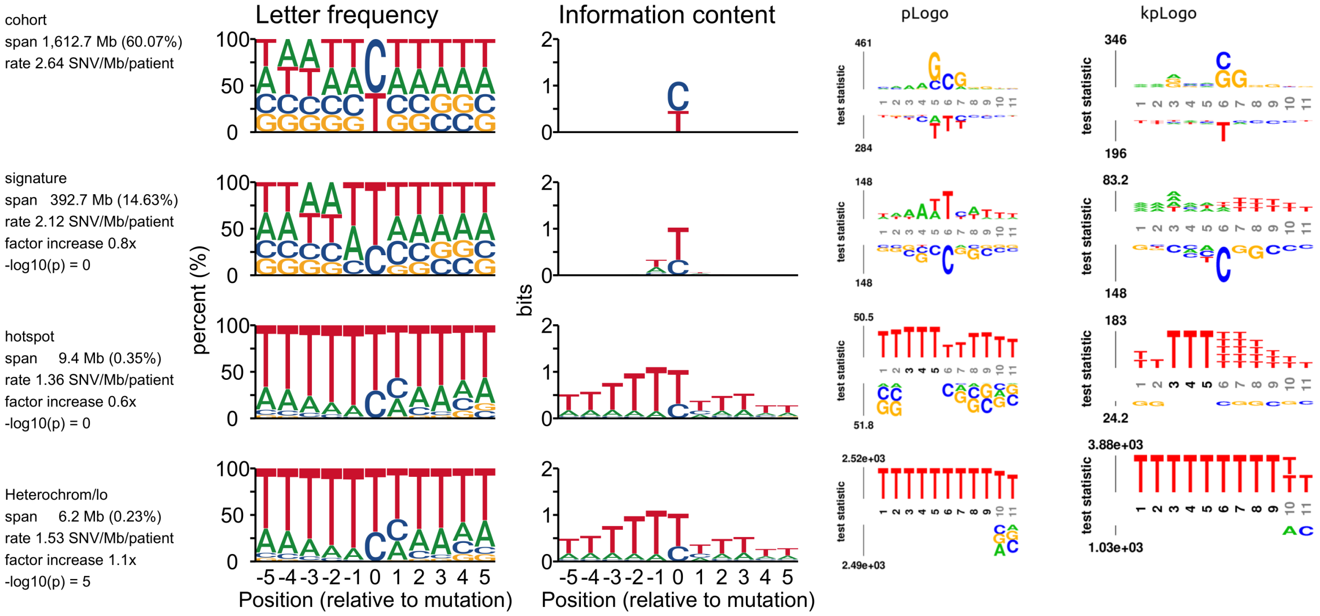

cohort
span 1,612.7 Mb (60.07%)
rate 2.64 SNV/Mb/patient
signature
span 392.7 Mb (14.63%)
rate 2.12 SNV/Mb/patient
factor increase 0.8x
-log10(p) = 0
hotspot
span 9.4 Mb (0.35%)
rate 1.36 SNV/Mb/patient
factor increase 0.6x
-log10(p) = 0
Heterochrom/lo
span 6.2 Mb (0.23%)
rate 1.53 SNV/Mb/patient
factor increase 1.1x
-log10(p) = 5
Letter frequency
percent (%)
Position (relative to mutation)
Information content
bits
Position (relative to mutation)
pLogo
test statistic
461
284
148
148
50.5
51.8
2.52e+03
2.49e+03
kpLogo
346
196
83.2
148
183
24.2
3.88e+03
1.03e+03

SBS44

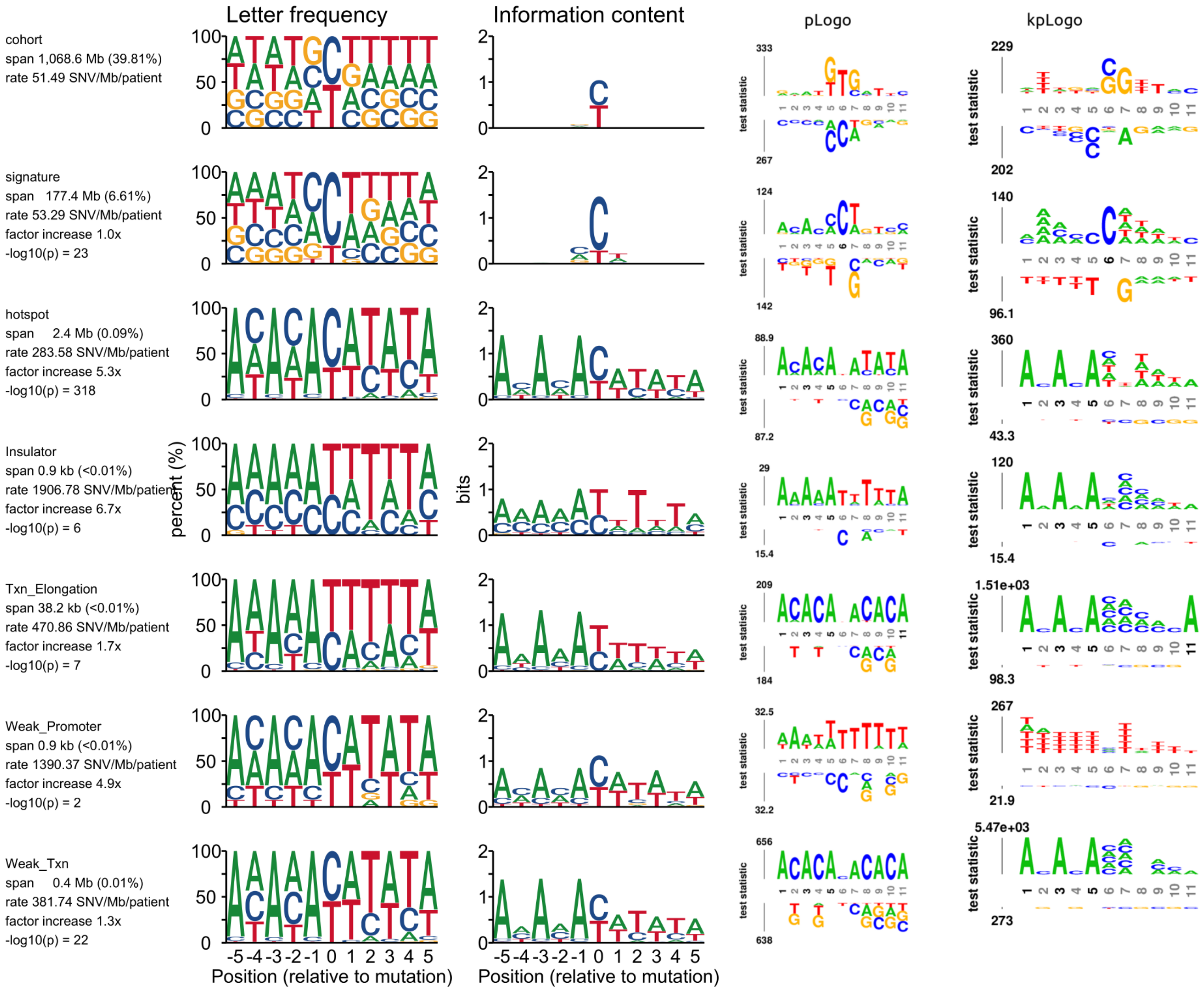

Letter frequency
Information content
pLogo
kpLogo
cohort
span 1,068.6 Mb (39.81%)
rate 51.49 SNV/Mb/patient
signature
span 177.4 Mb (6.61%)
rate 53.29 SNV/Mb/patient
factor increase 1.0x
-log10(p) = 23
hotspot
span 2.4 Mb (0.09%)
rate 283.58 SNV/Mb/patient
factor increase 5.3x
-log10(p) = 318
Insulator
span 0.9 kb (<0.01%)
rate 1906.78 SNV/Mb/patient
factor increase 6.7x
-log10(p) = 6
Txn_Elongation
span 38.2 kb (<0.01%)
rate 470.86 SNV/Mb/patient
factor increase 1.7x
-log10(p) = 7
Weak_Promoter
span 0.9 kb (<0.01%)
rate 1390.37 SNV/Mb/patient
factor increase 4.9x
-log10(p) = 2
Weak_Txn
span 0.4 Mb (0.01%)
rate 381.74 SNV/Mb/patient
factor increase 1.3x
-log10(p) = 22
percent (%)
bits
test statistic
Position (relative to mutation)
Position (relative to mutation)

SBS61

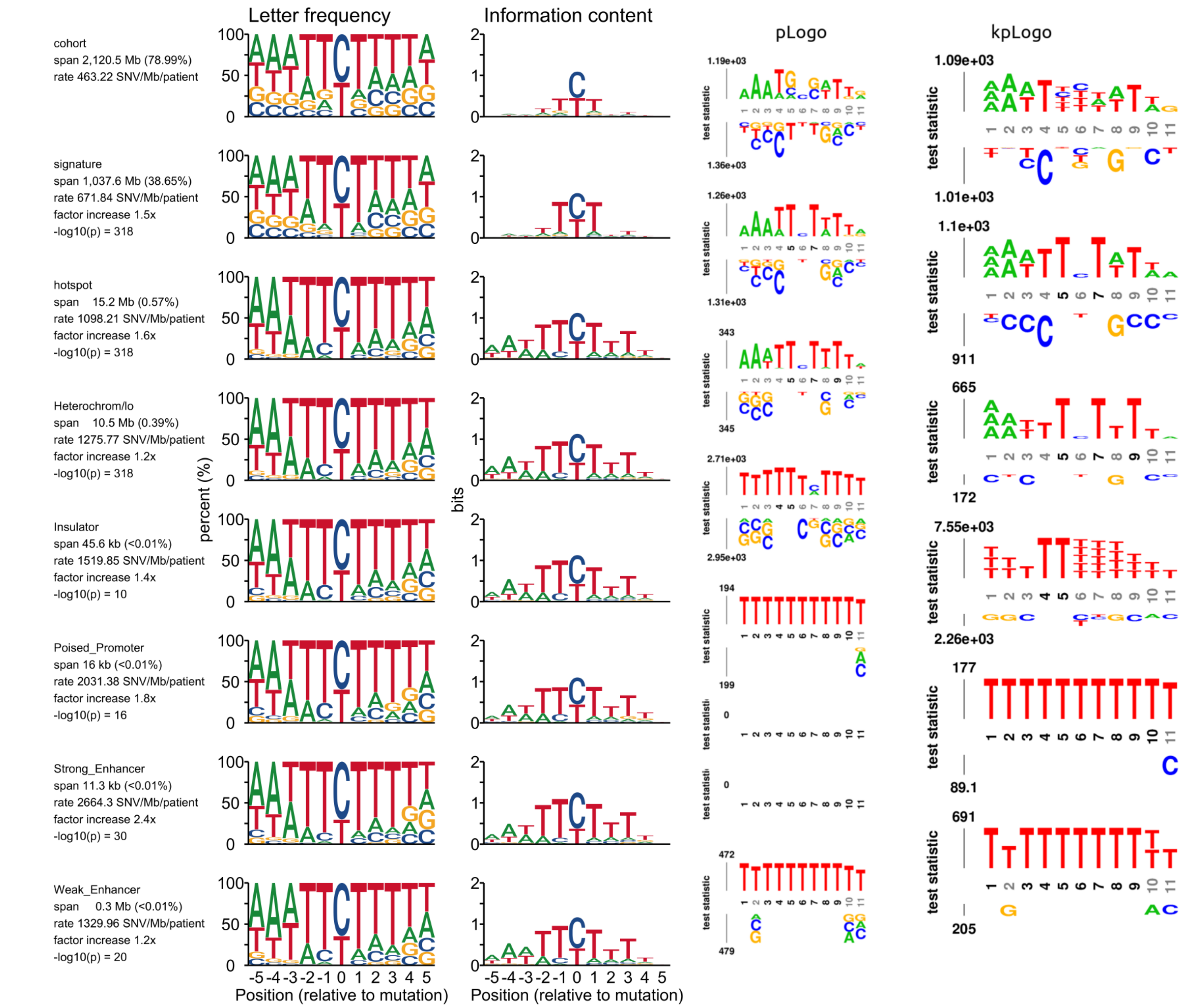
Letter frequency
Information content
pLogo
kpLogo
cohort
span 2,120.5 Mb (78.99%)
rate 463.22 SNV/Mb/patient
signature
span 1,037.6 Mb (38.65%)
rate 671.84 SNV/Mb/patient
factor increase 1.5x
-log10(p) = 318
hotspot
span 15.2 Mb (0.57%)
rate 1098.21 SNV/Mb/patient
factor increase 1.6x
-log10(p) = 318
Heterochrom/lo
span 10.5 Mb (0.39%)
rate 1275.77 SNV/Mb/patient
factor increase 1.2x
-log10(p) = 318
Insulator
span 45.6 kb (<0.01%)
rate 1519.85 SNV/Mb/patient
factor increase 1.4x
-log10(p) = 10
Poised_Promoter
span 16 kb (<0.01%)
rate 2031.38 SNV/Mb/patient
factor increase 1.8x
-log10(p) = 16
Strong_Enhancer
span 11.3 kb (<0.01%)
rate 2664.3 SNV/Mb/patient
factor increase 2.4x
-log10(p) = 30
Weak_Enhancer
span 0.3 Mb (<0.01%)
rate 1329.96 SNV/Mb/patient
factor increase 1.2x
-log10(p) = 20
percent (%)
bits
test statistic
Position (relative to mutation)
Position (relative to mutation)

SBS62

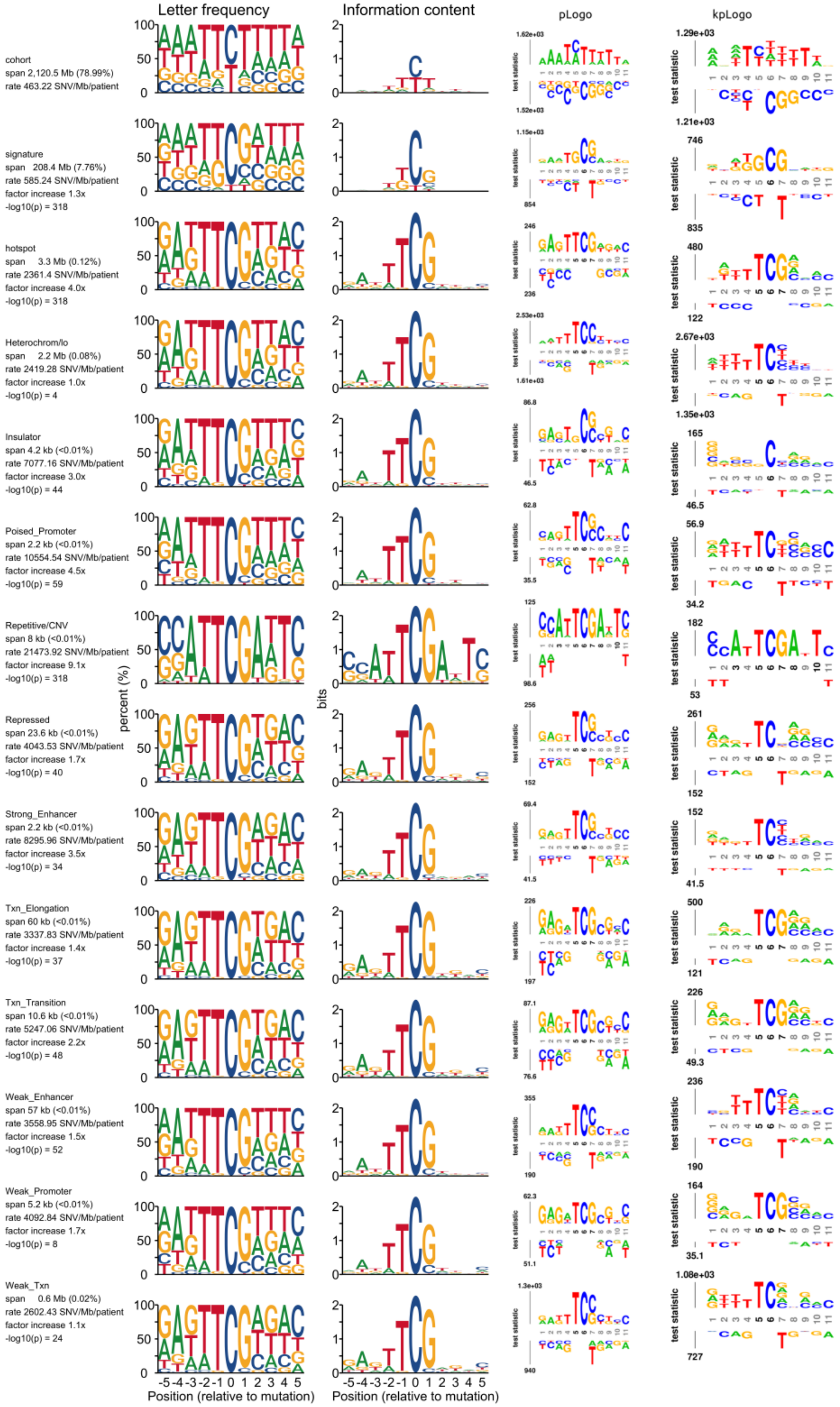

Letter frequency
Information content
pLogo
kpLogo
cohort
span 2,120.5 Mb (78.99%)
rate 463.22 SNV/Mb/patient
signature
span 208.4 Mb (7.76%)
rate 585.24 SNV/Mb/patient
factor increase 1.3x
-log10(p) = 318
hotspot
span 3.3 Mb (0.12%)
rate 2361.4 SNV/Mb/patient
factor increase 4.0x
-log10(p) = 318
Heterochrom/lo
span 2.2 Mb (0.08%)
rate 2419.28 SNV/Mb/patient
factor increase 1.0x
-log10(p) = 4
Insulator
span 4.2 kb (<0.01%)
rate 7077.16 SNV/Mb/patient
factor increase 3.0x
-log10(p) = 44
Poised_Promoter
span 2.2 kb (<0.01%)
rate 10554.54 SNV/Mb/patient
factor increase 4.5x
-log10(p) = 59
Repetitive/CNV
span 8 kb (<0.01%)
rate 21473.92 SNV/Mb/patient
factor increase 9.1x
-log10(p) = 318
Repressed
span 23.6 kb (<0.01%)
rate 4043.53 SNV/Mb/patient
factor increase 1.7x
-log10(p) = 40
Strong_Enhancer
span 2.2 kb (<0.01%)
rate 8295.96 SNV/Mb/patient
factor increase 3.5x
-log10(p) = 34
Txn_Elongation
span 60 kb (<0.01%)
rate 3337.83 SNV/Mb/patient
factor increase 1.4x
-log10(p) = 37
Txn_Transition
span 10.6 kb (<0.01%)
rate 5247.06 SNV/Mb/patient
factor increase 2.2x
-log10(p) = 48
Weak_Enhancer
span 57 kb (<0.01%)
rate 3558.95 SNV/Mb/patient
factor increase 1.5x
-log10(p) = 52
Weak_Promoter
span 5.2 kb (<0.01%)
rate 4092.84 SNV/Mb/patient
factor increase 1.7x
-log10(p) = 8
Weak_Txn
span 0.6 Mb (0.02%)
rate 2602.43 SNV/Mb/patient
factor increase 1.1x
-log10(p) = 24
percent (%)
bits
test statistic
Position (relative to mutation)
Position (relative to mutation)

SBS63

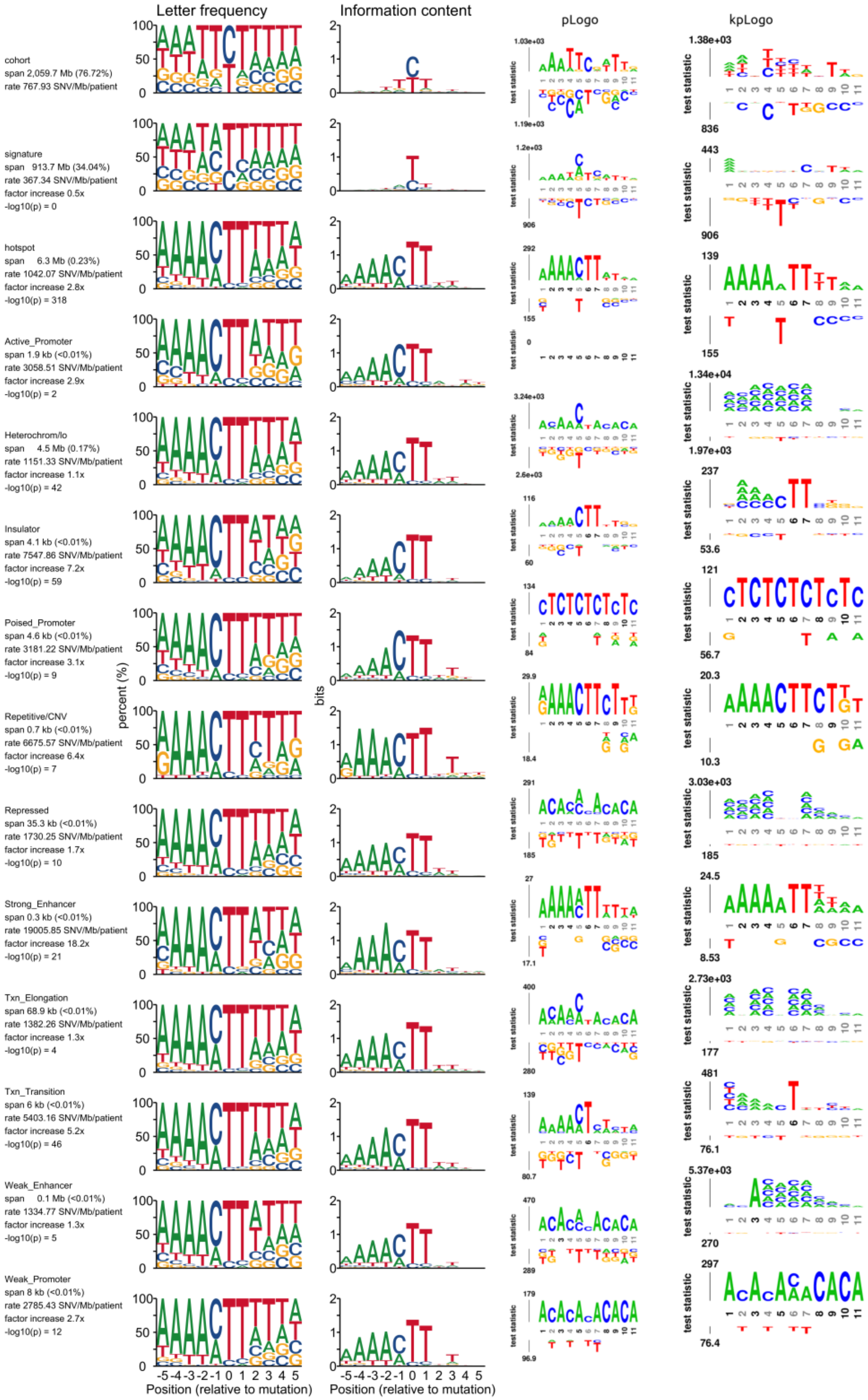
Letter frequency
Information content
pLogo
kpLogo
cohort
span 2,059.7 Mb (76.72%)
rate 767.93 SNV/Mb/patient
signature
span 913.7 Mb (34.04%)
rate 367.34 SNV/Mb/patient
factor increase 0.5x
-log10(p) = 0
hotspot
span 6.3 Mb (0.23%)
rate 1042.07 SNV/Mb/patient
factor increase 2.8x
-log10(p) = 318
Active_Promoter
span 1.9 kb (<0.01%)
rate 3058.51 SNV/Mb/patient
factor increase 2.9x
-log10(p) = 2
Heterochrom/lo
span 4.5 Mb (0.17%)
rate 1151.33 SNV/Mb/patient
factor increase 1.1x
-log10(p) = 42
Insulator
span 4.1 kb (<0.01%)
rate 7547.86 SNV/Mb/patient
factor increase 7.2x
-log10(p) = 59
Poised_Promoter
span 4.6 kb (<0.01%)
rate 3181.22 SNV/Mb/patient
factor increase 3.1x
-log10(p) = 9
Repetitive/CNV
span 0.7 kb (<0.01%)
rate 6675.57 SNV/Mb/patient
factor increase 6.4x
-log10(p) = 7
Repressed
span 35.3 kb (<0.01%)
rate 1730.25 SNV/Mb/patient
factor increase 1.7x
-log10(p) = 10
Strong_Enhancer
span 0.3 kb (<0.01%)
rate 19005.85 SNV/Mb/patient
factor increase 18.2x
-log10(p) = 21
Txn_Elongation
span 68.9 kb (<0.01%)
rate 1382.26 SNV/Mb/patient
factor increase 1.3x
-log10(p) = 4
Txn_Transition
span 6 kb (<0.01%)
rate 5403.16 SNV/Mb/patient
factor increase 5.2x
-log10(p) = 46
Weak_Enhancer
span 0.1 Mb (<0.01%)
rate 1334.77 SNV/Mb/patient
factor increase 1.3x
-log10(p) = 5
Weak_Promoter
span 8 kb (<0.01%)
rate 2785.43 SNV/Mb/patient
factor increase 2.7x
-log10(p) = 12
percent (%)
bits
test statistic
Position (relative to mutation)
Position (relative to mutation)

SBS64

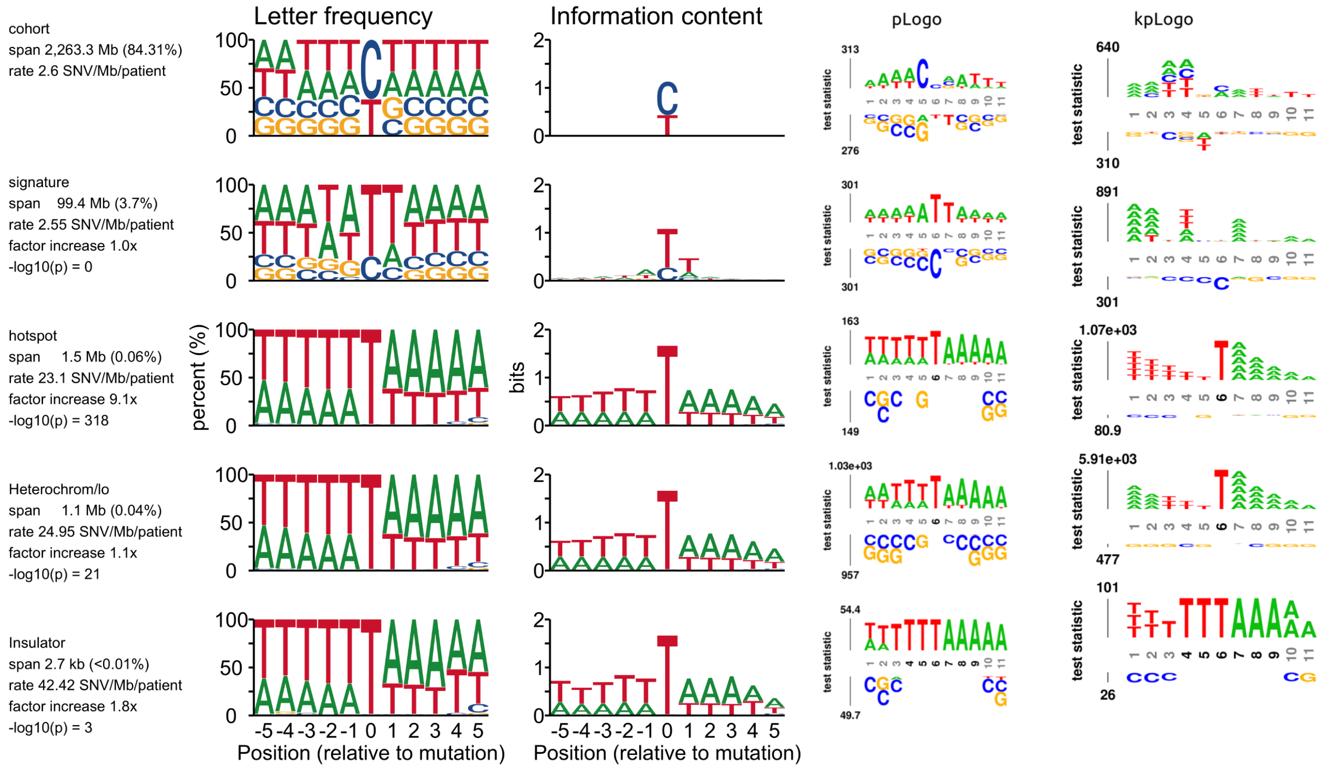
Letter frequency
Information content
pLogo
kpLogo
cohort
span 2,263.3 Mb (84.31%)
rate 2.6 SNV/Mb/patient
signature
span 99.4 Mb (3.7%)
rate 2.55 SNV/Mb/patient
factor increase 1.0x
-log10(p) = 0
hotspot
span 1.5 Mb (0.06%)
rate 23.1 SNV/Mb/patient
factor increase 9.1x
-log10(p) = 318
Heterochrom/lo
span 1.1 Mb (0.04%)
rate 24.95 SNV/Mb/patient
factor increase 1.1x
-log10(p) = 21
Insulator
span 2.7 kb (<0.01%)
rate 42.42 SNV/Mb/patient
factor increase 1.8x
-log10(p) = 3
percent (%)
bits
test statistic
Position (relative to mutation)
Position (relative to mutation)

SBS65

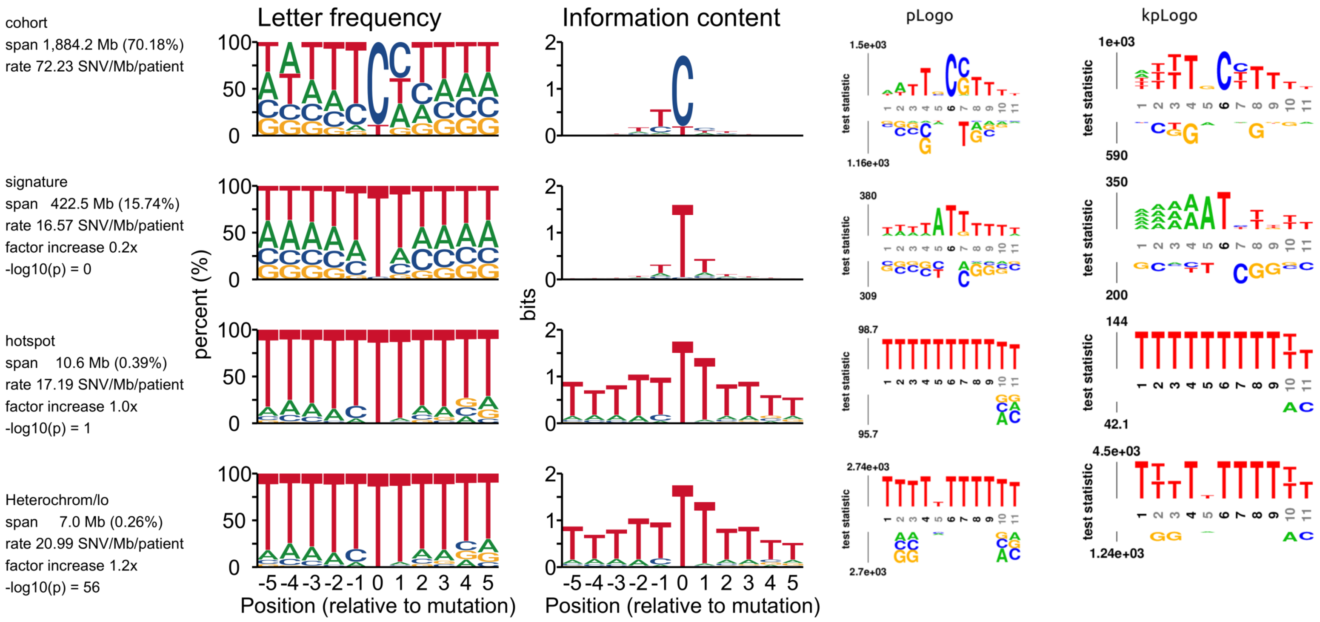
Letter frequency
Information content
pLogo
kpLogo
cohort
span 1,884.2 Mb (70.18%)
rate 72.23 SNV/Mb/patient
signature
span 422.5 Mb (15.74%)
rate 16.57 SNV/Mb/patient
factor increase 0.2x
-log10(p) = 0
hotspot
span 10.6 Mb (0.39%)
rate 17.19 SNV/Mb/patient
factor increase 1.0x
-log10(p) = 1
Heterochrom/lo
span 7.0 Mb (0.26%)
rate 20.99 SNV/Mb/patient
factor increase 1.2x
-log10(p) = 56
percent (%)
bits
test statistic
Position (relative to mutation)
Position (relative to mutation)

SBS66

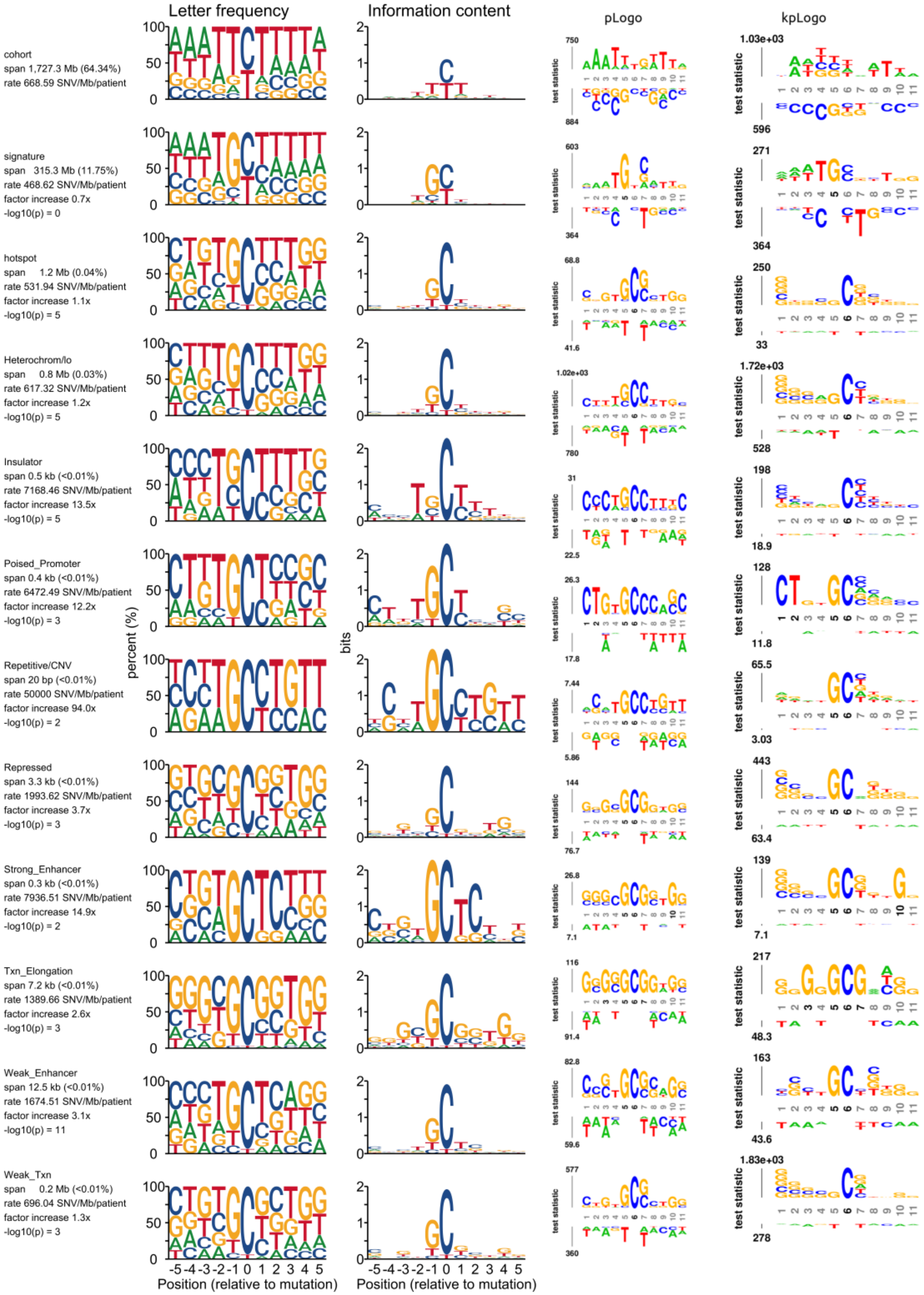

Letter frequency
Information content
pLogo
kpLogo
cohort
span 1,727.3 Mb (64.34%)
rate 668.59 SNV/Mb/patient
signature
span 315.3 Mb (11.75%)
rate 468.62 SNV/Mb/patient
factor increase 0.7x
-log10(p) = 0
hotspot
span 1.2 Mb (0.04%)
rate 531.94 SNV/Mb/patient
factor increase 1.1x
-log10(p) = 5
Heterochrom/lo
span 0.8 Mb (0.03%)
rate 617.32 SNV/Mb/patient
factor increase 1.2x
-log10(p) = 5
Insulator
span 0.5 kb (<0.01%)
rate 7168.46 SNV/Mb/patient
factor increase 13.5x
-log10(p) = 5
Poised_Promoter
span 0.4 kb (<0.01%)
rate 6472.49 SNV/Mb/patient
factor increase 12.2x
-log10(p) = 3
Repetitive/CNV
span 20 bp (<0.01%)
rate 50000 SNV/Mb/patient
factor increase 94.0x
-log10(p) = 2
Repressed
span 3.3 kb (<0.01%)
rate 1993.62 SNV/Mb/patient
factor increase 3.7x
-log10(p) = 3
Strong_Enhancer
span 0.3 kb (<0.01%)
rate 7936.51 SNV/Mb/patient
factor increase 14.9x
-log10(p) = 2
Txn_Elongation
span 7.2 kb (<0.01%)
rate 1389.66 SNV/Mb/patient
factor increase 2.6x
-log10(p) = 3
Weak_Enhancer
span 12.5 kb (<0.01%)
rate 1674.51 SNV/Mb/patient
factor increase 3.1x
-log10(p) = 11
Weak_Txn
span 0.2 Mb (<0.01%)
rate 696.04 SNV/Mb/patient
factor increase 1.3x
-log10(p) = 3
percent (%)
bits
test statistic
Position (relative to mutation)
Position (relative to mutation)

SBS67

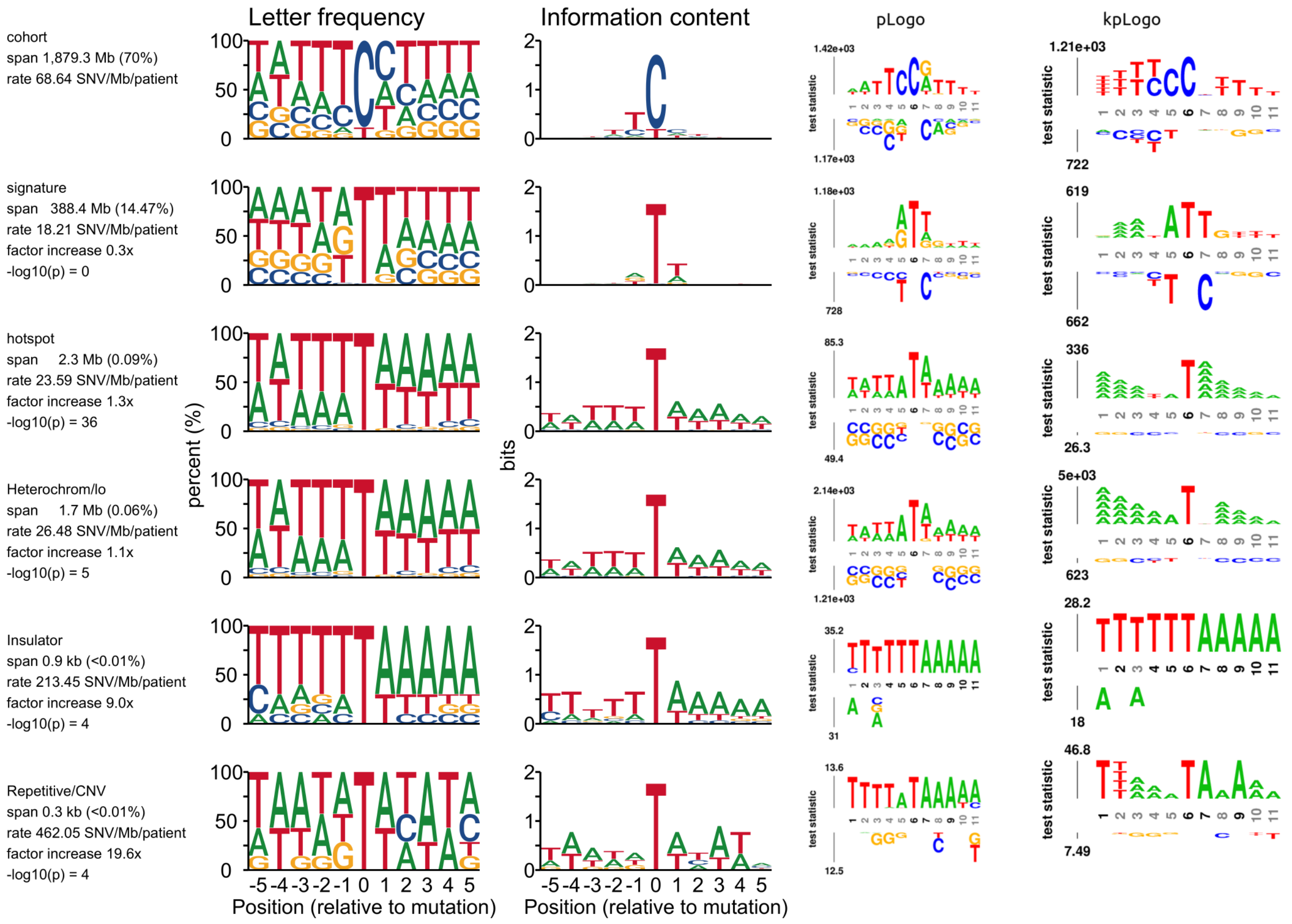
Letter frequency
Information content
pLogo
kpLogo
cohort
span 1,879.3 Mb (70%)
rate 68.64 SNV/Mb/patient
signature
span 388.4 Mb (14.47%)
rate 18.21 SNV/Mb/patient
factor increase 0.3x
-log10(p) = 0
hotspot
span 2.3 Mb (0.09%)
rate 23.59 SNV/Mb/patient
factor increase 1.3x
-log10(p) = 36
Heterochrom/lo
span 1.7 Mb (0.06%)
rate 26.48 SNV/Mb/patient
factor increase 1.1x
-log10(p) = 5
Insulator
span 0.9 kb (<0.01%)
rate 213.45 SNV/Mb/patient
factor increase 9.0x
-log10(p) = 4
Repetitive/CNV
span 0.3 kb (<0.01%)
rate 462.05 SNV/Mb/patient
factor increase 19.6x
-log10(p) = 4
percent (%)
bits
test statistic
Position (relative to mutation)
Position (relative to mutation)

SBS69

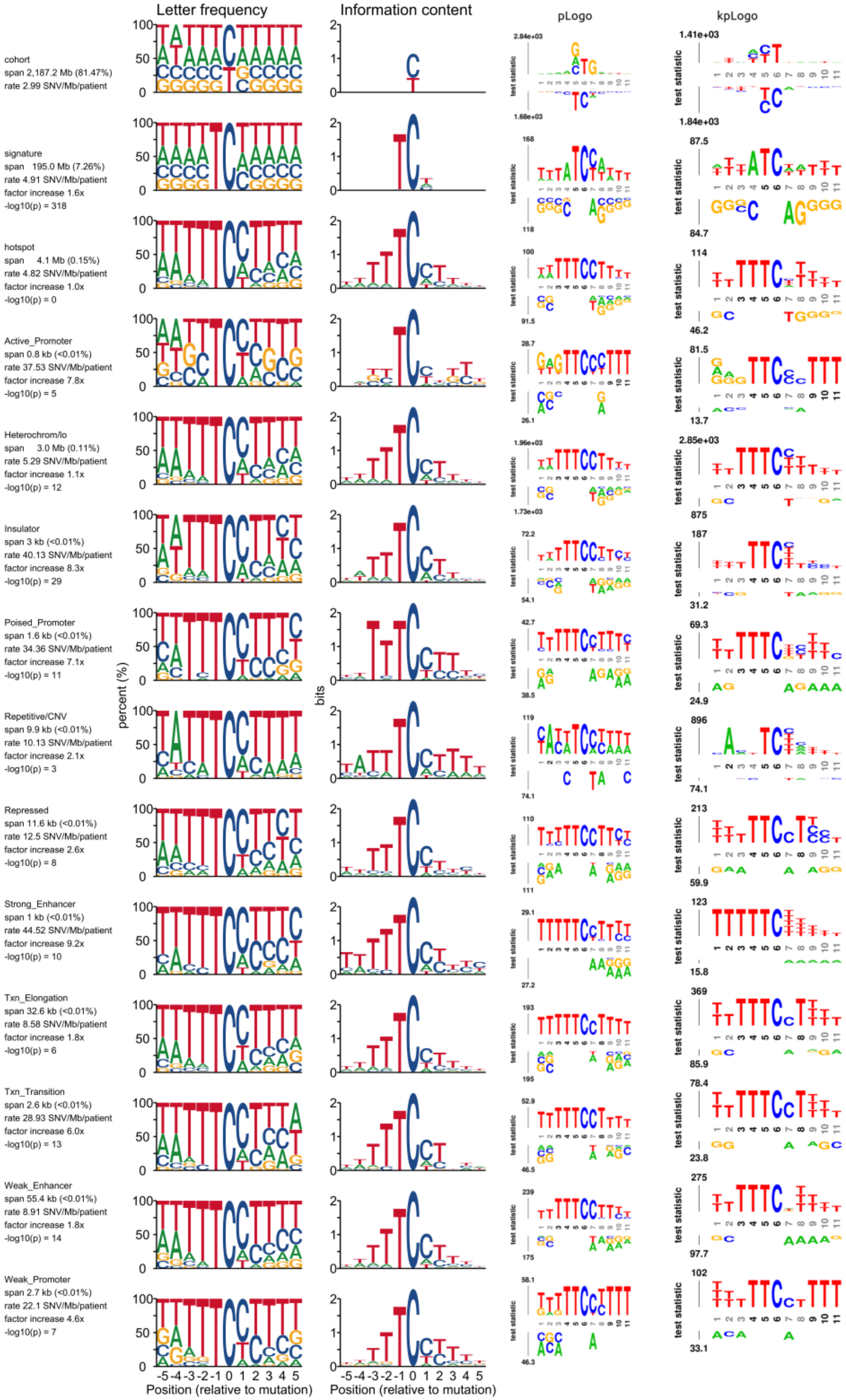
Letter frequency
Information content
pLogo
kpLogo
cohort
span 2,187.2 Mb (81.47%)
rate 2.99 SNV/Mb/patient
signature
span 195.0 Mb (7.26%)
rate 4.91 SNV/Mb/patient
factor increase 1.6x
-log10(p) = 318
hotspot
span 4.1 Mb (0.15%)
rate 4.82 SNV/Mb/patient
factor increase 1.0x
-log10(p) = 0
Active_Promoter
span 0.8 kb (<0.01%)
rate 37.53 SNV/Mb/patient
factor increase 7.8x
-log10(p) = 5
Heterochrom/lo
span 3.0 Mb (0.11%)
rate 5.29 SNV/Mb/patient
factor increase 1.1x
-log10(p) = 12
Insulator
span 3 kb (<0.01%)
rate 40.13 SNV/Mb/patient
factor increase 8.3x
-log10(p) = 29
Poised_Promoter
span 1.6 kb (<0.01%)
rate 34.36 SNV/Mb/patient
factor increase 7.1x
-log10(p) = 11
Repetitive/CNV
span 9.9 kb (<0.01%)
rate 10.13 SNV/Mb/patient
factor increase 2.1x
-log10(p) = 3
Repressed
span 11.6 kb (<0.01%)
rate 12.5 SNV/Mb/patient
factor increase 2.6x
-log10(p) = 8
Strong_Enhancer
span 1 kb (<0.01%)
rate 44.52 SNV/Mb/patient
factor increase 9.2x
-log10(p) = 10
Txn_Elongation
span 32.6 kb (<0.01%)
rate 8.58 SNV/Mb/patient
factor increase 1.8x
-log10(p) = 6
Txn_Transition
span 2.6 kb (<0.01%)
rate 28.93 SNV/Mb/patient
factor increase 6.0x
-log10(p) = 13
Weak_Enhancer
span 55.4 kb (<0.01%)
rate 8.91 SNV/Mb/patient
factor increase 1.8x
-log10(p) = 14
Weak_Promoter
span 2.7 kb (<0.01%)
rate 22.1 SNV/Mb/patient
factor increase 4.6x
-log10(p) = 7
percent (%)
bits
test statistic
Position (relative to mutation)
Position (relative to mutation)

SBS70

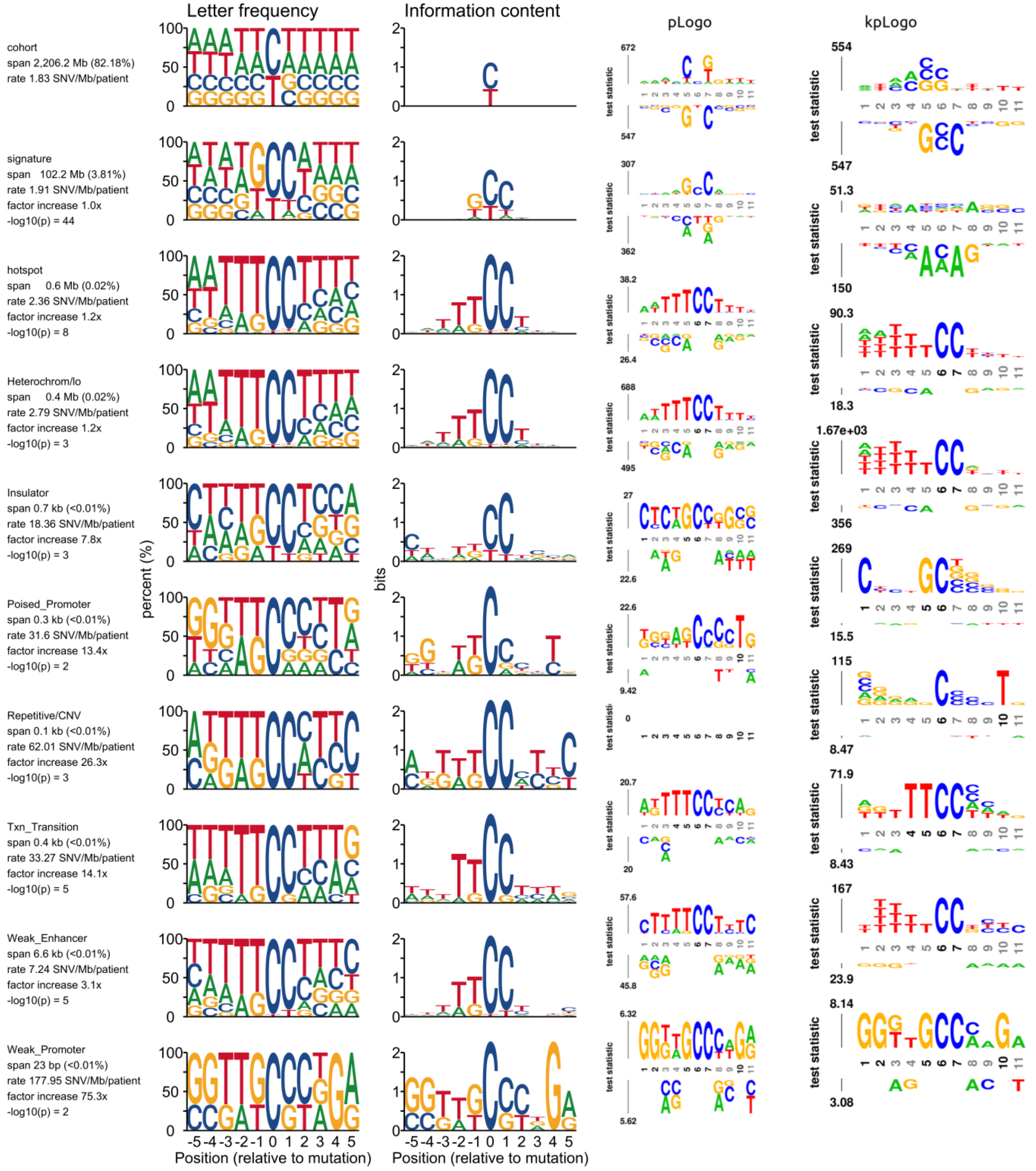
Letter frequency
Information content
pLogo
kpLogo
cohort
span 2,206.2 Mb (82.18%)
rate 1.83 SNV/Mb/patient
signature
span 102.2 Mb (3.81%)
rate 1.91 SNV/Mb/patient
factor increase 1.0x
-log10(p) = 44
hotspot
span 0.6 Mb (0.02%)
rate 2.36 SNV/Mb/patient
factor increase 1.2x
-log10(p) = 8
Heterochrom/lo
span 0.4 Mb (0.02%)
rate 2.79 SNV/Mb/patient
factor increase 1.2x
-log10(p) = 3
Insulator
span 0.7 kb (<0.01%)
rate 18.36 SNV/Mb/patient
factor increase 7.8x
-log10(p) = 3
Poised_Promoter
span 0.3 kb (<0.01%)
rate 31.6 SNV/Mb/patient
factor increase 13.4x
-log10(p) = 2
Repetitive/CNV
span 0.1 kb (<0.01%)
rate 62.01 SNV/Mb/patient
factor increase 26.3x
-log10(p) = 3
Txn_Transition
span 0.4 kb (<0.01%)
rate 33.27 SNV/Mb/patient
factor increase 14.1x
-log10(p) = 5
Weak_Enhancer
span 6.6 kb (<0.01%)
rate 7.24 SNV/Mb/patient
factor increase 3.1x
-log10(p) = 5
Weak_Promoter
span 23 bp (<0.01%)
rate 177.95 SNV/Mb/patient
factor increase 75.3x
-log10(p) = 2
percent (%)
bits
test statistic
Position (relative to mutation)
Position (relative to mutation)

SBS72

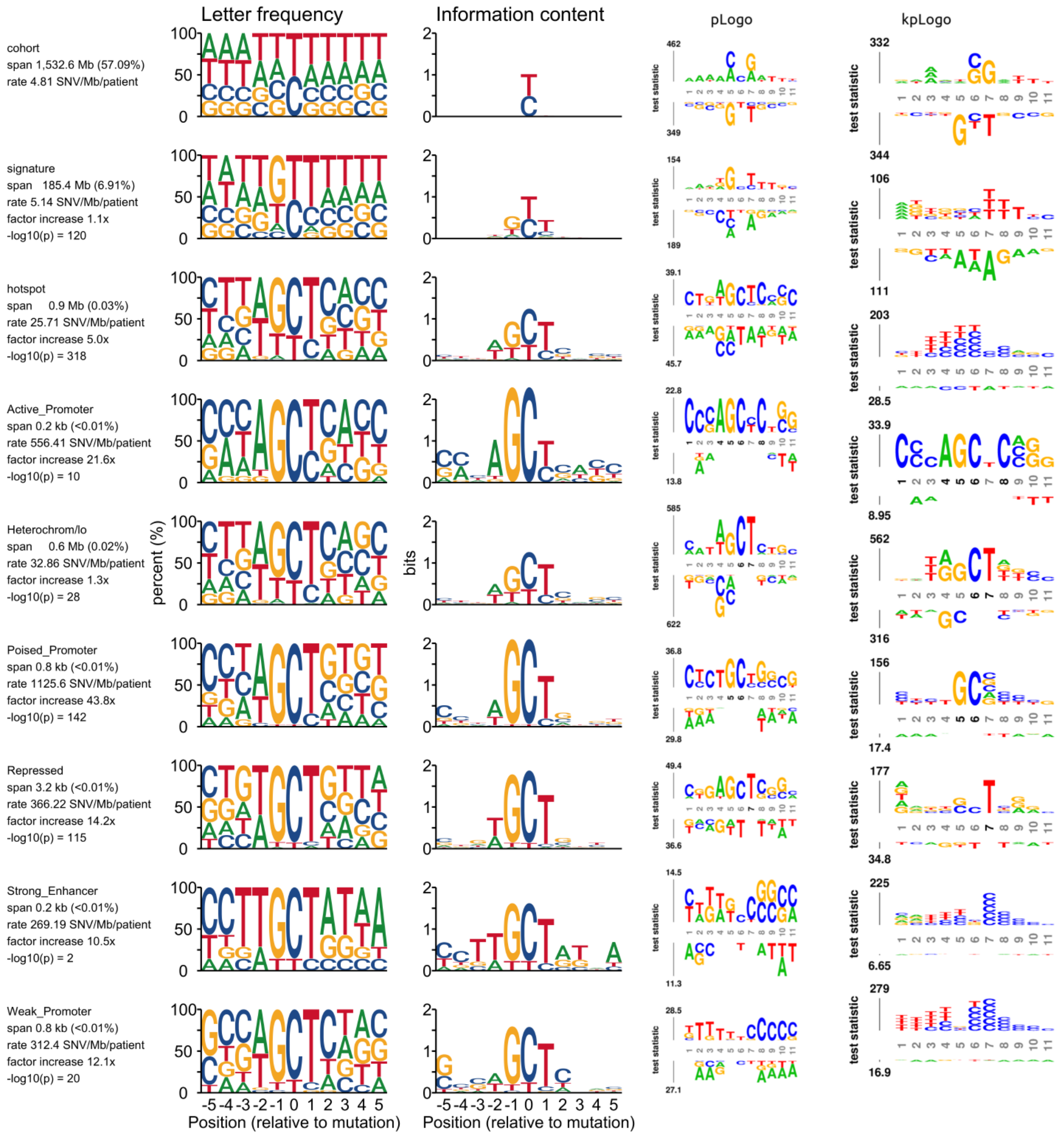

Letter frequency
Information content
pLogo
kpLogo
cohort
span 1,532.6 Mb (57.09%)
rate 4.81 SNV/Mb/patient
signature
span 185.4 Mb (6.91%)
rate 5.14 SNV/Mb/patient
factor increase 1.1x
-log10(p) = 120
hotspot
span 0.9 Mb (0.03%)
rate 25.71 SNV/Mb/patient
factor increase 5.0x
-log10(p) = 318
Active_Promoter
span 0.2 kb (<0.01%)
rate 556.41 SNV/Mb/patient
factor increase 21.6x
-log10(p) = 10
Heterochrom/lo
span 0.6 Mb (0.02%)
rate 32.86 SNV/Mb/patient
factor increase 1.3x
-log10(p) = 28
Poised_Promoter
span 0.8 kb (<0.01%)
rate 1125.6 SNV/Mb/patient
factor increase 43.8x
-log10(p) = 142
Repressed
span 3.2 kb (<0.01%)
rate 366.22 SNV/Mb/patient
factor increase 14.2x
-log10(p) = 115
Strong_Enhancer
span 0.2 kb (<0.01%)
rate 269.19 SNV/Mb/patient
factor increase 10.5x
-log10(p) = 2
Weak_Promoter
span 0.8 kb (<0.01%)
rate 312.4 SNV/Mb/patient
factor increase 12.1x
-log10(p) = 20
percent (%)
bits
test statistic
Position (relative to mutation)
Position (relative to mutation)

SBS73

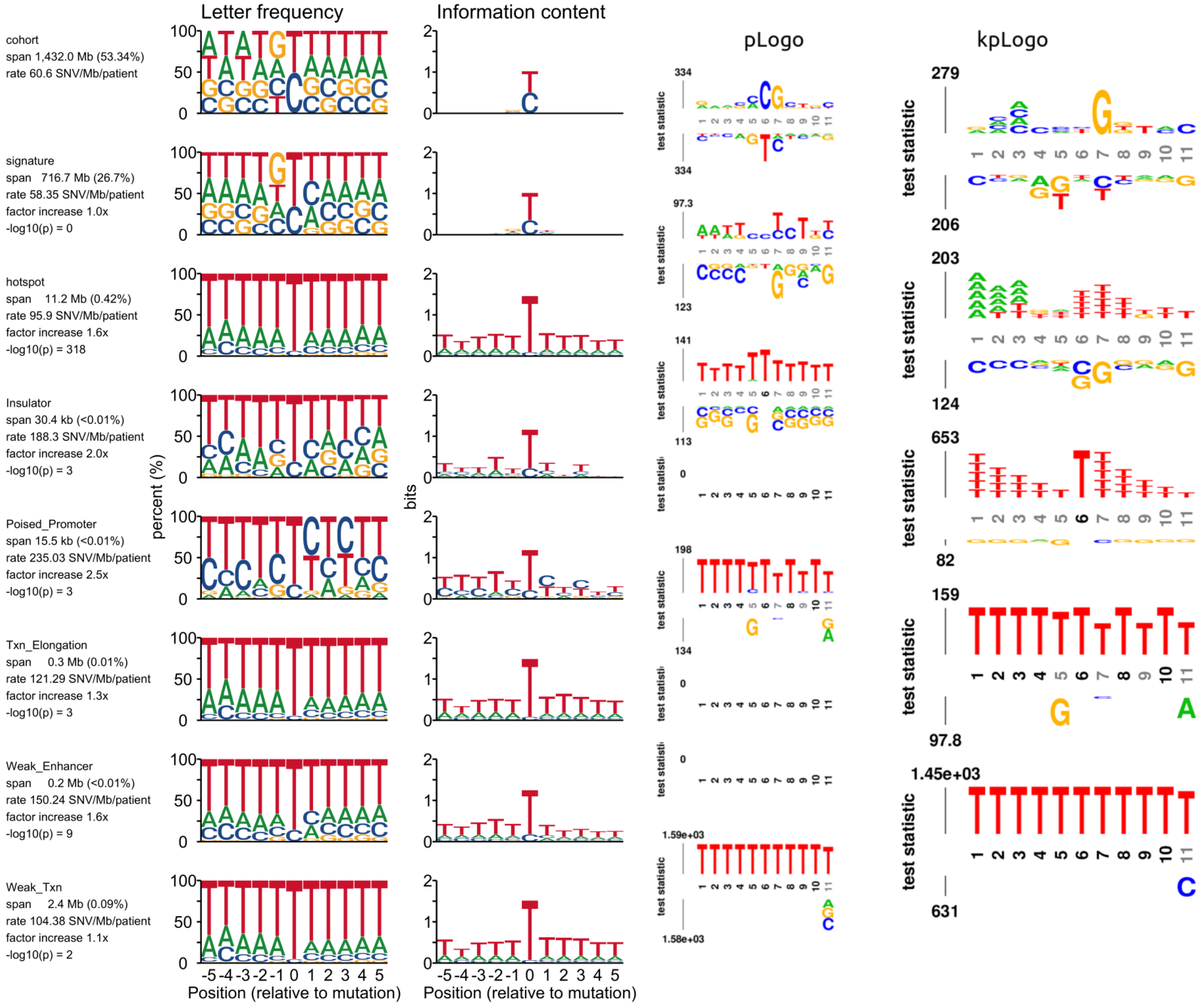
Letter frequency
Information content
pLogo
kpLogo
cohort
span 1,432.0 Mb (53.34%)
rate 60.6 SNV/Mb/patient
signature
span 716.7 Mb (26.7%)
rate 58.35 SNV/Mb/patient
factor increase 1.0x
-log10(p) = 0
hotspot
span 11.2 Mb (0.42%)
rate 95.9 SNV/Mb/patient
factor increase 1.6x
-log10(p) = 318
Insulator
span 30.4 kb (<0.01%)
rate 188.3 SNV/Mb/patient
factor increase 2.0x
-log10(p) = 3
Poised_Promoter
span 15.5 kb (<0.01%)
rate 235.03 SNV/Mb/patient
factor increase 2.5x
-log10(p) = 3
Txn_Elongation
span 0.3 Mb (0.01%)
rate 121.29 SNV/Mb/patient
factor increase 1.3x
-log10(p) = 3
Weak_Enhancer
span 0.2 Mb (<0.01%)
rate 150.24 SNV/Mb/patient
factor increase 1.6x
-log10(p) = 9
Weak_Txn
span 2.4 Mb (0.09%)
rate 104.38 SNV/Mb/patient
factor increase 1.1x
-log10(p) = 2
percent (%)
bits
test statistic
Position (relative to mutation)
Position (relative to mutation)

SBS74

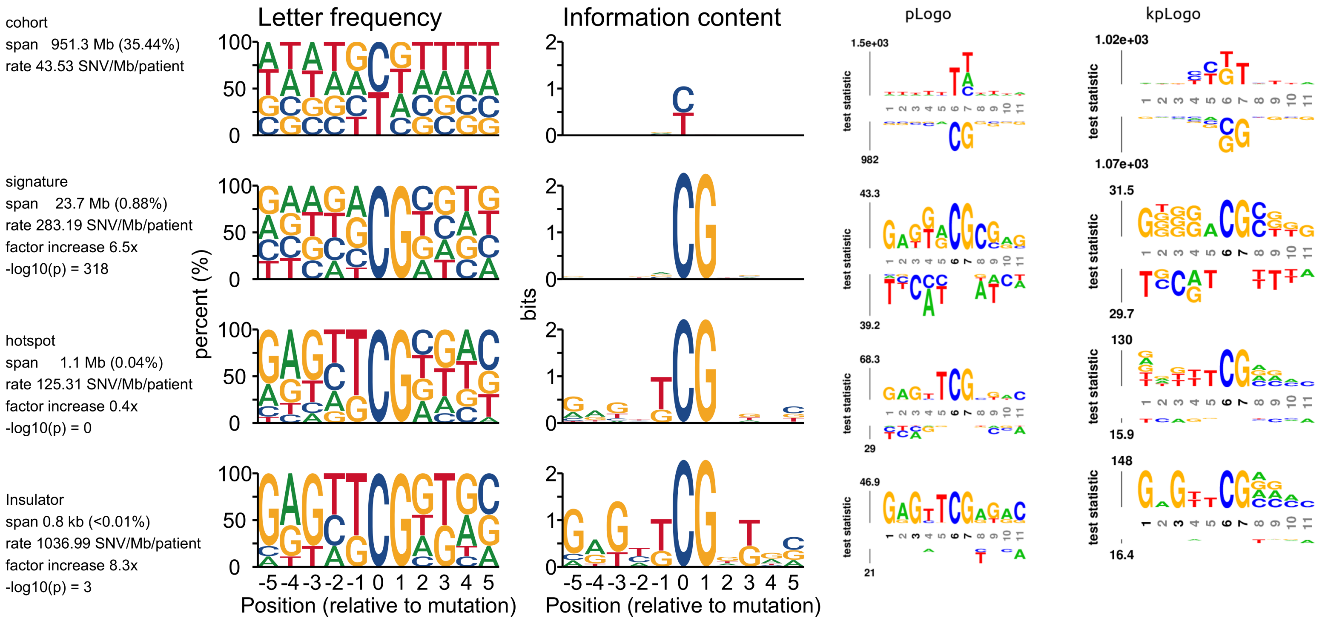
cohort
span 951.3 Mb (35.44%)
rate 43.53 SNV/Mb/patient
signature
span 23.7 Mb (0.88%)
rate 283.19 SNV/Mb/patient
factor increase 6.5x
-log10(p) = 318
hotspot
span 1.1 Mb (0.04%)
rate 125.31 SNV/Mb/patient
factor increase 0.4x
-log10(p) = 0
Insulator
span 0.8 kb (<0.01%)
rate 1036.99 SNV/Mb/patient
factor increase 8.3x
-log10(p) = 3
Letter frequency
percent (%)
Position (relative to mutation)
Information content
bits
Position (relative to mutation)
pLogo
test statistic
1.5e+03
982
43.3
39.2
68.3
29
46.9
21
kpLogo
1.02e+03
1.07e+03
31.5
29.7
130
15.9
148
16.4

SBS75

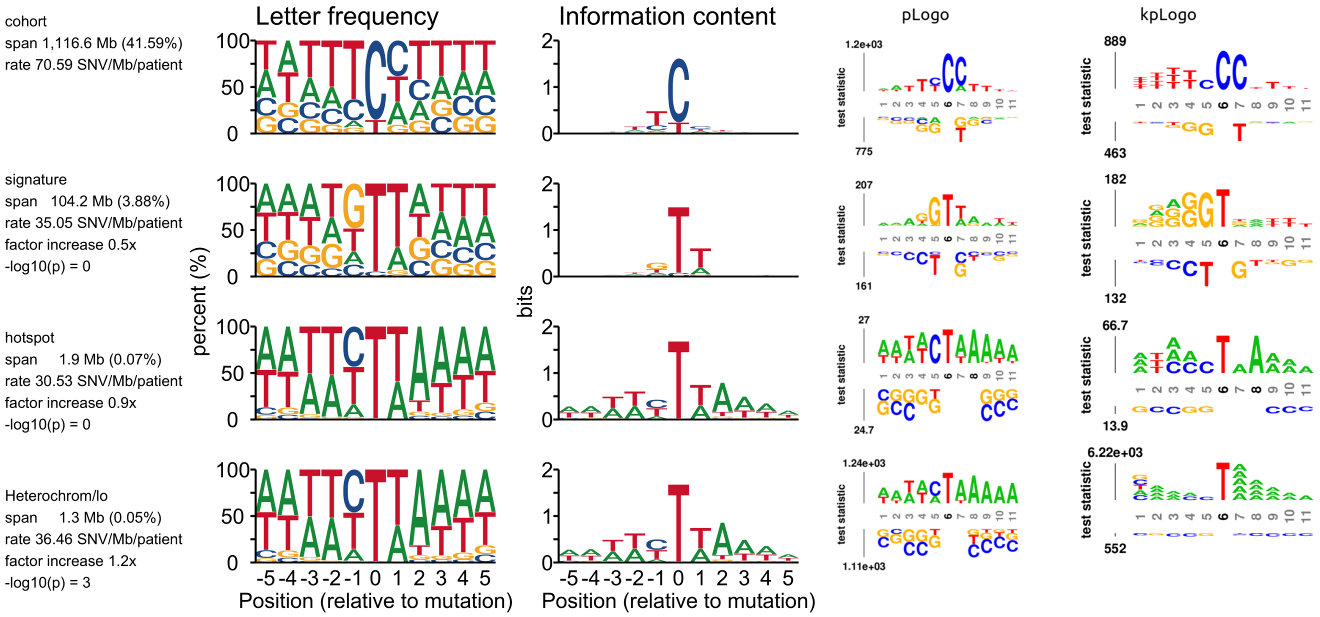
Letter frequency
Information content
pLogo
kpLogo
cohort
span 1,116.6 Mb (41.59%)
rate 70.59 SNV/Mb/patient
signature
span 104.2 Mb (3.88%)
rate 35.05 SNV/Mb/patient
factor increase 0.5x
-log10(p) = 0
hotspot
span 1.9 Mb (0.07%)
rate 30.53 SNV/Mb/patient
factor increase 0.9x
-log10(p) = 0
Heterochrom/lo
span 1.3 Mb (0.05%)
rate 36.46 SNV/Mb/patient
factor increase 1.2x
-log10(p) = 3
percent (%)
bits
Position (relative to mutation)
Position (relative to mutation)
test statistic

SBS76

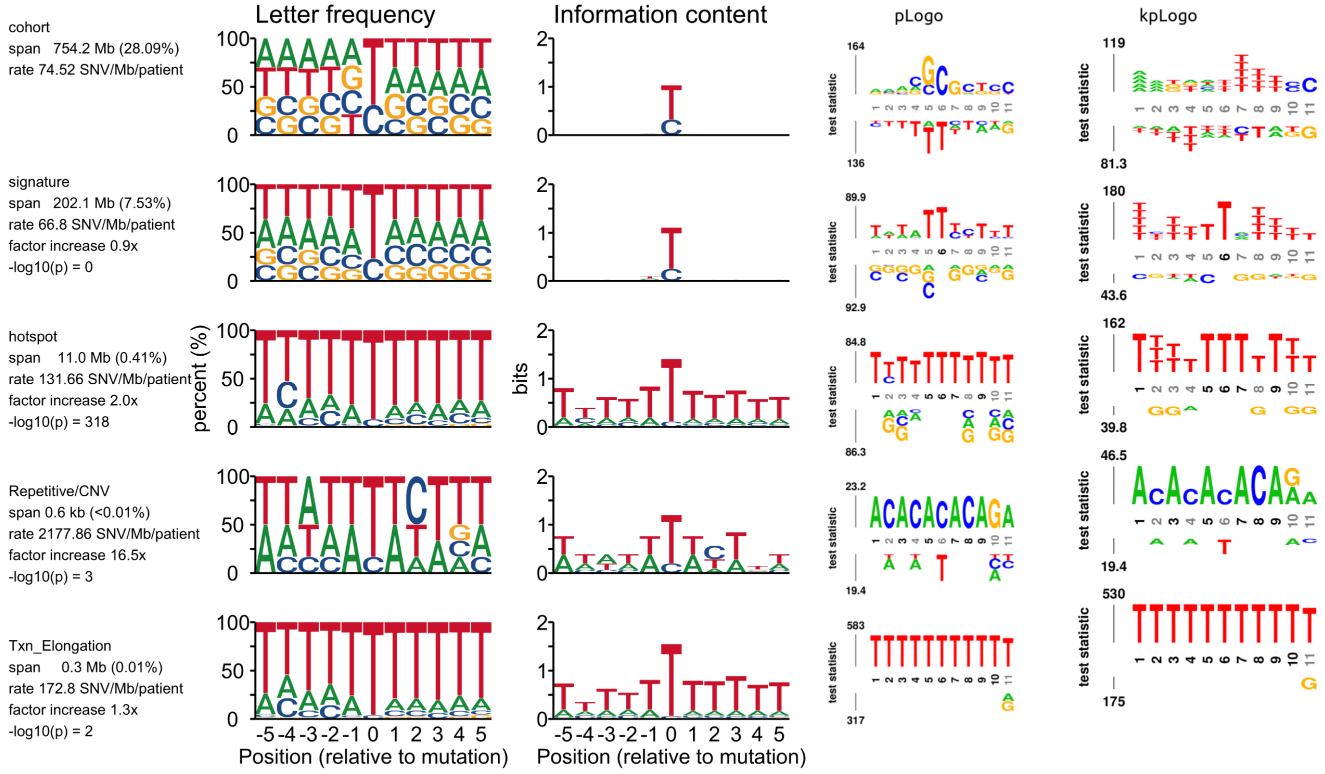
cohort
span 754.2 Mb (28.09%)
rate 74.52 SNV/Mb/patient
signature
span 202.1 Mb (7.53%)
rate 66.8 SNV/Mb/patient
factor increase 0.9x
-log10(p) = 0
hotspot
span 11.0 Mb (0.41%)
rate 131.66 SNV/Mb/patient
factor increase 2.0x
-log10(p) = 318
Repetitive/CNV
span 0.6 kb (<0.01%)
rate 2177.86 SNV/Mb/patient
factor increase 16.5x
-log10(p) = 3
Txn_Elongation
span 0.3 Mb (0.01%)
rate 172.8 SNV/Mb/patient
factor increase 1.3x
-log10(p) = 2
Letter frequency
Information content
pLogo
kpLogo
percent (%)
bits
test statistic
Position (relative to mutation)
Position (relative to mutation)

SBS77

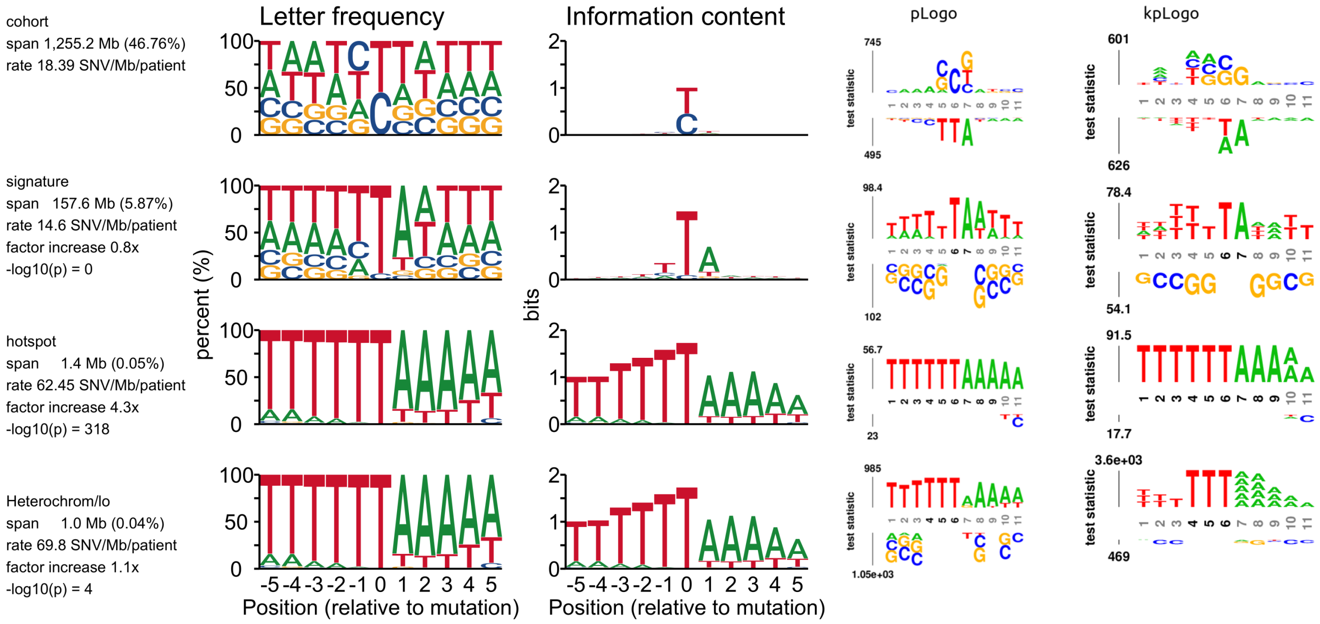
cohort
span 1,255.2 Mb (46.76%)
rate 18.39 SNV/Mb/patient
signature
span 157.6 Mb (5.87%)
rate 14.6 SNV/Mb/patient
factor increase 0.8x
-log10(p) = 0
hotspot
span 1.4 Mb (0.05%)
rate 62.45 SNV/Mb/patient
factor increase 4.3x
-log10(p) = 318
Heterochrom/lo
span 1.0 Mb (0.04%)
rate 69.8 SNV/Mb/patient
factor increase 1.1x
-log10(p) = 4
Letter frequency
percent (%)
Position (relative to mutation)
Information content
bits
Position (relative to mutation)
pLogo
kpLogo
test statistic

SBS78

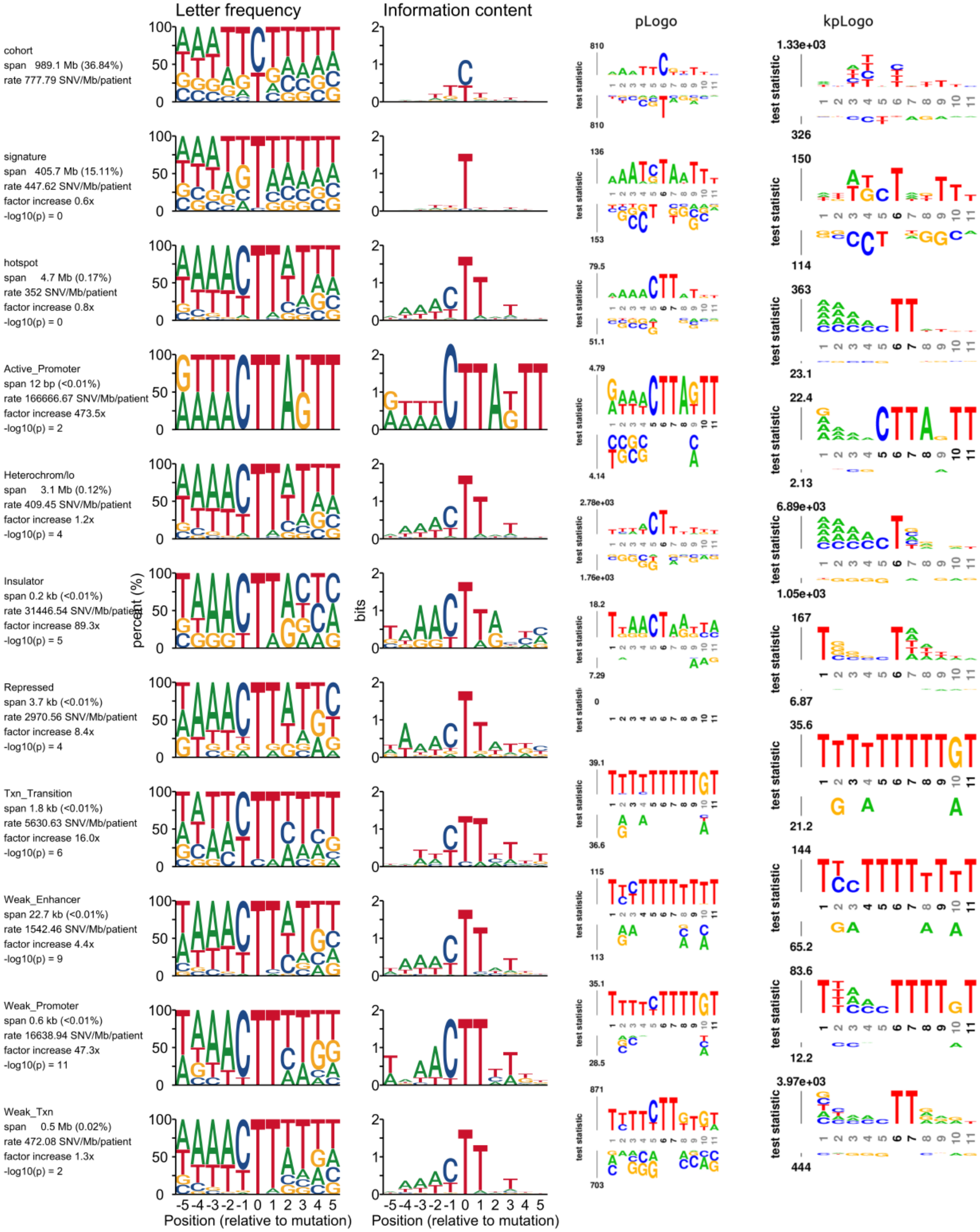
Letter frequency
Information content
pLogo
kpLogo
cohort
span 989.1 Mb (36.84%)
rate 777.79 SNV/Mb/patient
signature
span 405.7 Mb (15.11%)
rate 447.62 SNV/Mb/patient
factor increase 0.6x
-log10(p) = 0
hotspot
span 4.7 Mb (0.17%)
rate 352 SNV/Mb/patient
factor increase 0.8x
-log10(p) = 0
Active_Promoter
span 12 bp (<0.01%)
rate 166666.67 SNV/Mb/patient
factor increase 473.5x
-log10(p) = 2
Heterochrom/lo
span 3.1 Mb (0.12%)
rate 409.45 SNV/Mb/patient
factor increase 1.2x
-log10(p) = 4
Insulator
span 0.2 kb (<0.01%)
rate 31446.54 SNV/Mb/patient
factor increase 89.3x
-log10(p) = 5
Repressed
span 3.7 kb (<0.01%)
rate 2970.56 SNV/Mb/patient
factor increase 8.4x
-log10(p) = 4
Txn_Transition
span 1.8 kb (<0.01%)
rate 5630.63 SNV/Mb/patient
factor increase 16.0x
-log10(p) = 6
Weak_Enhancer
span 22.7 kb (<0.01%)
rate 1542.46 SNV/Mb/patient
factor increase 4.4x
-log10(p) = 9
Weak_Promoter
span 0.6 kb (<0.01%)
rate 16638.94 SNV/Mb/patient
factor increase 47.3x
-log10(p) = 11
Weak_Txn
span 0.5 Mb (0.02%)
rate 472.08 SNV/Mb/patient
factor increase 1.3x
-log10(p) = 2
percent (%)
bits
test statistic
Position (relative to mutation)
Position (relative to mutation)

SBS79

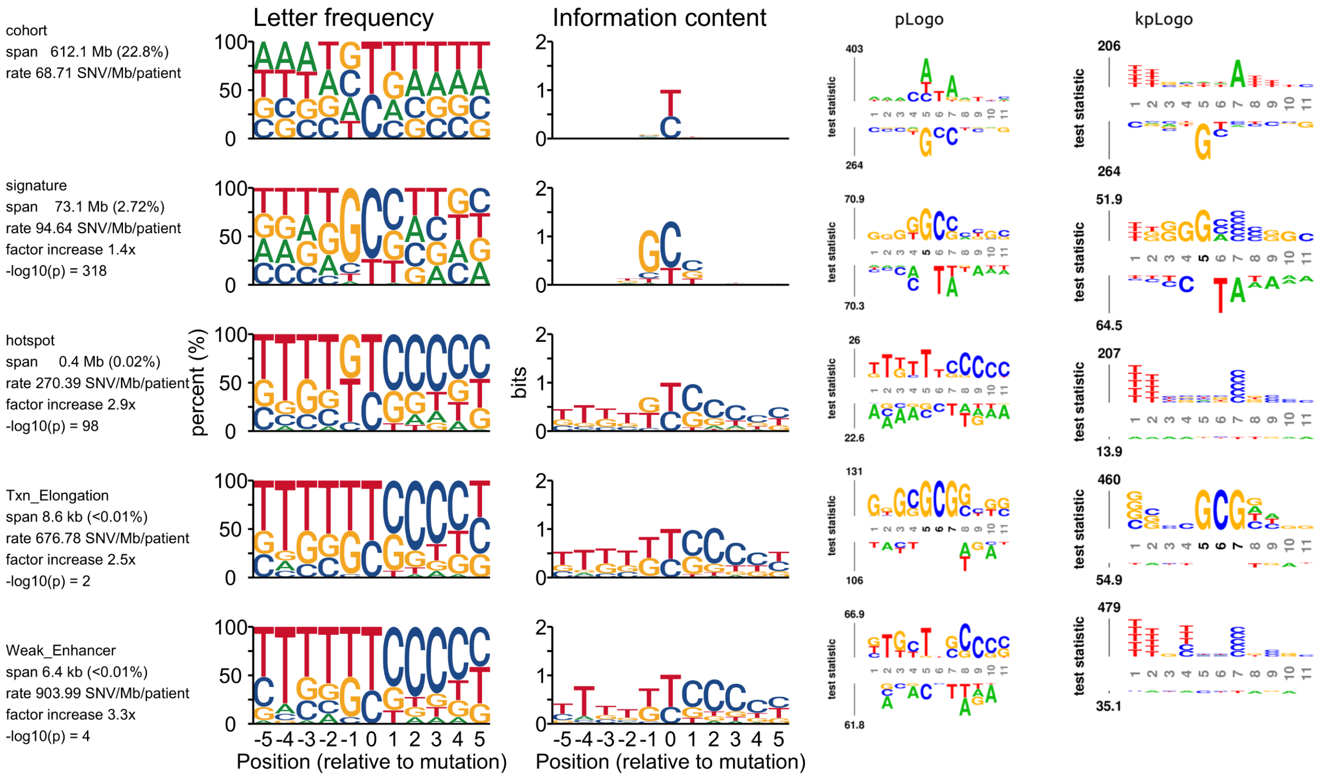
Letter frequency
Information content
pLogo
kpLogo
cohort
span 612.1 Mb (22.8%)
rate 68.71 SNV/Mb/patient
signature
span 73.1 Mb (2.72%)
rate 94.64 SNV/Mb/patient
factor increase 1.4x
-log10(p) = 318
hotspot
span 0.4 Mb (0.02%)
rate 270.39 SNV/Mb/patient
factor increase 2.9x
-log10(p) = 98
Txn_Elongation
span 8.6 kb (<0.01%)
rate 676.78 SNV/Mb/patient
factor increase 2.5x
-log10(p) = 2
Weak_Enhancer
span 6.4 kb (<0.01%)
rate 903.99 SNV/Mb/patient
factor increase 3.3x
-log10(p) = 4
percent (%)
bits
test statistic
Position (relative to mutation)

SBS80

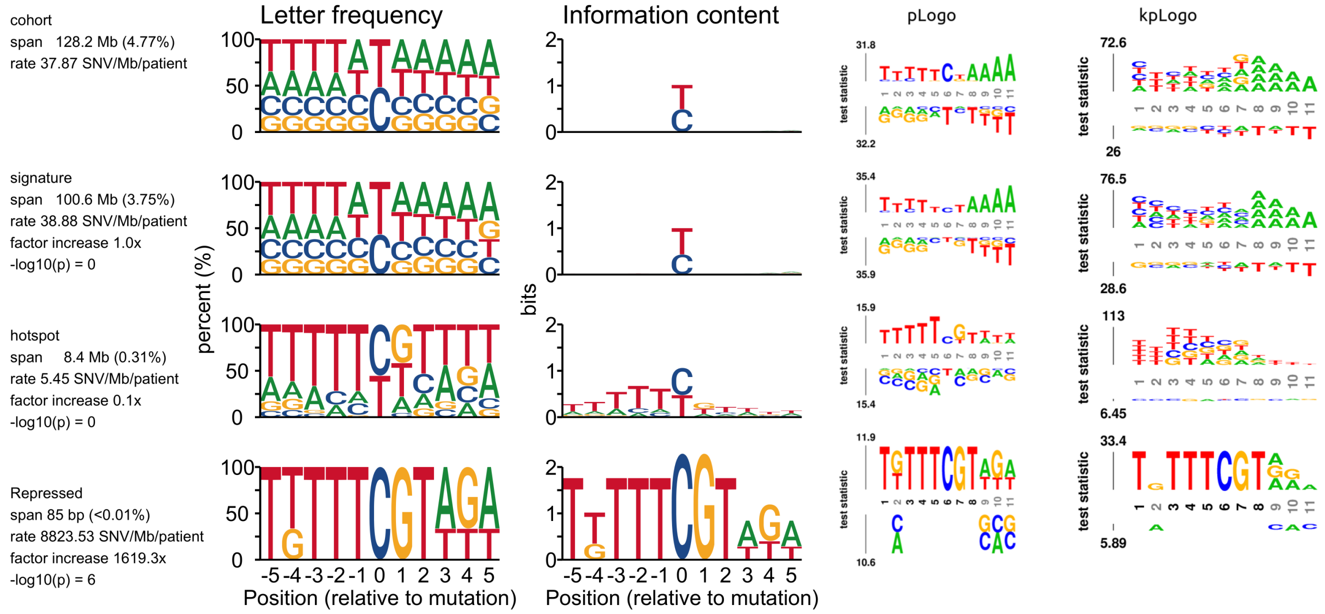

cohort
span 128.2 Mb (4.77%)
rate 37.87 SNV/Mb/patient
signature
span 100.6 Mb (3.75%)
rate 38.88 SNV/Mb/patient
factor increase 1.0x
-log10(p) = 0
hotspot
span 8.4 Mb (0.31%)
rate 5.45 SNV/Mb/patient
factor increase 0.1x
-log10(p) = 0
Repressed
span 85 bp (<0.01%)
rate 8823.53 SNV/Mb/patient
factor increase 1619.3x
-log10(p) = 6
Letter frequency
Information content
pLogo
kpLogo
percent (%)
bits
test statistic
Position (relative to mutation)

Figure S2. Catalog of sequence dependencies.

The letter frequency logo represents the raw frequency of each base in the 11-mers set. The information content logo represents the Kullback-Leibler divergence (bits) of each base compared to the base distribution in the reference genome (chromosome 1-22; A=29.5%; C=20.5%; G=20.5%, T=29.5%). pLogo and kpLogo quantify the surprise of observing a letter given a binomial distribution, where kpLogo only shows the most surprising k-mer ($k \leq 4$) at each position. pLogo and kpLogo use as background the expected base distribution under a given signature, for signature 17b, the background is equivalent to the base distributions in **Fig. 5a** and **Fig. S5**.

Figure S3

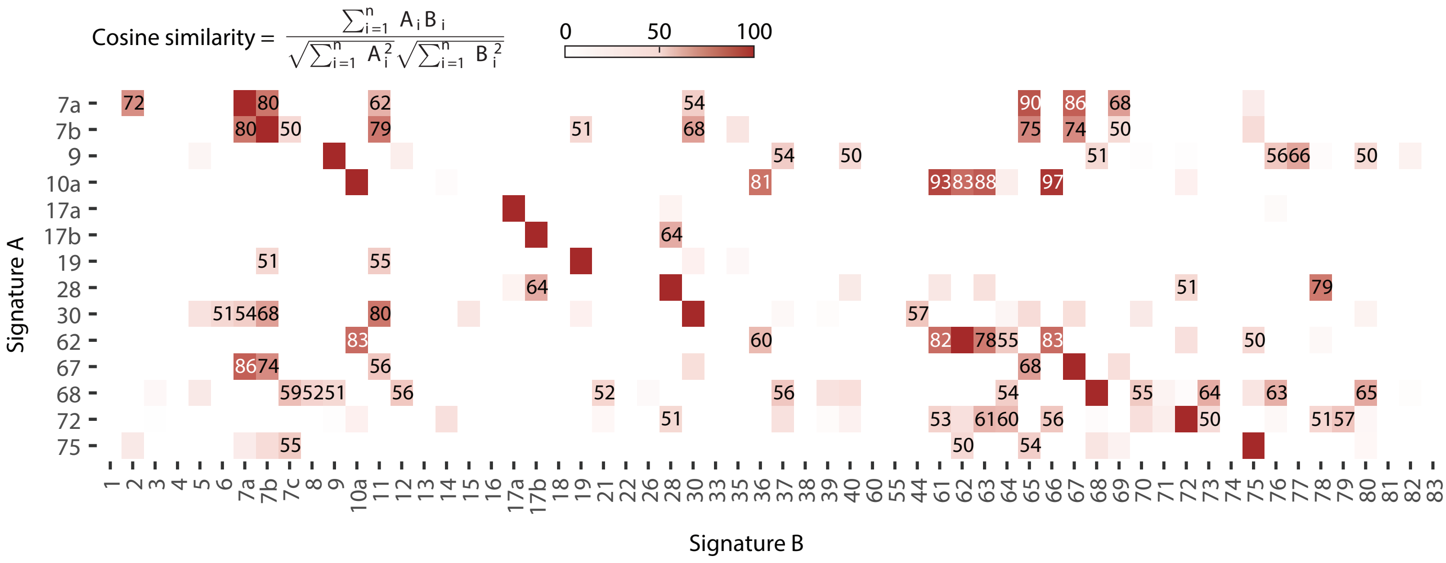

Cosine similarity = $\frac{\sum_{i=1}^{n} A_i B_i}{\sqrt{\sum_{i=1}^{n} A_i^2}\sqrt{\sum_{i=1}^{n} B_i^2}}$
0
50
100
Signature A
Signature B

Figure S3. Cosine similarities between signatures.

Comparison of signatures that are enriched in hotspots (Signature A; y-axis) to all other signatures in the reference set (Signature B; x-axis). The comparison is evaluated using cosine similarity.

Figure S4

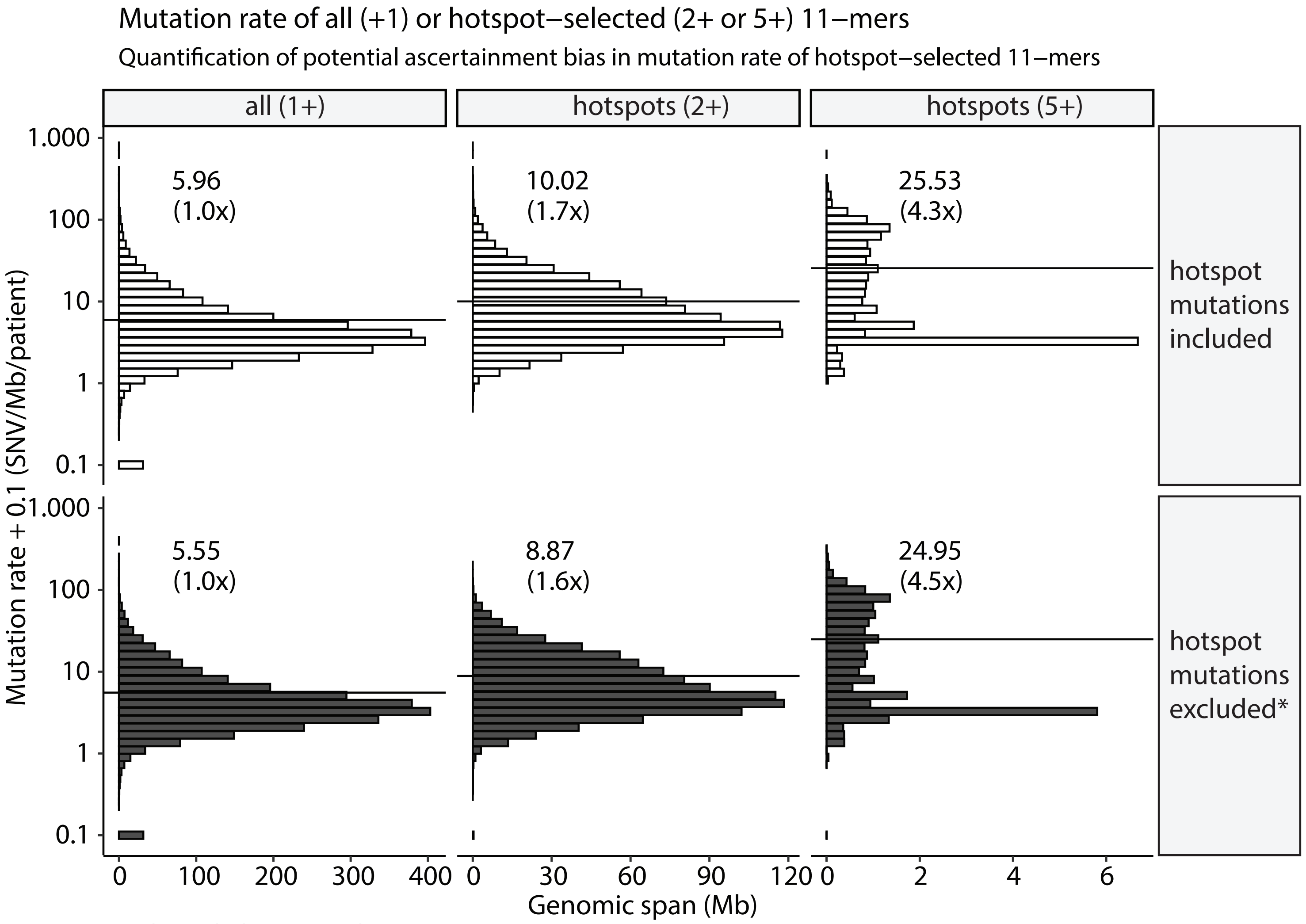

* applies to the bottom panel

panel	excluded from mutation rate	included in mutation rate
left	all hotspot mutations	singletons
middle	all hotspot mutations	singletons
right	hotspot mutations with recurrence ≥5	singletons and mutations with recurrence ≤4

Figure S4. Mutation rates of 11-mers with and without hotspots.

(**Top panels**) 11-mer mutation rates from all SNVs (1+; top left panel), mutation rates of 11-mers with hotspots (2+; top center), and mutation rates of 11-mers with highly recurrent hotspots (5+; top right). (**Bottom panels**) 11-mer mutation rates excluding SNVs from hotspots (1+; bottom left panel), mutation rate of 11-mers with hotspot excluding SNVs from the hotspot (2+; bottom center), mutation rate of 11-mers with highly recurrent hotspots excluding SNVs from highly recurrent hotspots (5+; bottom right).

Figure S5

a

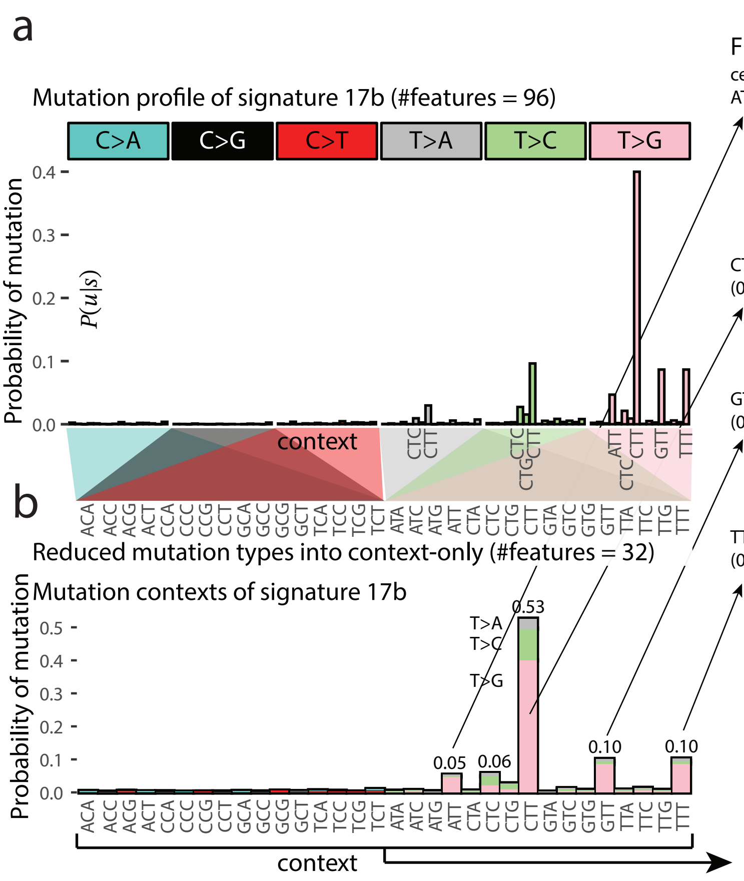

c

$$P(u \text{ in } m|s) = \frac{P(u|s)\,P(m)}{P(trinuc(m))}$$

u	$P(u\|s)$ 10^{-3}	m	$P(m)$ 10^{-5}	$trinuc(m)$	$P(trinuc(m))$		$P(u \text{ in } m\|s)$ 10^{-6}
A[T>A]T	3.5	AAAAATTAAAA	3.3	ATT	0.05	=	2.3
A[T>C]T	2.9	AAAAATTAAAC	0.5	ATT	0.05	=	0.3
...	...	...	...	...	...		
C[T>G]T	400	AAAACTTAAAT	0.3	CTT	0.04	=	30
C[T>A]T	30	AAAACTTAACA	0.2	CTT	0.04	=	1.5
...	...	...	...	...	...		
G[T>C]T	8.5	TTTTGTTTTGT	4.9	GTT	0.03	=	14
G[T>G]T	87	TTTTGTTTTTA	1.6	GTT	0.03	=	46
...	...	...	...	...	...		
T[T>A]T	7.6	TTTTTTTTTTG	18	TTT	0.08	=	17
T[T>C]T	8.2	TTTTTTTTTTT	174	TTT	0.08	=	178

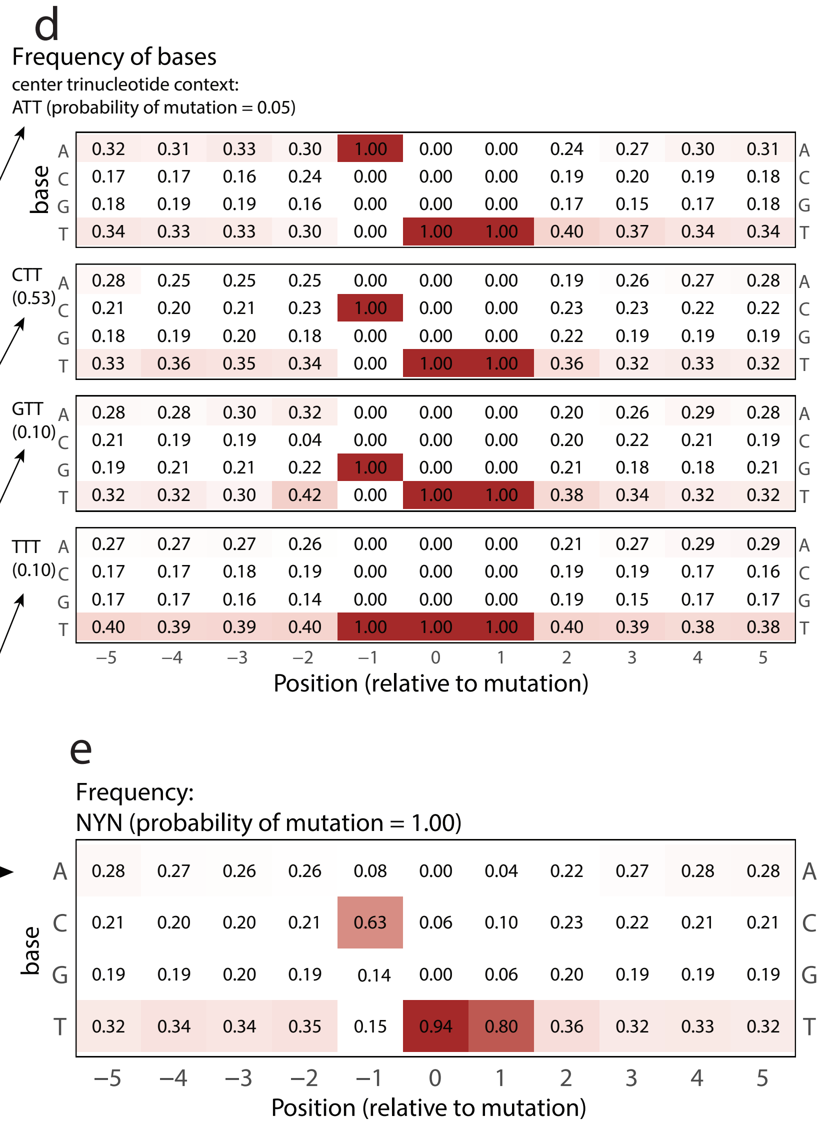

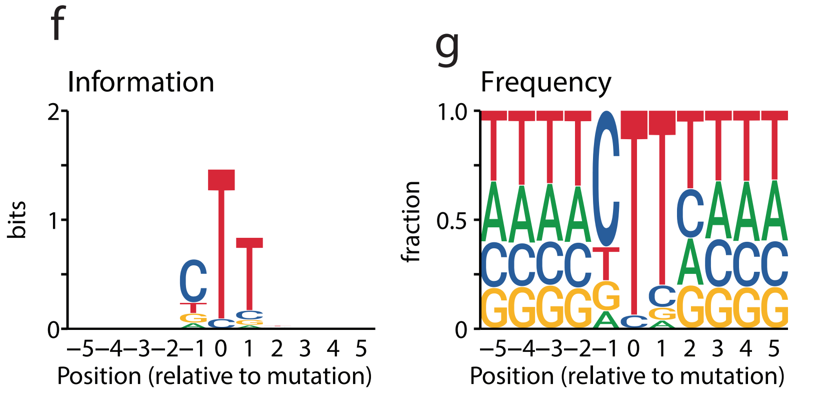

Figure S5. Background 11-mer sets derived from mutational signatures.

(**a**) Reference mutation profile of signature 17b as extracted by SignatureAnalyzer. (**b**) Summarized mutation probability for each trinucleotide context (number of contexts = 32). (**c**) Table of examples of mutation types (u), their probabilities for signature 17b ($P(u|s)$), 11-mers (m), their frequency ($P(m)$), the trinucleotide context associated with the given 11-mer ($trinuc(m)$), the frequency of the trinucleotide ($P(trinuc(m))$), and the probability of a mutation type in an 11-mer for signature 17b ($P(u\ in\ m|s)$). (**d**) Tables of 11-mer base frequencies given fixed trinucleotide contexts. (**e**) Table of 11-mer base frequencies given all 32 trinucleotide contexts. The base frequencies in panel d are used to compute a weighted mean with weights from the summarized mutation probability of each trinucleotide context (panel b). (**f**) Sequence information logo (Kullback-Leibler divergence from background nucleotide distribution: A=29.5%; C=20.5%; G=20.5%, T=29.5%). (**g**) Sequence frequency logo.

Figure S6

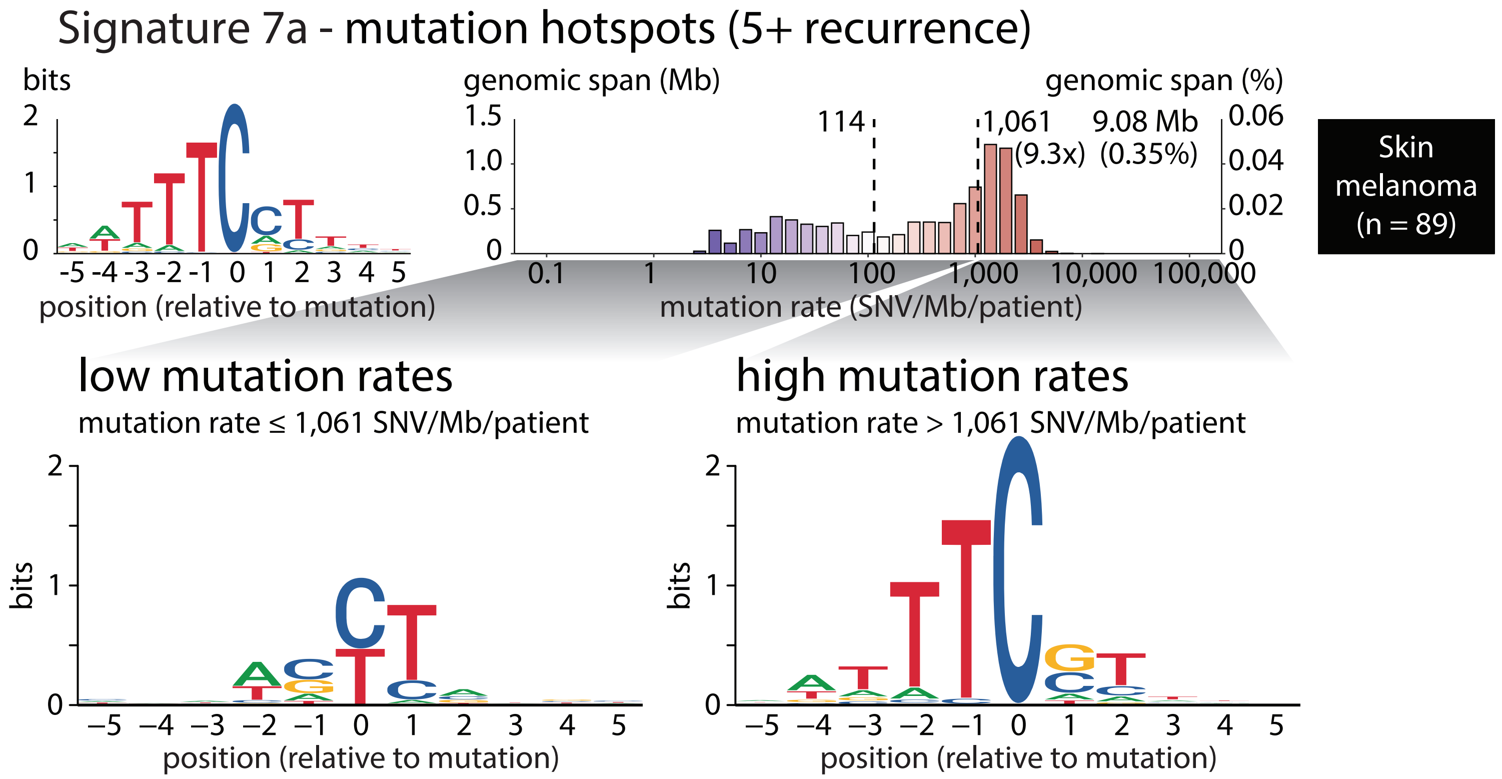
Signature 7a - mutation hotspots (5+ recurrence)
bits
2
1
0
-5 -4 -3 -2 -1 0 1 2 3 4 5
position (relative to mutation)
genomic span (Mb)
genomic span (%)
1.5
1.0
0.5
0
114
1,061
(9.3x)
9.08 Mb
(0.35%)
0.06
0.04
0.02
0
0.1
1
10
100
1,000
10,000
100,000
mutation rate (SNV/Mb/patient)
Skin melanoma (n = 89)
low mutation rates
mutation rate ≤ 1,061 SNV/Mb/patient
high mutation rates
mutation rate > 1,061 SNV/Mb/patient
bits
2
1
0
−5 −4 −3 −2 −1 0 1 2 3 4 5
position (relative to mutation)

Figure S6. UV-signature sequence characteristics across mutation rates.

Signature 7a-assigned 11-mers with hotspots (5+) have a bimodal mutation rate distribution. The sequence characteristics of lowly (equal to or below the mean rate) and highly (above the mean rate) mutated 11-mers are shown as logo plots.

Figure S7

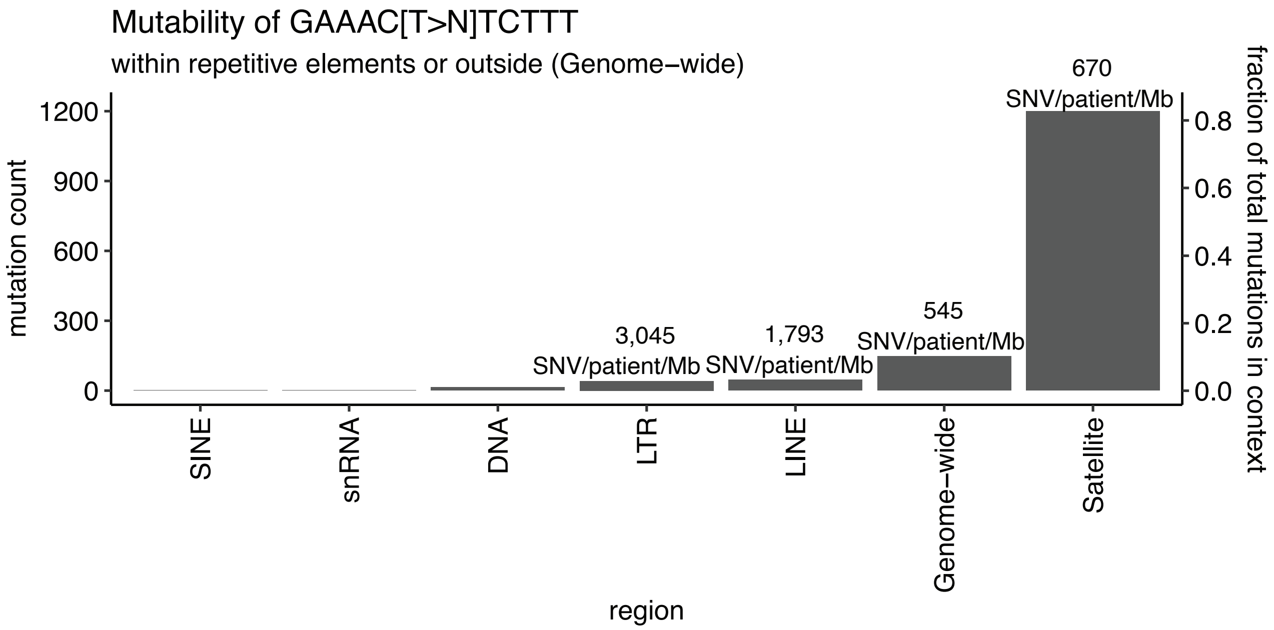

Mutability of GAAAC[T>N]TCTTT
within repetitive elements or outside (Genome−wide)
mutation count
1200
900
600
300
0
fraction of total mutations in context
0.8
0.6
0.4
0.2
0.0
3,045 SNV/patient/Mb
1,793 SNV/patient/Mb
545 SNV/patient/Mb
670 SNV/patient/Mb
SINE
snRNA
DNA
LTR
LINE
Genome−wide
Satellite
region

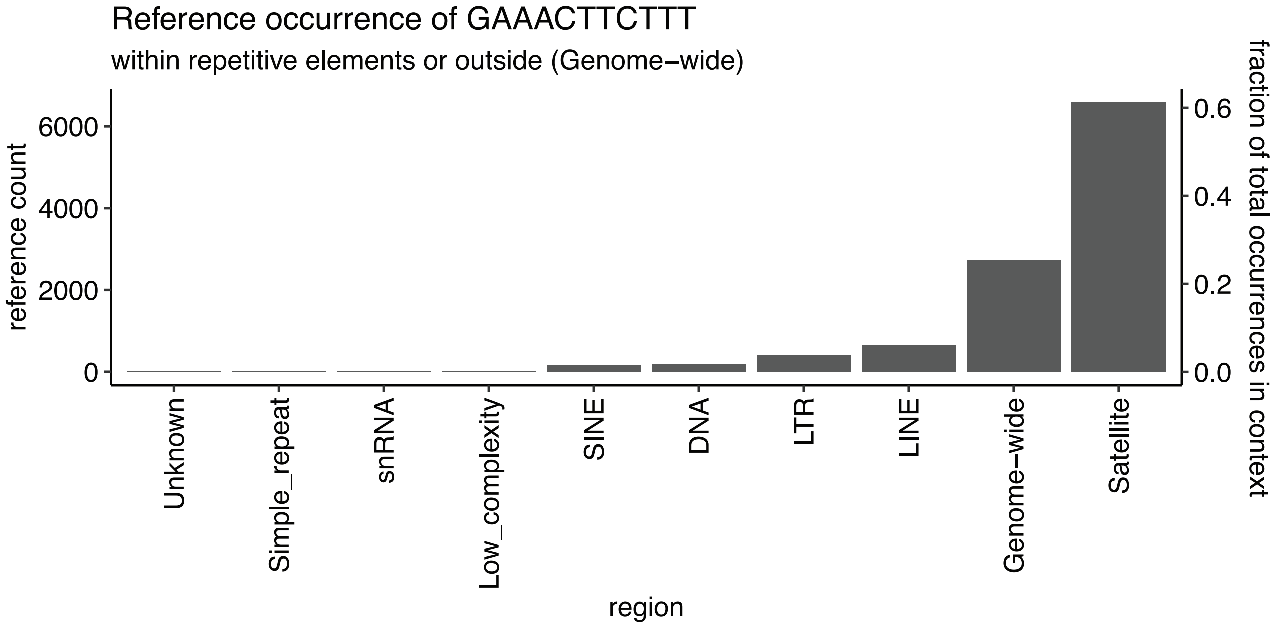

Reference occurrence of GAAACTTCTTT
within repetitive elements or outside (Genome−wide)
reference count
6000
4000
2000
0
fraction of total occurrences in context
0.6
0.4
0.2
0.0
Unknown
Simple_repeat
snRNA
Low_complexity
SINE
DNA
LTR
LINE
Genome−wide
Satellite
region

Figure S7. Characterization of GAAACTTCTTT-sequences in repetitive elements.

(**Top panel**) The number of mutations (y-axis left) in the GAAACTTCTTT contexts across repetitive elements (RepeatMasker) or outside repetitive elements (Genome-wide). (**Bottom panel**) The occurrence (y-axis left) of GAAACTTCTTT instances across repetitive elements (RepeatMasker) or outside repetitive elements (Genome-wide).

Table S1: K-mer statistics

	kmer space				Instances per family					
**k**	**expected**	**observed**	**nullomers**	**SNVs per kmer (expected)**	**min**	**Q1**	**median**	**mean**	**Q3**	**max**
**1**	2	2	0	20,659,025.00	1,100,439,365	1,221,362,933.75	1,342,286,502.50	1,342,286,502.50	1,463,210,071.25	1,584,133,640
**3**	32	32	0	1,291,189.06	11,923,725	67,881,588.50	84,697,455.00	83,892,887.72	105,985,401.25	205,859,180
**5**	512	512	0	80,699.32	147,012	2,952,289.00	5,344,526.50	5,243,304.34	6,967,661.00	36,208,737
**7**	8,192	8,192	0	5,043.71	3,695	63,206.25	292,403.50	327,706.45	457,130.00	11,824,057
**9**	131,072	131,072	0	315.23	54	2,965.75	14,723.00	20,481.65	28,077.25	6,870,011
**11**	2,097,152	2,097,090	62	19.70	0	146.00	608.00	1,280.10	1,637.00	4,674,610
**13**	33,554,432	32,307,152	1,247,280	1.28	0	7.00	23.00	80.01	93.00	3,279,735
**15**	536,870,912	-	-	0.08	-	-	-	5.00	-	-
